# Effects of mind–body exercise on motor function, quality of life, and depressive symptoms in patients with Parkinson’s disease: a systematic review and network meta-analysis

**DOI:** 10.3389/fnagi.2026.1982103

**Published:** 2026-10-28

**Authors:** Haoran Bai, Zhen Chen, Liang Li, Meiqiang Duan, Tonggang Fan, Yinping Zhang

**Affiliations:** 1 College of Wushu, Shanghai University of Sport, Shanghai, China; 2 College of Wushu, Hebei Sport University, Shijiazhuang, Hebei, China

**Keywords:** depression, mind–body exercise, Parkinson’s disease, physical functional performance, quality of life

## Abstract

**Purpose:**

This study used network meta-analysis to compare and rank the effects of different mind–body exercises on motor function, depression, and quality of life in patients with Parkinson’s disease, to inform clinical practice.

**Methods:**

PubMed, the Cochrane Library, Web of Science, Embase, China National Knowledge Infrastructure (CNKI), Wanfang Database, and VIP Database were searched from inception to June 8, 2026, for randomized controlled trials of mind–body exercise interventions in Parkinson’s disease. Network meta-analysis was performed using Stata version 18.0. Direct and indirect evidence was synthesized, and interventions were ranked using the surface under the cumulative ranking curve (SUCRA). Risk of bias and certainty of evidence were assessed using the Cochrane Risk of Bias 2 tool and the Grading of Recommendations Assessment, Development and Evaluation (GRADE) approach, respectively.

**Results:**

A total of 43 studies involving 2,337 patients with Parkinson’s disease were included. For balance measured using the Berg Balance Scale, dance was significantly more effective than no treatment, walking, and usual care. For functional mobility measured using the Timed Up and Go Test, qigong was significantly more effective than tai chi, Dance, stretching, no treatment, and usual care. For overall motor function measured using the Unifed Parkinson’s Disease rating Scale, Part III, yoga was significantly more effective than usual care and no treatment. For depressive symptoms measured using the Hamilton Depression Scale, tai chi was significantly more effective than qigong, no treatment, walking, and usual care. For health-related quality of life, as measured by the Parkinson’s Disease Questionnaire, qigong was significantly more effective than usual care.

**Conclusion:**

Moderate-certainty evidence supported benefits of dance for balance and qigong for functional mobility and quality of life compared with usual care, but superiority over other exercise modalities remains uncertain. For overall motor function, the current evidence is insufficient to identify a clearly superior mind–body exercise intervention, and the findings for Pilates and yoga remain uncertain, with the significant effects of yoga not proving robust in sensitivity analysis. Tai chi showed potential benefits for depressive symptoms; however, this finding should be interpreted cautiously because it was based on a limited number of studies and relied substantially on indirect comparisons.

**Systematic review registration:**

PROSPERO, CRD420261418269.

## 1 Introduction

Parkinson’s disease (PD) is the most prevalent neurodegenerative disorder worldwide and one of the leading causes of motor dysfunction and disability in older adults (Dorsey and Bloem, 2018; Karikari et al., 2018). Its principal manifestations include motor symptoms such as resting tremor, bradykinesia, muscular rigidity, and postural instability (Jankovic, 2008), as well as non-motor symptoms such as autonomic dysfunction and psychiatric disturbances (Gunawardana et al., 2026). Patients may also experience chronic pain, apathy, sleep disturbances, and other symptoms that impair quality of life (De Bruin et al., 2026), further increasing the burden of the disease. Epidemiological data indicate that approximately 11.8 million people worldwide were living with Parkinson’s disease in 2021 (Luo et al., 2024), and the global burden of the disease is projected to increase substantially by 2040 (Dorsey et al., 2007), which poses a significant public health risk. At present, pharmacological treatment remains the mainstay of PD management, with clinical practice relying predominantly on dopaminergic medications to control both motor and non-motor symptoms (Grabli et al., 2012; Bloem et al., 2021).

Nevertheless, pharmacological treatment has limited efficacy for some manifestations of Parkinson’s disease and cannot halt disease progression (Aydın et al., 2026). Moreover, long-term and intensive medication use may also increase physiological and financial burdens and adversely affect quality of life. Effective nonpharmacological therapies are therefore urgently needed. Exercise therapy has emerged as one of the most promising nonpharmacological approaches for Parkinson’s disease. Evidence suggests that different forms of exercise may attenuate neuroinflammation, oxidative stress, and mitochondrial dysfunction (Volpe et al., 2017; Niering et al., 2025); improve balance, alleviate gait rigidity, and enhance mood and sleep quality (Rayapuraju et al., 2026).

Mind–body exercise integrates breath regulation, attentional control, and psychological awareness into a unified form of physical activity (Wen et al., 2025). This combined physical and mental approach may be particularly relevant to Parkinson’s disease (PD), in which motor impairment and mood disturbances may involve partly overlapping pathophysiological mechanisms, including dopaminergic dysfunction in motor and limbic circuits (Remy et al., 2005). By incorporating both physical and mental components, mind–body exercise may help address these coexisting symptoms (Kwok et al., 2019). Multiple studies have shown that mind–body exercise helps alleviate freezing of gait and fear of falling in patients with PD (Ernst et al., 2024; Li et al., 2026), and it has shown potential benefits for non-motor symptoms such as mood, cognition, and sleep (Wang et al., 2021; Zhang et al., 2022). Although mind–body exercise may not always produce the greatest improvement in motor symptoms compared with other forms of exercise, it is generally easier to learn and perform and requires less physical exertion, making it an accessible intervention option for people with PD.

Previous pairwise meta-analyses have examined mind–body exercise in PD, but their focus on individual comparisons limits the simultaneous evaluation of multiple modalities within a common analytical framework (Wang et al., 2021; Jin et al., 2019; Song et al., 2017). Several published network meta-analyses have included aerobic, resistance, balance, and mind–body exercises within a single network, providing broad comparisons across exercise types rather than focusing specifically on mind–body modalities (Ernst et al., 2024; Zeng et al., 2026; Zhan et al., 2026). A network meta-analysis published in 2022 specifically compared mind–body exercise modalities for functional performance and quality of life in PD, treating each modality as a separate intervention node (Mustafaoglu et al., 2022). This study provided a foundation for comparative effectiveness assessment, but the range of outcomes examined was limited. As new evidence in exercise rehabilitation accumulates, an updated synthesis covering a broader range of outcomes is needed to clarify the relative benefits and clinical relevance of these interventions. Accordingly, this study integrates direct and indirect evidence from the latest randomized controlled trials to compare the effects of Tai chi, Qigong, Yoga, Dance, and Pilates on motor function, depressive symptoms, and quality of life in patients with Parkinson’s disease. Relative benefits and clinical relevance are assessed in the context of treatment rankings, applicable minimal clinically important difference thresholds, and certainty of evidence to inform the choice of exercise interventions and future research in PD.

## 2 Materials and methods

### 2.1 Study registration

This systematic review and network meta-analysis focused on mind–body exercise interventions, strictly adhering to the Preferred Reporting Items for Systematic Reviews and Meta-Analyses (PRISMA) statement and its extension for network meta-analyses (PRISMA, 2026). The review protocol was prospectively registered on the international systematic review registry platform PROSPERO (Registration Number CRD420261418269; Registration Date: June 8, 2026) (PROSPERO, 2026). Sensitivity analyses using a random-effects model and excluding studies at high risk of bias were not prespecified in the registered protocol and were conducted to assess the robustness of the findings.

### 2.2 Search strategy

Four international databases (PubMed, the Cochrane Library, Web of Science, and Embase) and three Chinese databases (CNKI, Wanfang Database, and VIP Database) were searched from inception to June 8, 2026. Core concepts were defined using the PICO framework, and searches employed a combination of “subject headings + free-text terms + synonyms/acronyms.” Additionally, after the preliminary set of eligible studies had been established, the reference lists and “similar articles” records of all included studies were screened to minimize omissions and improve comprehensiveness. Database-specific thesauri were also used to optimize each search strategy. Detailed search strategies are provided in Supplementary Appendix 1.

### 2.3 Eligibility criteria

Inclusion and exclusion criteria were established in accordance with the PICOS principles (Li et al., 2025).

Inclusion criteria: (1) Population: adult patients with Parkinson’s disease aged ≥ 18 years, irrespective of disease stage, without comorbidities that could interfere with outcome assessment; (2) Intervention: a mind–body exercise intervention, including tai chi, qigong, yoga, dance, or Pilates; (3) comparison: conventional psychotropic medication, conventional physical exercise, or standard care; (4) Outcomes: at least one of the following outcomes with complete measurement data: motor function assessed using the Berg Balance Scale, Timed Up and Go Test, or Unifed Parkinson’s Disease rating Scale, Part III; quality of life assessed using the Parkinson’s Disease Questionnaire; or depressive symptoms assessed using the Hamilton Depression Scale; (5) Study design: a full-text randomized controlled trial published in Chinese or English with sufficient data for effect-size extraction.

Exclusion criteria: (1) non-RCT studies, reviews, meta-analyses, conference abstracts, theses, or unpublished research reports; (2) participants aged < 18 years; (3) serious comorbidities, such as other psychiatric disorders, cardiovascular or cerebrovascular disease, or severe psychological disorders, that could affect the study results; (4) interventions combining mind–body exercise with other forms of exercise when the effect of the exercise modality could not be isolated, or studies without a clearly defined exercise protocol; (5) duplicate publications; (6) incomplete study data for which key information could not be obtained or supplemented. Definitions of each intervention and outcome are provided in Supplementary Appendices 2, 3.

### 2.4 Data extraction

Two reviewers independently extracted key information from the included randomized controlled trials using a prespecified standardized form in Microsoft Excel. The extracted information comprised: (1) basic study information: first author, publication year, country of study, and study design; (2) participant characteristics: sample size in each group, age, sex distribution, Hoehn and Yahr stage, and baseline BBS, TUG, UPDRS-III, HAMD, and PDQ-39 scores; (3) intervention and control measures: mind–body exercise type, total intervention duration, weekly frequency, duration of each session, and the type and implementation details of the comparator; (4) outcome measure data: postintervention raw scores for the BBS, TUG, UPDRS-III, HAMD, and PDQ-39; and (5) information related to risk-of-bias assessment: random-sequence generation, allocation concealment, blinding, completeness of outcome data, selective reporting, and other potential sources of bias. After extraction, the two reviewers cross-checked the data. Disagreements were resolved through discussion or adjudication by a third reviewer.

### 2.5 Quality assessment

The risk of bias in the included RCTs was assessed according to the recommendations of the Cochrane Handbook for Systematic Reviews of Interventions (Higgins et al., 2026), using the updated Cochrane Risk of Bias Assessment Tool for Randomized Trials (RoB 2). Two reviewers independently conducted risk-of-bias assessments. Disagreements were resolved through discussion or arbitration by a third senior researcher. The RoB 2 signaling questions and domain-specific algorithms were used to inform judgments for each domain and the overall risk of bias. RoB 2 assessments were checked separately for each included outcome result. Judgments were categorized as “Low risk,” “Some concerns,” or “High risk.” The risk of bias assessment includes the following domains: bias arising from the randomization process, deviations from intended interventions, missing outcome data, outcome measurement, and selection of the reported result. Additionally, the GRADE approach for network meta-analysis was used to assess the certainty of each network estimate. This system systematically grades the quality of evidence based on the study design, risk of bias, inconsistency, precision, and publication bias. Direct and indirect evidence were evaluated separately, with further consideration of intransitivity, incoherence, and network imprecision. Final certainty was rated as high, moderate, low, or very low.

### 2.6 Data synthesis and analysis

This study conducted a network meta-analysis within a frequentist statistical framework. All statistical analyses were performed using Stata software (version 18.0). This method integrates direct evidence from RCTs with indirect evidence derived from common comparators, enabling simultaneous estimation of relative treatment effects across all exercise interventions. Network plots were constructed to visualize the structure of the evidence. In each plot, each exercise intervention is represented as a node, and lines connecting the nodes indicate the existence of direct comparative studies. Node size was proportional to the number of participants receiving the intervention, while line thickness reflected the number of randomized controlled trials informing that comparison. The plots therefore provided a visual overview of network connectivity and the evidence base supporting each comparison.

Qigong forms were grouped into a single treatment node based on their shared integration of movement or posture, breathing regulation, and attentional focus. This category-level definition reflected the review’s aim of comparing broad mind–body exercise modalities rather than individual Qigong forms and did not imply equivalent effects across forms.

In this study, all outcomes were continuous variables. A fixed-effect model was used as a parsimonious primary analysis given the sparse evidence for several treatment comparisons, under the assumption of a common relative effect within each comparison, and effect sizes were expressed as mean differences (MDs) with 95% confidence intervals (CIs). The relative effectiveness of each exercise intervention was ranked using SUCRA. SUCRA values range from 0 to 100%, with higher values indicating a greater probability that an intervention is among the most effective.

To assess the robustness of the findings and the influence of model choice, *post hoc* sensitivity analyses were conducted for outcomes showing moderate between-study heterogeneity. First, the effect model was changed to a random-effects model. If a material difference was observed—defined as a change in the highest-ranked intervention or a loss or reversal of statistical significance for a comparison that was significant in the primary fixed-effect analysis—studies at high risk of bias were further excluded and the fixed-effect analysis was repeated to evaluate the stability of the primary findings. Results were considered robust when the sensitivity analyses were consistent with the primary analysis; any material discrepancies were reported and discussed.

The transitivity assumption was assessed by comparing the distribution of clinically relevant potential effect modifiers across direct treatment comparisons. The prespecified effect modifiers included participant age, Hoehn–Yahr stage, baseline severity of the corresponding outcome, disease duration, intervention duration and frequency, and medication status. The distributions of these variables were compared descriptively across the available direct comparisons. Transitivity was considered plausible when no substantial systematic imbalance in these effect modifiers was identified across comparisons. However, residual intransitivity was acknowledged when important between-comparison differences remained or when relevant information was substantially missing.

Agreement between direct and indirect evidence was assessed using both global and local approaches. Globally, an inconsistency model was used to evaluate consistency across the entire network. Locally, node-splitting analysis and loop-specific inconsistency tests were used to examine differences between direct and indirect evidence for individual comparisons. Comparison-adjusted funnel plots were used to assess publication bias and small-study effects. All statistical tests were two-sided, with *p* < 0.05 considered statistically significant.

## 3 Results

### 3.1 Study selection

This study followed the PRISMA guidelines; seven databases were systematically searched: PubMed (157 records), Web of Science (851 records), the Cochrane Library (545 records), Embase (271 records), CNKI (230 records), Wanfang Database (229 records), and VIP Database (173 records), yielding a total of 2,456 records. After duplicates were removed, 921 records underwent title and abstract screening, and 321 articles were assessed in full text. Following rigorous eligibility assessment, 43 randomized controlled trials were included in the systematic review (Supplementary Appendix 4: Dorsey and Bloem, 2018; Karikari et al., 2018; Jankovic, 2008; Gunawardana et al., 2026; De Bruin et al., 2026; Luo et al., 2024; Dorsey et al., 2007; Grabli et al., 2012; Bloem et al., 2021; Aydın et al., 2026; Volpe et al., 2017; Niering et al., 2025; Rayapuraju et al., 2026; Wen et al., 2025; Remy et al., 2005; Kwok et al., 2019; Ernst et al., 2024; Li et al., 2026; Wang et al., 2021; Zhang et al., 2022; Jin et al., 2019; Song et al., 2017; Zeng et al., 2026; Zhan et al., 2026; Mustafaoglu et al., 2022; PRISMA, 2026; PROSPERO, 2026; Li et al., 2025; Higgins et al., 2026; Ni et al., 2016; Mehdizadeh et al., 2025; Chen et al., 2020; Sharp and Hewitt, 2014; Beitz, 2014; Huang and He, 2016; Sohn et al., 2018; Federici et al., 2005; Hackney and McKee, 2014; Taghizadeh et al., 2025; Wang et al., 2019; Tu and Liao, 2014; Zheng et al., 2021; Ghai et al., 2018). The detailed study-selection process is shown in Figure 1.

**Figure 1:**
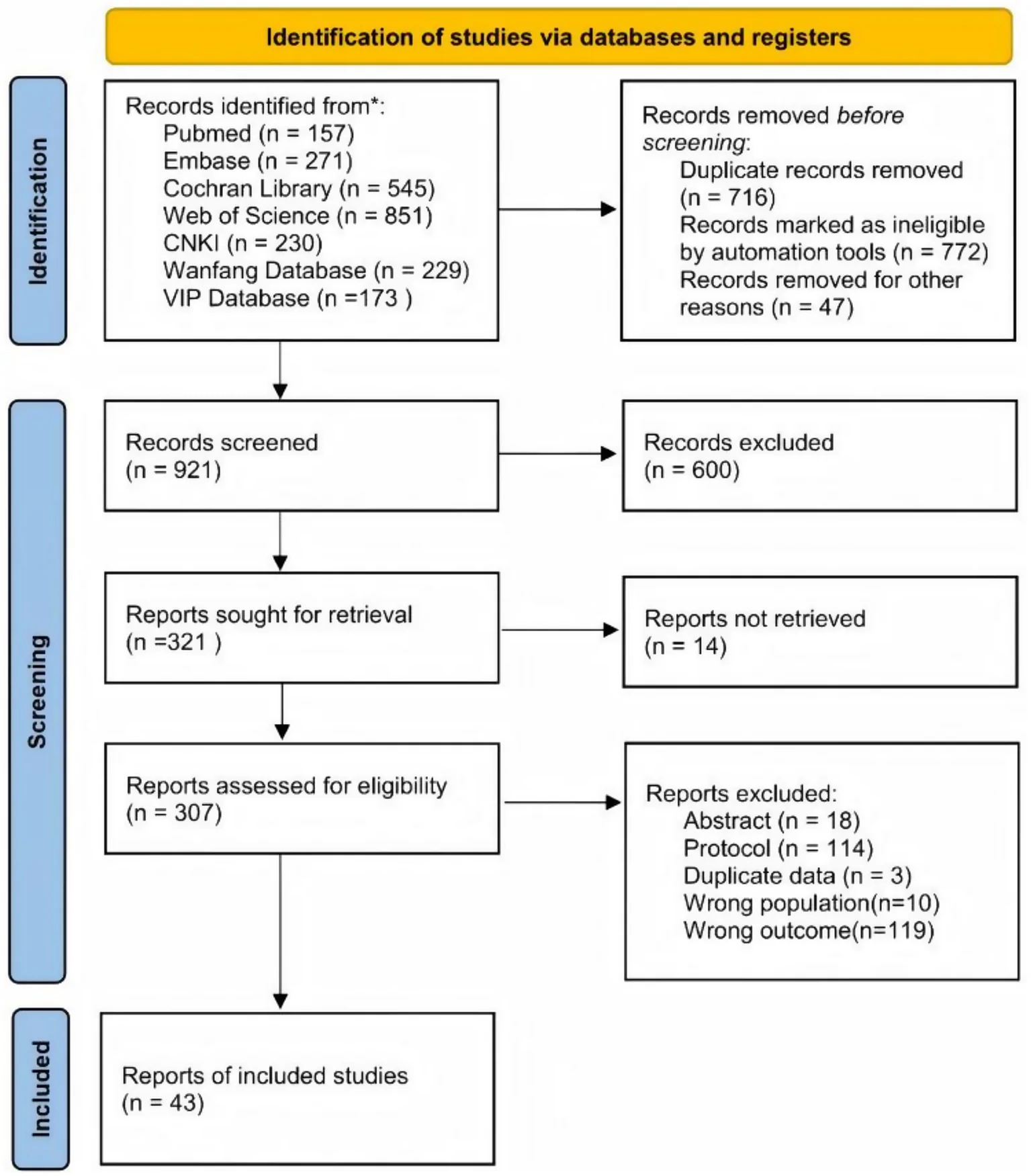
Flow chart of literature search and study selection.

### 3.2 Study and participant characteristics

The 43 included studies were published between 2011 and 2026 and comprised 16 Chinese-language and 27 English-language articles. Studies were conducted in 10 countries: China (27 studies), the United States (5), Italy (3), South Korea (2), the United Kingdom (1), Brazil (1), Spain (1), India (1), Germany (1), and Canada (1). A total of 2,337 patients with Parkinson’s disease were included, of whom 1,210 were assigned to intervention groups and 1,127 to comparator groups. The interventions comprised five forms of mind–body exercise: Tai chi (15 studies), Qigong (11), Yoga (8), Dance (7), and Pilates (2). Qigong and dance encompass multiple subtypes, with intervention protocols varying across studies; these subtypes were pooled into their respective treatment nodes for analysis. Comparator groups received standard care, conventional exercises such as stretching or walking, or maintained their baseline activity level without an exercise intervention (no treatment). Intervention programs ranged from 4 to 24 weeks in duration, with session frequencies of 1–14 per week and individual sessions lasting 20–90 min. TUG outcomes were reported in 25 studies, UPDRS-III outcomes in 21, BBS outcomes in 19, PDQ-39 outcomes in 17, and HAMD outcomes in 7. Additional study characteristics are presented in Supplementary Appendix 4.

### 3.3 Risk of bias

The revised Cochrane Risk of Bias tool (RoB 2) was used to assess five domains in the 43 included studies. Domain-level and overall judgments were identical across outcomes within each study and are therefore presented together. The overall risk of bias was “Low risk” (13 studies, 30.2%), “Some concerns” (29 studies, 67.4%), and “High risk” (1 study, 2.3%) (Supplementary Appendix 5). Common sources of concern included incomplete reporting relevant to the selection of reported results and, in some studies, issues related to deviations from intended interventions, missing outcome data, or outcome measurement. A summary of the revised assessments is shown in Figure 2.

**Figure 2:**
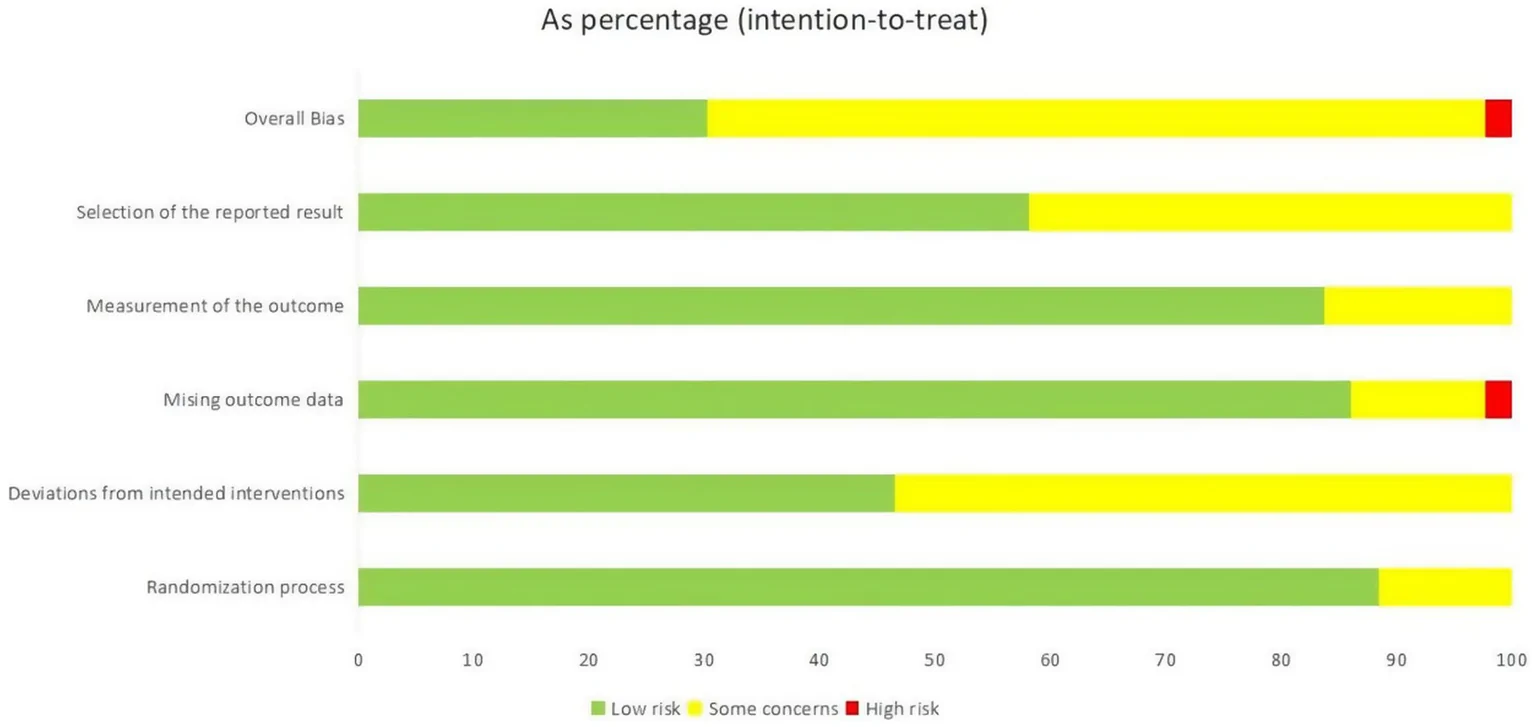
Distribution of risk of bias across included studies, as assessed by the Cochrane RoB 2 Risk of Bias tool.

### 3.4 Assessment of transitivity

The distributions of major potential effect modifiers were examined across direct treatment comparisons. Age and Hoehn–Yahr stage showed substantial overlap across comparisons, with most trials enrolling participants with mild-to-moderate Parkinson’s disease. Disease duration and baseline outcome severity were more variable, although no clear systematic separation of these characteristics across treatment comparisons was observed. Considerable variation was present in intervention duration and training frequency. Medication-related conditions also differed across studies, with some trials assessing outcomes in the ON-medication state, some after medication withdrawal in the OFF state, and others without clearly reporting the assessment state. Overall, no major systematic violation of the transitivity assumption was identified; however, residual intransitivity, particularly related to medication status and intervention dose, could not be excluded. The detailed distributions of the potential effect modifiers are presented in Supplementary Appendix 6.

### 3.5 Assessment of inconsistency and publication bias

The global inconsistency test detected no statistically significant evidence of inconsistency for the BBS (χ^2^ = 0.07, df = 2, *p* = 0.966), TUG (χ^2^ = 8.42, df = 8, *p* = 0.393), UPDRS-III (χ^2^ = 2.25, df = 4, *p* = 0.691), or PDQ-39 (χ^2^ = 3.25, df = 4, *p* = 0.516). Node-splitting analysis further showed no statistically significant disagreement between direct and indirect evidence for the available comparisons (*p* > 0.05), supporting the global findings (Supplementary Appendix 7). Assessment of heterogeneity within closed loops indicated low between-study heterogeneity for the BBS (τ^2^ = 0.76) and TUG (τ^2^ = 0.77), and moderate heterogeneity for the UPDRS-III (τ^2^ = 11.83) and PDQ-39 (τ^2^ = 22.37) (Supplementary Appendix 8). Comparison-adjusted funnel plots showed that the scatter points for each outcome were approximately symmetrical around the central line (Supplementary Appendices 9.1–9.5). However, funnel-plot assessment may have limited ability to detect publication bias or small-study effects for outcomes supported by sparse evidence, particularly the Hamilton Depression Rating Scale (HAMD).

### 3.6 Analysis of outcomes

#### 3.6.1 BBS

Nineteen studies involving 969 participants evaluated the BBS. The comparison network included five mind–body exercise interventions (Figure 3): tai chi (*n* = 9 studies), qigong (Baduanjin and Wuqinxi; *n* = 5), dance (*n* = 3), yoga (*n* = 1), and Pilates (*n* = 1). Usual care served as a central node, directly connected to tai chi, qigong, yoga, dance, and Pilates through direct evidence.

**Figure 3:**
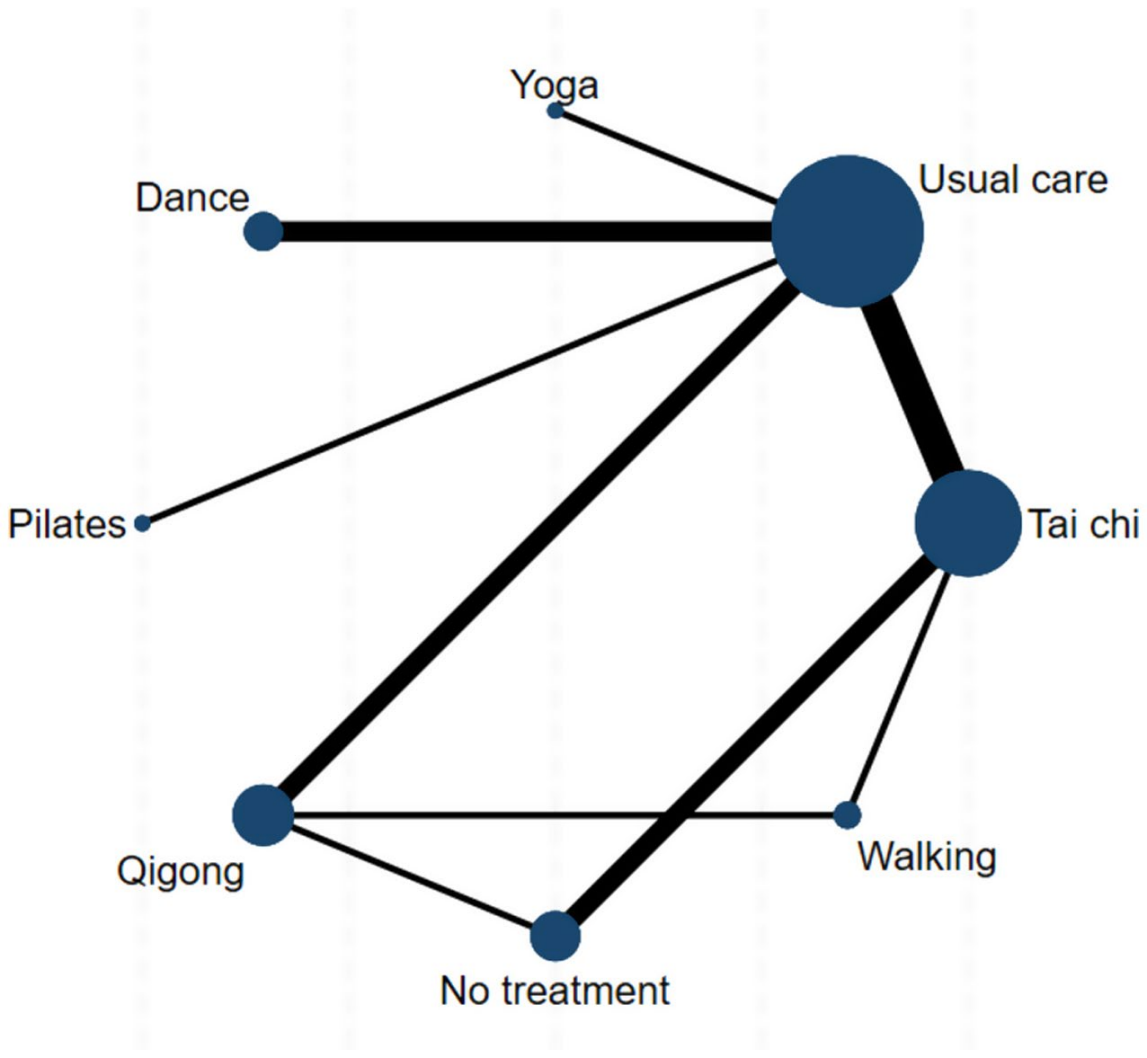
Network structure of BBS in patients with Parkinson’s disease.

The league table of all pairwise comparisons (Supplementary Appendix 10.1) showed that dance was significantly more effective than no treatment [MD = 3.39; 95% CI (0.32, 6.46)], walking [MD = 3.67; 95% CI (0.58, 6.75)], and usual care [MD = 5.20; 95% CI (2.70, 7.69)]. Tai chi was significantly more effective than no treatment [MD = 2.48; 95% CI (1.15, 3.81)], walking [MD = 2.75; 95% CI (0.93, 4.57)], and usual care [MD = 4.28; 95% CI (3.14, 5.42)]. Qigong was significantly more effective than walking [MD = 1.70; 95% CI (0.08, 3.31)] and usual care [MD = 3.23; 95% CI (2.12, 4.33)]. No other pairwise comparisons between exercise interventions were statistically significant, as their 95% CIs crossed zero.

SUCRA rankings for the BBS differed across intervention types (Supplementary Appendix 11.1). Dance (SUCRA = 90.7%) and tai chi (SUCRA = 82.8%) ranked highest. Qigong also ranked favorably (SUCRA = 62.1%), while Pilates showed a moderate SUCRA ranking (SUCRA = 58.5%). Yoga (SUCRA = 40.5%), no treatment (SUCRA = 34.1%), and walking (SUCRA = 28.2%) ranked lower, and usual care had the lowest SUCRA value (3.0%). The dance ranking reflects pooled evidence across dance styles and does not establish the relative efficacy of individual styles.

#### 3.6.2 TUG

Twenty-five studies involving 1,387 participants evaluated the TUG. The comparison network included five mind–body exercise interventions (Figure 4): tai chi (*n* = 9 studies), qigong (Baduanjin, Wuqinxi, and Health Qigong; *n* = 5), dance (*n* = 5), yoga (*n* = 5), and Pilates (*n* = 2). Usual care served as a central node, directly connected to tai chi, qigong, yoga, dance, and Pilates through direct evidence.

**Figure 4:**
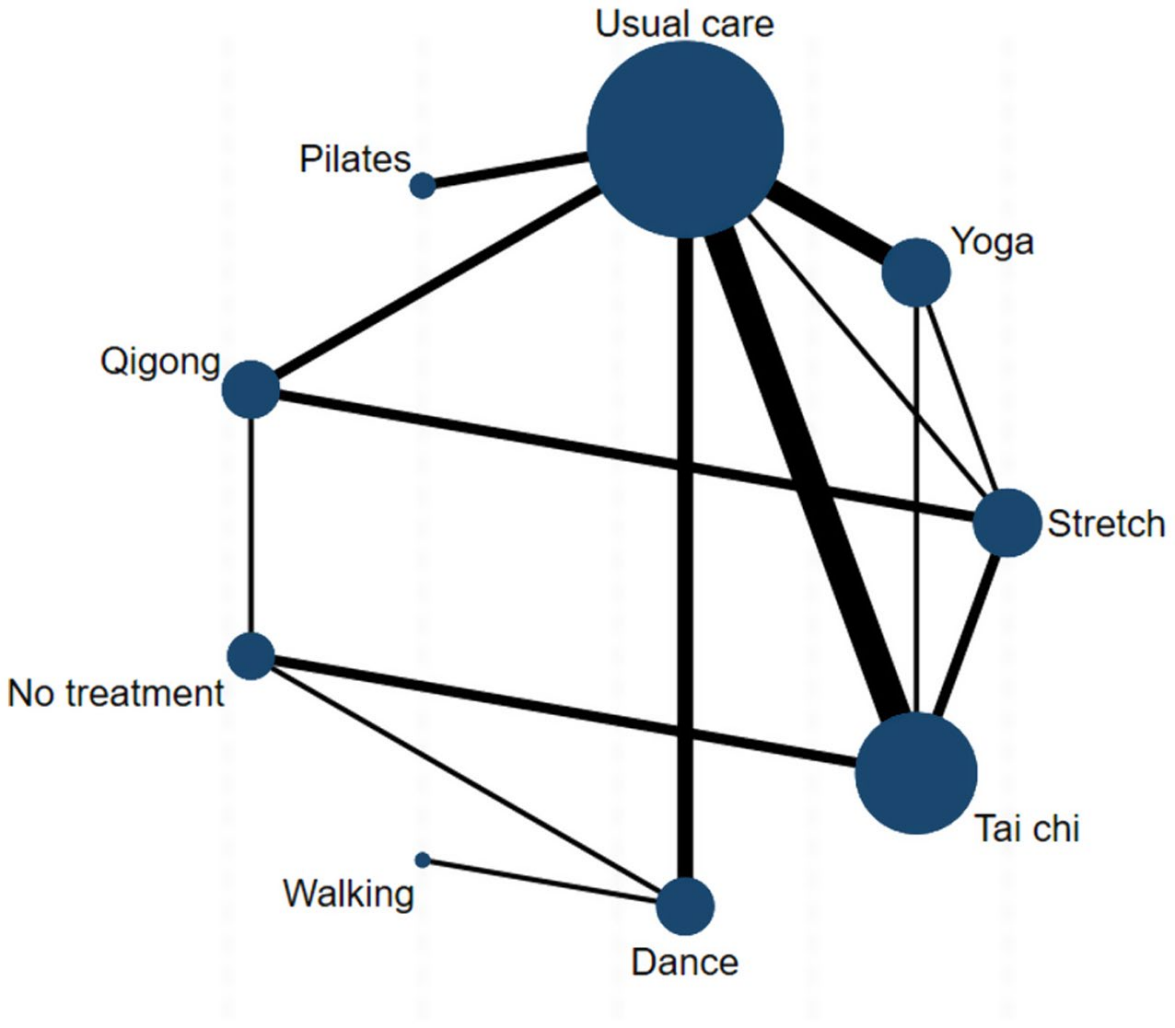
Network structure of TUG in patients with Parkinson’s disease.

Pairwise comparisons (Supplementary Appendix 10.2) showed that qigong was significantly more effective than tai chi [MD = −1.59; 95% CI (−2.82, −0.36)], dance [MD = −1.85; 95% CI (−3.35, −0.36)], stretching [MD = −1.86; 95% CI (−3.10, −0.62)], no treatment [MD = −2.76; 95% CI (−4.17, −1.35)], and usual care [MD = −2.81; 95% CI (−3.91, −1.70)]. Yoga [MD = −1.56; 95% CI (−2.55, −0.57)] and tai chi [MD = −1.22; 95% CI (−2.05, −0.38)] were also significantly more effective than usual care. No other pairwise comparisons between exercise interventions were statistically significant, as their 95% CIs crossed zero.

SUCRA rankings for the TUG differed across intervention types (Supplementary Appendix 11.2). Qigong ranked highest (SUCRA = 96.8%). Yoga (SUCRA = 70.9%) and Pilates (SUCRA = 63.9%) also ranked favorably, while tai chi (SUCRA = 58.6%) and dance (SUCRA = 49.5%) showed moderate SUCRA rankings. Stretching (SUCRA = 46.8%) and walking (SUCRA = 33.5%) ranked lower. No treatment (SUCRA = 16.9%) and usual care (SUCRA = 13.0%) had the lowest SUCRA values. The qigong ranking reflects pooled evidence across qigong subtypes and does not establish the relative efficacy of individual subtypes.

#### 3.6.3 UPDRS-III

Twenty-one studies involving 1,082 participants evaluated the UPDRS-III. The comparison network included five mind–body exercise interventions (Figure 5): tai chi (*n* = 9 studies), qigong (Baduanjin, Wuqinxi, Yijinjing, and Health Qigong; *n* = 6), dance (*n* = 3), yoga (*n* = 2), and Pilates (*n* = 1). Usual care served as a central node, directly connected to tai chi, qigong, yoga, dance, and Pilates through direct evidence.

**Figure 5:**
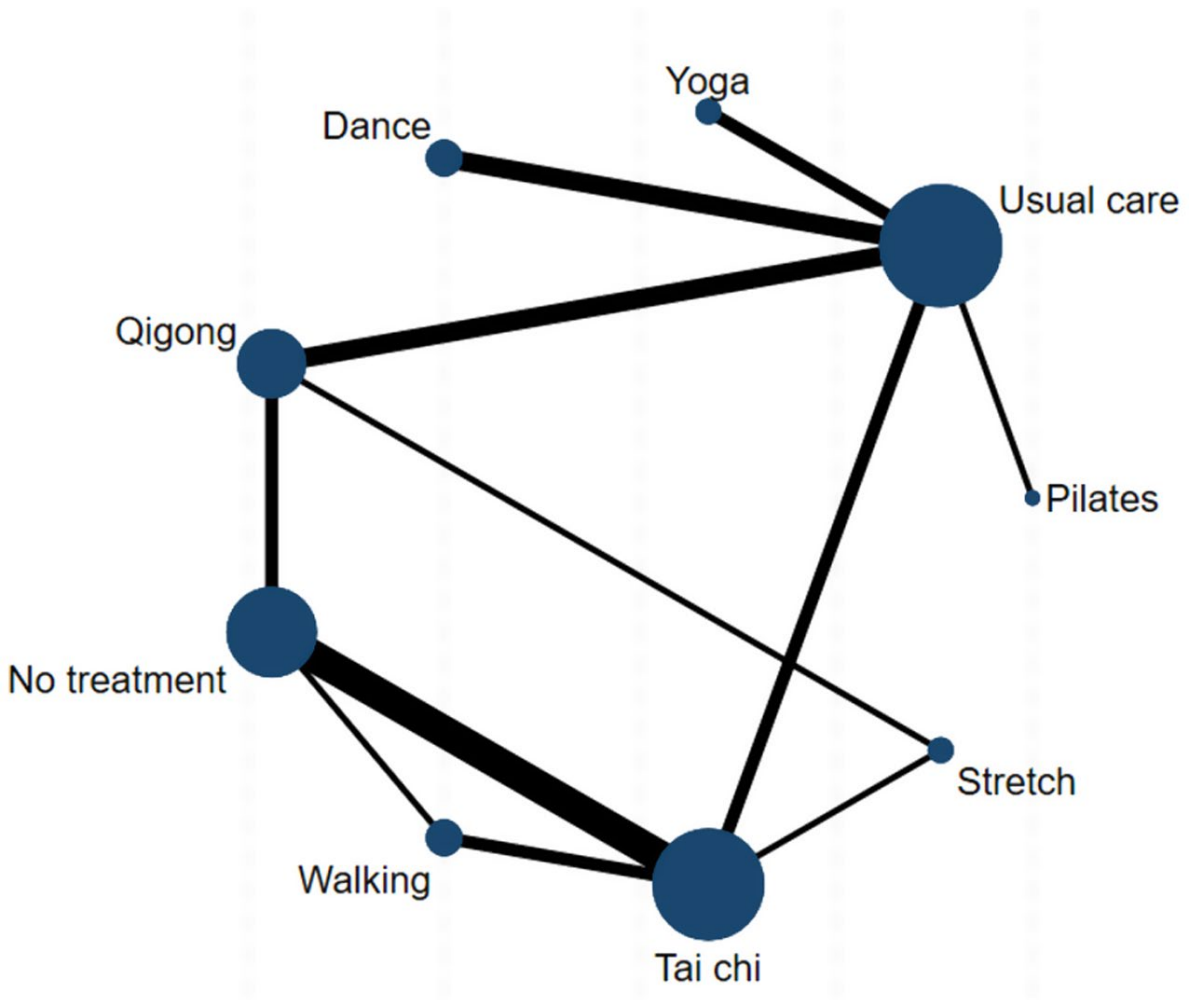
Network structure of UPDRS-III in patients with Parkinson’s disease.

The league table of pairwise comparisons (Supplementary Appendix 10.3) showed that yoga was significantly more effective than usual care [MD = −7.55; 95% CI (−13.02, −2.08)] and no treatment [MD = −9.15; 95% CI (−16.46, −1.84)]. Qigong [MD = −4.69; 95% CI (−8.98, −0.39)] and tai chi [MD = −4.44; 95% CI (−7.65, −1.24)] were significantly more effective than no treatment. No other pairwise comparisons between exercise interventions were statistically significant, as their 95% CIs crossed zero.

SUCRA values for the UPDRS-III differed across interventions (Supplementary Appendix 11.3). Pilates (SUCRA = 86.9%) and yoga (SUCRA = 84.1%) ranked highest. Dance also ranked favorably (SUCRA = 67.2%). Walking (SUCRA = 55.9%), qigong (SUCRA = 54.0%), and tai chi (SUCRA = 51.6%) showed moderate SUCRA rankings, whereas usual care (SUCRA = 21.6%) and stretching (SUCRA = 19.1%) ranked lower. No treatment had the lowest SUCRA value (9.6%). Notably, although Pilates ranked highest by SUCRA, its superiority over other interventions was not statistically significant in any pairwise comparison, with all 95% CIs crossing the null. This ranking should therefore be interpreted cautiously.

#### 3.6.4 HAMD

Seven studies involving 423 patients with Parkinson’s disease evaluated the HAMD. The comparison network included three mind–body exercise interventions (Figure 6): qigong (Baduanjin and Liuzijue; *n* = 4 studies), tai chi (*n* = 2), and yoga (*n* = 1). Usual care served as a central node, directly connected to qigong and yoga through direct evidence.

**Figure 6:**
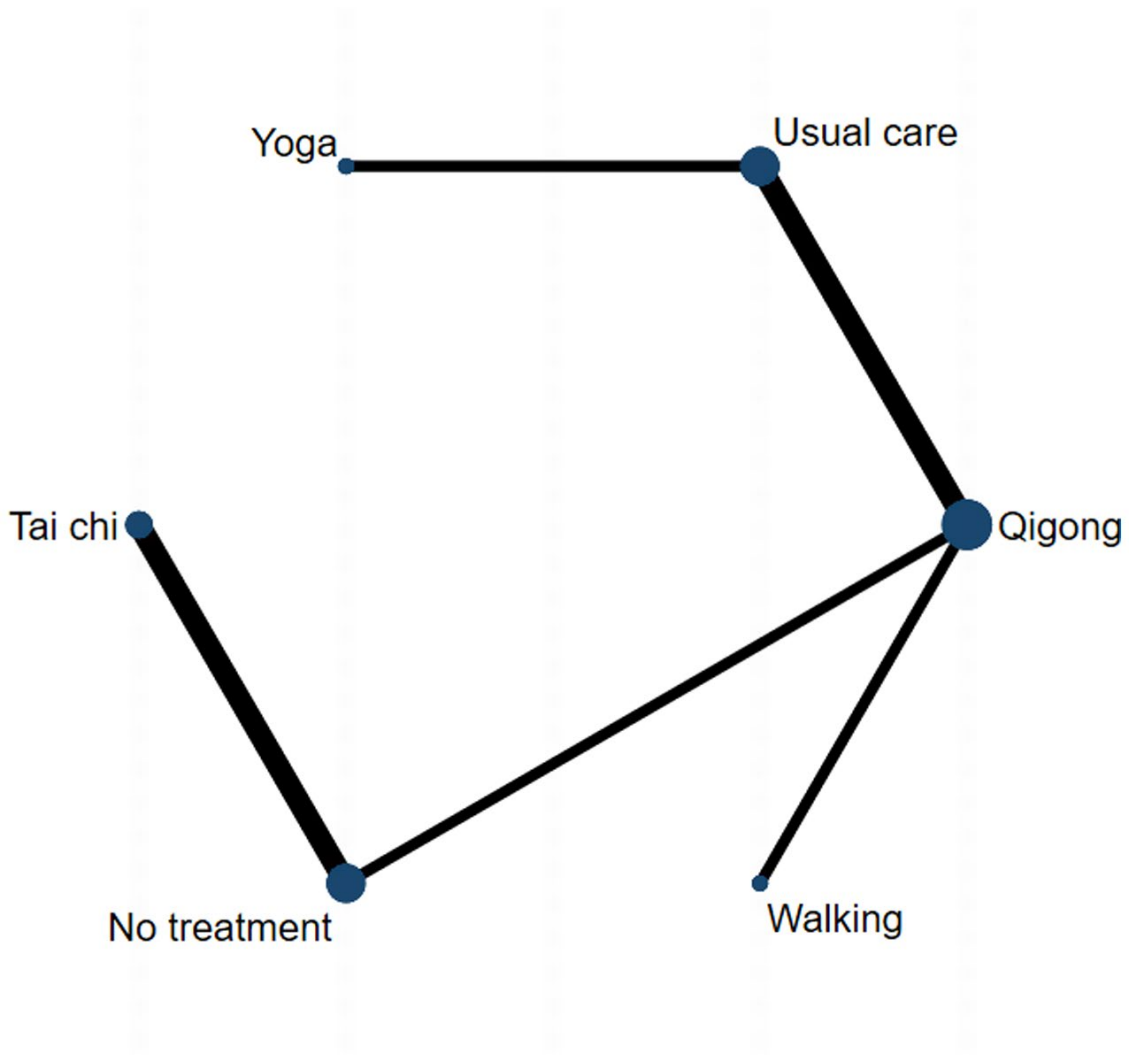
Network structure of HAMD in patients with Parkinson’s disease.

Pairwise comparisons (Supplementary Appendix 10.4) showed that tai chi was significantly more effective than qigong [MD = −6.25; 95% CI (−12.17, −0.32)], no treatment [MD = −7.03; 95% CI (−10.81, −3.24)], walking [MD = −7.23; 95% CI (−14.39, −0.06)], and usual care [MD = −8.48; 95% CI (−15.50, −1.46)]. No other pairwise comparisons between exercise interventions were statistically significant, as their 95% CIs crossed zero.

SUCRA values for the HAMD differed across interventions (Supplementary Appendix 11.4). Tai chi ranked highest (SUCRA = 97.9%). Qigong (SUCRA = 55.6%) and yoga (SUCRA = 47.6%) also ranked favorably. No treatment (SUCRA = 40.8%) and walking (SUCRA = 38.8%) ranked lower, while usual care had the lowest SUCRA value (19.3%). Notably, tai chi was connected by direct evidence only to no treatment; consequently, its comparisons with other interventions and its ranking relied substantially on indirect evidence and should be interpreted cautiously.

#### 3.6.5 PDQ-39

Seventeen studies involving 724 patients with Parkinson’s disease evaluated the PDQ-39. The comparison network included four mind–body exercise interventions (Figure 7): qigong (Baduanjin, Wuqinxi, Yijinjing, and Liuzijue; *n* = 6 studies), dance (*n* = 5), tai chi (*n* = 3), and yoga (*n* = 3). Usual care served as a central node, directly connected to tai chi, qigong, yoga, and dance through direct evidence.

**Figure 7:**
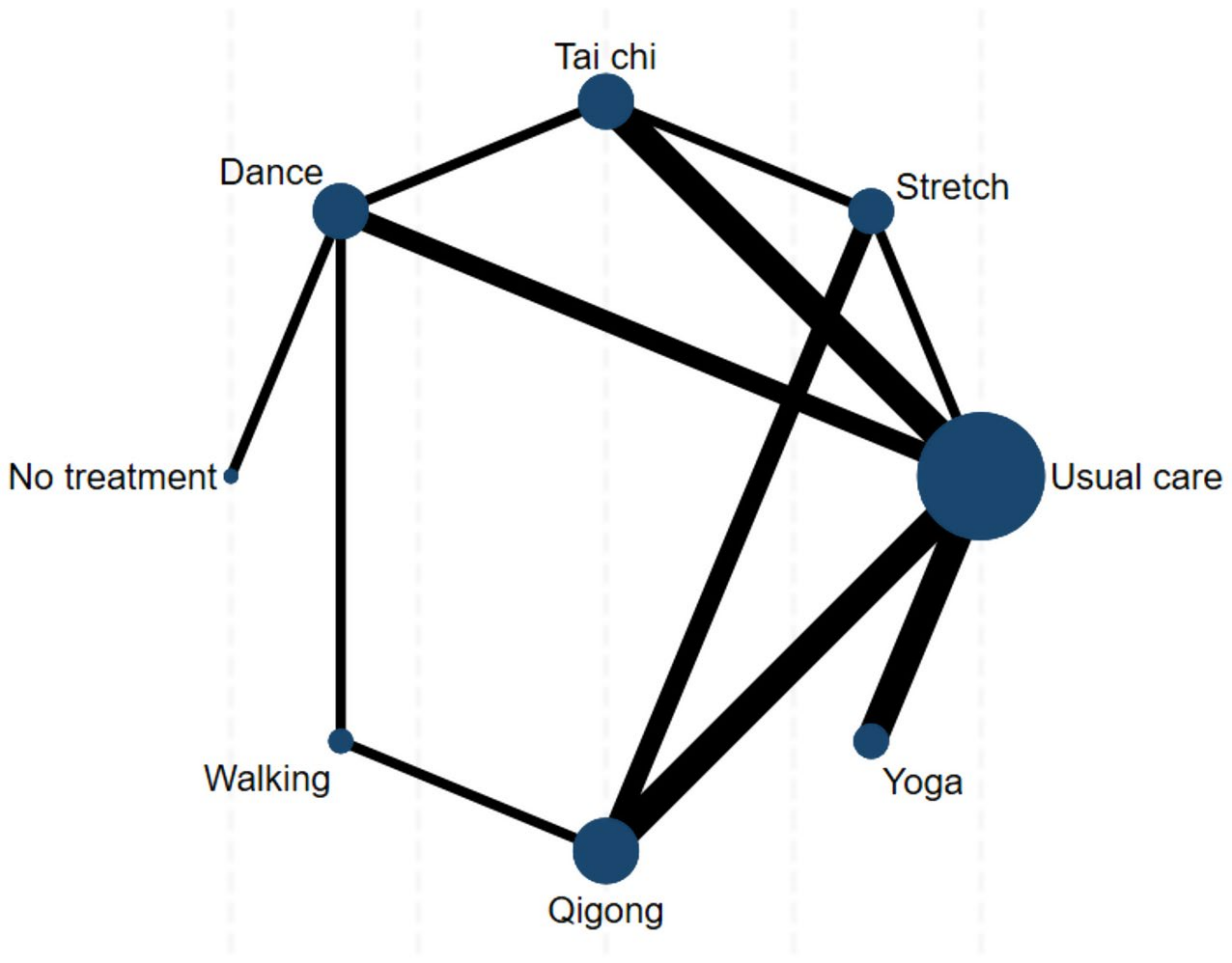
Network structure of PDQ-39 in patients with Parkinson’s disease.

Pairwise comparisons (Supplementary Appendix 10.5) showed that qigong [MD = −10.16; 95% CI (−15.32, −5.01)], walking [MD = −8.51; 95% CI (−16.83, −0.19)], and yoga [MD = −8.04; 95% CI (−15.08, −1.01)] were significantly more effective than usual care. No other pairwise comparisons between exercise interventions were statistically significant, as their 95% CIs crossed zero.

SUCRA rankings for the PDQ-39 are presented in Supplementary Appendix 11.5. Qigong ranked highest (SUCRA = 82.6%). Walking (SUCRA = 67.8%) and yoga (SUCRA = 64.6%) also ranked favorably. Tai chi (SUCRA = 49.9%) and stretching (SUCRA = 48.8%) showed moderate SUCRA rankings. Dance ranked lower (SUCRA = 31.3%), and usual care had the lowest SUCRA value (7.6%). As qigong subtypes were pooled within a single node, this ranking should not be interpreted as evidence of superiority for any specific subtype.

### 3.7 GRADE assessment

The GRADE approach for network meta-analysis was used to assess the certainty of the network estimate for each outcome and treatment comparison. For clinically relevant comparisons, the final certainty ratings ranged from moderate to very low. Downgrading was primarily attributable to study limitations (risk of bias in the contributing evidence), network imprecision, and incoherence between direct and indirect estimates for specific comparisons. The certainty of indirect comparisons was further informed by the certainty of the contributing direct evidence and the assessment of intransitivity. Detailed certainty ratings and reasons for downgrading for each comparison are provided in Supplementary Appendix 12.

### 3.8 Sensitivity analysis

Sensitivity analyses were conducted for outcomes with moderate between-study heterogeneity (UPDRS-III and PDQ-39). Under the random-effects model, the PDQ-39 ranking pattern remained unchanged, with qigong remaining the highest-ranked intervention. In contrast, the UPDRS-III rankings were sensitive to model specification: yoga became the highest-ranked intervention, whereas Pilates decreased from first to fourth place. Because this met the predefined operational criterion for a material change used in the revised analysis, an additional *post hoc* sensitivity analysis was performed by excluding the study rated as having an overall high risk of bias while retaining the fixed-effect model. After one high-risk study was excluded (Ni et al., 2016), Pilates remained the highest-ranked intervention, whereas dance moved from third to second place and yoga declined from second to third. Moreover, the significant benefit of yoga for motor function was no longer observed (Yoga vs. Usual care: MD = −4.17, 95% CI: −11.90 to 3.56; Yoga vs. No treatment: MD = −5.78, 95% CI: −14.87 to 3.31). The effects of qigong and tai chi remained significant across all sensitivity scenarios. Thus, the UPDRS-III findings for qigong and tai chi were robust, whereas the findings for yoga require more cautious interpretation. Detailed results are presented in Supplementary Appendix 13.

## 4 Discussion

This systematic review and network meta-analysis comprehensively compared the effects of five mind–body exercises on motor function, depressive symptoms, and quality of life in patients with Parkinson’s disease. Preliminary results indicated that dance ranked highest according to SUCRA for balance (BBS); qigong ranked highest for functional mobility (TUG); Pilates and yoga ranked highly for overall motor function (UPDRS-III); tai chi ranked highest for depressive symptoms (HAMD); and qigong ranked highest for health-related quality of life (PDQ-39). However, the UPDRS-III findings showed some sensitivity to analytical assumptions. Under the random-effects model, Pilates decreased from first to fourth place, whereas yoga became the highest-ranked intervention, indicating sensitivity of the rankings to model specification. In contrast, after exclusion of the high-risk study (Ni et al., 2016), while retaining the fixed-effect model, Pilates remained the highest-ranked intervention, whereas yoga declined from second to third place and its previously significant effects versus usual care and no treatment were no longer statistically significant. Thus, the Pilates ranking was sensitive to model specification but remained stable after exclusion of the high-risk study, whereas the findings for yoga should be interpreted with greater caution. The clinical interpretation of these findings should also account for the moderate to very low certainty of evidence.

Dance exercise improved BBS scores compared with usual care, walking exercise, and no treatment. Drawing on a recent estimate of the minimal important difference for the BBS in patients with Parkinson’s disease (Mehdizadeh et al., 2025), we adopted symmetric thresholds of ±2 points as an operational assumption. For dance versus usual care, both the mean difference and its entire 95% confidence interval exceeded the 2-point improvement threshold, suggesting that the benefit may be clinically meaningful as well as statistically significant. Dance is a simple, feasible, and enjoyable rehabilitation therapy (Chen et al., 2020) that naturally integrates music, cueing, movement, balance, and mobility (Sharp and Hewitt, 2014), and it has been widely applied in Parkinson’s rehabilitation. The main clinical manifestations of Parkinson’s disease include tremor, rigidity, bradykinesia, and postural instability (Beitz, 2014). Dance may activate or establish alternative motor neural pathways, thereby strengthening central control of the limbs, increasing joint flexibility, and reducing motor impairment (Huang and He, 2016). Previous studies have suggested that forward and backward stepping, turning, jumping, and single-leg support during dance training require continuous postural control, emphasizing trunk stability and potentially improving balance adaptation in patients with Parkinson’s disease (Sohn et al., 2018; Federici et al., 2005). The effect on balance may also vary by dance style. Tango is the most extensively studied dance therapy; its repeated changes in speed and rhythm place demands on foot placement and whole-body coordination (Hackney and McKee, 2014). Partnered ballroom dance involves more regular movements and fewer changes in direction and rhythm, but requires participants to respond to a partner and therefore emphasizes postural stability (Federici et al., 2005). Ballet, Irish dance, Brazilian dance, and other forms have also been used to improve balance in people with Parkinson’s disease. Importantly, dance therapy should be conducted under professional supervision and in a safe environment to minimize the risk of falls.

The TUG test assesses functional mobility, gait performance, and fall risk in patients. Qigong; reduced TUG completion time compared with usual care, Stretching, no treatment, dance, and tai chi. Drawing on recent estimates of the minimal clinically important difference for single-task TUG in patients with Parkinson’s disease (Taghizadeh et al., 2025), we adopted a 1.0-s threshold as an operational benchmark. Compared with usual care and no treatment, both the point estimates and their entire 95% confidence intervals exceeded this threshold in the direction of improvement, suggesting potentially clinically meaningful benefits. However, the confidence intervals for comparisons with dance, tai chi, and stretching included differences below this threshold, leaving the clinical importance of these comparative advantages uncertain. Qigong is a traditional Chinese physical exercise and a mind–body practice that promotes health primarily through breath regulation. Qigong practice requires practitioners to maintain a stable center of gravity, clearly differentiate between weight-bearing and non-weight-bearing limbs, and exert force symmetrically, which may enhance proprioceptive sensitivity and the integrated processing of sensory stimuli in patients with PD (Wang et al., 2019). Meanwhile, qigong adopts standing postures (Zhan Zhuang) as its fundamental training position, in which sustained knee flexion may increase lower-limb muscle strength and endurance, thereby improving balance ability (Tu and Liao, 2014). In addition, the rhythmic diaphragmatic breathing pattern of qigong may enhances the coordinated contraction of the diaphragm and transversus abdominis, elevating intra-abdominal pressure and thereby strengthening core stability (Zheng et al., 2021). Growing evidence indicates that rhythmic breathing and vocalization act as internal auditory cues, enabling patients to maintain a consistent gait rhythm and reducing both gait initiation difficulties and freezing of gait (Ghai et al., 2018). Overall, qigong training disrupts balance by introducing external forces, requiring patients to adjust their posture to restore stability. This process may improve neuromuscular coordination and dynamic balance, as evidenced by improved Timed Up and Go scores (Li et al., 2026). However, given the diversity of qigong styles, further in-depth investigation is warranted to determine which specific forms improve functional mobility in patients with PD and through what underlying mechanisms.

The UPDRS-III is a comprehensive measure of motor symptom severity and can be applied across all stages of Parkinson’s disease (Skorvanek et al., 2017). Our findings indicated that Pilates and yoga ranked relatively high according to SUCRA for improving overall motor function in patients with PD. Pilates is a strength- and flexibility-based mind–body exercise that coordinates agonist and antagonist muscle groups to effectively activate the deep spinal muscles, namely the multifidus and transversus abdominis, thereby enhancing spinal stability, core strength, and postural stability in patients with PD (Panhan et al., 2018). Long-term practice may further improve comprehensive motor attributes including balance, coordination, muscle strength, endurance, and flexibility (Kloubec, 2010). Yoga, in contrast, emphasizes control of body postures and has been shown to increase flexibility, improve balance, and enhance muscle strength (Li et al., 2017). Its effects in Parkinson’s disease may therefore resemble those of Pilates, with both approaches targeting core strength and overall motor performance. Notably, although Pilates and yoga ranked highly in the UPDRS-III analysis, the robustness of these findings remains uncertain. To contextualize clinical relevance, we adopted a 5-point threshold as an operational benchmark, informed by the minimal clinically important improvement reported for the original UPDRS motor section by Schrag et al. (2006) Pilates ranked first according to SUCRA; however, none of its pairwise comparisons reached statistical significance, with the comparison versus no treatment coming closest to significance [MD = −12.18, 95% CI (−24.97, 0.61)]. The wide confidence interval indicates substantial uncertainty around this estimate, and the ranking of Pilates was sensitive to model specification. Additionally, only one study of Pilates reported UPDRS-III outcomes, and the evidence from a single study is inherently limited. For these reasons, the available evidence is insufficient to support a robust conclusion regarding the overall effectiveness of Pilates. Similarly, the ranking and effect estimates for yoga changed in the sensitivity analyses, further suggesting limited robustness. Accordingly, the findings for both Pilates and yoga should be interpreted cautiously and require confirmation in future high-quality studies. In contrast, although tai chi and qigong ranked lower than Pilates and yoga according to SUCRA, both showed statistically significant improvements compared with no treatment, indicating measurable benefits for motor function.

The HAMD evaluates the severity of depressive symptoms and reflects emotional and depressive symptoms in patients with Parkinson’s disease. Previous evidence has consistently indicated that mind–body exercise is particularly beneficial for depressive symptoms and mood (Zhang et al., 2026). Among the included mind–body exercise interventions, tai chi had the highest SUCRA ranking for improvement in HAMD scores. Tai chi consists of slow, gentle movements that require focused attention and can be regarded as a form of dynamic meditation. Activation of the dorsolateral prefrontal cortex is closely associated with emotion regulation and cognitive control (Xu et al., 2026). Tai chi practice may enhance activation of this region, and this neurobiological effect may contribute to the attenuation of depressive symptoms and emotional distress in patients with Parkinson’s disease (Weintraub et al., 2022). However, the HAMD network was sparse, several comparisons involving tai chi relied substantially on indirect evidence, and the available evidence was limited to studies evaluating only three types of mind–body exercise. Therefore, the high SUCRA ranking of tai chi should not be interpreted as definitive evidence of superiority.

The PDQ-39 reflects multiple dimensions of quality of life in Parkinson’s disease, including mobility, activities of daily living, emotional well-being, and social support. In the present study, qigong ranked highest according to SUCRA for improving health-related quality of life and showed a statistically significant benefit compared with usual care. Similarly, we adopted thresholds of −4.72 points for improvement and +4.22 points for worsening as operational benchmarks, based on published MCID estimates for the PDQ-39 Summary Index in patients with Parkinson’s disease (Horváth et al., 2017). Qigong improved health-related quality of life compared with usual care (MD = −10.16; 95% CI, −15.32 to −5.01). Both the point estimate and the entire confidence interval exceeded the improvement threshold in magnitude, suggesting that the benefit may be clinically meaningful as well as statistically significant. We interpret the findings as follows: qigong includes multiple forms with distinct functional emphases. Yijinjing emphasizes stretching and extension and may improve flexibility (Wang et al., 2009); wuqinxi uses animal-inspired movements and may support visceral function and regulate excitation and inhibition within the nervous system (Cao et al., 2024); and liuzijue combines breathing with simple movements and may help individuals rapidly enter a calm, focused state, thereby alleviating anxiety and depression (Liang et al., 2026). Other forms include seated, walking, supine, apparatus-based, and partner qigong. Their effects may vary according to practice characteristics and exercise intensity. The pooling of diverse benefits across multiple qigong forms may partly explain its favorable effect on quality of life. Notably, walking, which was included as a comparator, ranked second according to SUCRA and showed a significant effect compared with usual care [MD = −8.51; 95% CI (−16.83, −0.19)]. Walking is simple and of moderate intensity. Although it does not specifically target balance, strength, or coordination, it may improve overall quality of life. However, the sample size for walking was limited, and walking was not a primary intervention of interest in this review; this finding should be interpreted cautiously.

## 5 Strengths and limitations

This study provides an up-to-date systematic review and network meta-analysis of the effects of mind–body exercise on multiple functional outcomes in patients with Parkinson’s disease. Rigorous eligibility criteria were applied, and the included studies and outcomes underwent standardized methodological assessment. Both global and local approaches were used to assess agreement between direct and indirect evidence, with no statistically significant inconsistency detected in the evaluable networks.

Several limitations should nevertheless be acknowledged. First, owing to the inherent nature of exercise interventions, blinding of participants and intervention providers is often infeasible, which may increase the risk of bias arising from awareness of treatment allocation. Second, differences in participant baseline characteristics and in intervention intensity, frequency, and duration contributed to heterogeneity and may limit the generalizability of the findings. We did not assess whether intervention duration or training frequency modified treatment effects, limiting conclusions about the optimal exercise dose. Third, although the distributions of the prespecified effect modifiers generally overlapped across direct comparisons, residual intransitivity cannot be excluded and may have affected the comparability of treatment comparisons and the validity of some indirect estimates. Fourth, only two randomized controlled trials evaluated Pilates, and only one reported outcomes on the Unified Parkinson’s Disease Rating Scale Part III (UPDRS-III), limiting the precision and robustness of its effect estimates and rankings. Evidence on psychological outcomes was restricted to Hamilton Depression Rating Scale (HAMD) scores from seven studies and therefore primarily reflects depressive symptoms rather than depressive symptoms more broadly. The sparse HAMD network and substantial reliance on indirect evidence for several comparisons further limit confidence in the comparative effect estimates and rankings. These limitations warrant cautious interpretation and further investigation. Fifth, qigong and dance each encompassed multiple subtypes with differing intervention characteristics. Limited subtype-specific data precluded meaningful subgroup analyses, leaving potential differences in efficacy among subtypes unresolved. Sixth, to broaden the scope of the review, walking and stretching were included as conventional physical therapy comparators. This network structure may have increased reliance on indirect evidence when comparing exercise modalities, potentially diminishing the robustness of the results. Seventh, multiple pairwise comparisons were conducted across five outcomes without adjustment for multiple testing, increasing the risk of false-positive findings. Accordingly, individual statistically significant findings should be interpreted cautiously, alongside effect sizes, confidence intervals, and certainty of evidence. Finally, most included studies lacked long-term follow-up, and evidence regarding the sustained effects of mind–body exercise remains insufficient. Future studies should therefore use large, high-quality randomized controlled designs with long-term follow-up to verify the present findings.

## 6 Conclusion

This network meta-analysis found that, compared with usual care, dance improved balance, while qigong improved functional mobility and quality of life in patients with Parkinson’s disease. Tai chi ranked highest for alleviating depressive symptoms; however, because the available evidence was based on a limited number of studies and relied substantially on indirect comparisons, considerable uncertainty remains regarding its relative advantage, and the current evidence is insufficient to establish tai chi as the most effective mind–body exercise intervention. For overall motor function, Pilates ranked highest according to SUCRA, but none of its pairwise comparisons were statistically significant. Yoga ranked second, but sensitivity analysis showed that its significant effects were not robust after a high-risk study was excluded. The UPDRS-III findings should therefore be interpreted cautiously, and the current evidence is insufficient to identify a clearly superior mind–body exercise intervention for overall motor function. Notably, these comparative findings should be interpreted in light of the moderate to very low certainty of evidence. Further high-quality randomized controlled trials are needed to clarify the comparative effects of different mind–body exercise interventions, particularly for overall motor function and depressive symptoms.

## Data Availability

The datasets presented in this study can be found in online repositories. The names of the repository/repositories and accession number(s) can be found in the article/Supplementary material.
