# Supplementary material for: Effects of mind–body exercise on motor function, quality of life, and depressive symptoms in patients with Parkinson’s disease: a systematic review and network meta-analysis
Source: Front Aging Neurosci. 2026 Oct 28;18:1982103. doi: 10.3389/fnagi.2026.1982103 (PMC13751929; doi:10.3389/fnagi.2026.1982103)
Supplement: Supplementary file 1 [file Data_Sheet_1.zip › Supplementary Material/Appendix 1-Search strategy.docx]

# **Table 1. Electronic database search strategy**

| **Database** | **Search strategies** | **Results** |
| --- | --- | --- |
| **Pubmed** | **#1** ("Parkinson Disease"[Mesh]) OR ((((((((((((((((((((((idiopathic parkinsonism[Title/Abstract]) OR (Lewy bodies of Parkinson disease[Title/Abstract])) OR (Lewy bodies of Parkinson's disease[Title/Abstract])) OR (Lewy bodies of Parkinsons disease[Title/Abstract])) OR (Lewy body Parkinson disease[Title/Abstract])) OR (Lewy body Parkinson's disease[Title/Abstract])) OR (Lewy body Parkinsons disease[Title/Abstract])) OR (paralysis agitans[Title/Abstract])) OR (Parkinson dementia complex[Title/Abstract])) OR (Parkinson's disease[Title/Abstract])) OR (Parkinsons disease[Title/Abstract])) OR (primary parkinsonism[Title/Abstract])) OR (Parkinson disease[Title/Abstract])) OR (Idiopathic Parkinson Disease[Title/Abstract])) OR (Idiopathic Parkinson's Disease[Title/Abstract])) OR (Parkinson Disease, Idiopathic[Title/Abstract])) OR (Parkinson's Disease, Idiopathic[Title/Abstract])) OR (Parkinson's Disease, Lewy Body[Title/Abstract])) OR (Parkinsonism, Primary[Title/Abstract])) OR (Parkinson Disease,Idiopathic[Title/Abstract])) OR (Parkinson's Disease, Idiopathic[Title/Abstract])) OR (Parkinson's Disease[Title/Abstract])) | **159142** |
|  | **#2** (((((((("Mind-Body Therapies"[Mesh]) OR "Meditation"[Mesh]) OR "Yoga"[Mesh]) OR "Exercise Movement Techniques"[Mesh]) OR "Tai Ji"[Mesh]) OR "Qigong"[Mesh]) OR "Mindfulness-Based Cognitive Therapy"[Mesh]) OR "Dance Therapy"[Mesh]) OR ((((((((((((((((((((((((((((((((((((((((((((((((((((((((((((((((((((((((Mind Body Therapies[Title/Abstract]) OR (Mind-Body Therapy[Title/Abstract])) OR (Therapies, Mind-Body[Title/Abstract])) OR (Therapy, Mind-Body[Title/Abstract])) OR (Mind-Body Medicine[Title/Abstract])) OR (Mind Body Medicine[Title/Abstract])) OR (alternative therapies[Title/Abstract])) OR (alternative therapy[Title/Abstract])) OR (complementary medicine[Title/Abstract])) OR (complementary therapies[Title/Abstract])) OR (eclectic medicine[Title/Abstract])) OR (eclecticism, historical[Title/Abstract])) OR (historical eclecticism[Title/Abstract])) OR (mental healing[Title/Abstract])) OR (metaphysical mind-body relations[Title/Abstract])) OR (mind body technique[Title/Abstract])) OR (mind body therapy[Title/Abstract])) OR (mind-body relations (metaphysics[Title/Abstract]))) OR (mind-body relations, metaphysical[Title/Abstract])) OR (mind-body therapies[Title/Abstract])) OR (Thomsonian medicine[Title/Abstract])) OR (alternative medicine[Title/Abstract])) OR (meditation[Title/Abstract])) OR (yogic meditation[Title/Abstract])) OR (Transcendental Meditation[Title/Abstract])) OR (Meditation, Transcendental[Title/Abstract])) OR (Exercise Movement Technics[Title/Abstract])) OR (Movement Techniques, Exercise[Title/Abstract])) OR (Pilates Based Exercises[Title/Abstract])) OR (Exercises, Pilates-Based[Title/Abstract])) OR (Training, Pilates[Title/Abstract])) OR (Pilates-Based Exercises[Title/Abstract])) OR (Pilates Training[Title/Abstract])) OR (pilates exercise[Title/Abstract])) OR (Tai-ji[Title/Abstract])) OR (Tai Chi[Title/Abstract])) OR (Chi, Tai[Title/Abstract])) OR (Tai Chi Chuan[Title/Abstract])) OR (Taiji[Title/Abstract])) OR (Taijiquan[Title/Abstract])) OR (T'ai Chi[Title/Abstract])) OR (Tai Ji Quan[Title/Abstract])) OR (Ji Quan, Tai[Title/Abstract])) OR (Quan, Tai Ji[Title/Abstract])) OR (Tai Ji[Title/Abstract])) OR (Taiji quan[Title/Abstract])) OR (Ch'i Kung[Title/Abstract])) OR (Qi Gong[Title/Abstract])) OR (Baduanjin[Title/Abstract])) OR (Yijinjing[Title/Abstract])) OR (Wuqinxi[Title/Abstract])) OR (Liuzijue[Title/Abstract])) OR (Cognitive Therapy, Mindfulness-Based[Title/Abstract])) OR (Mindfulness-Based Cognitive Therapies[Title/Abstract])) OR (Mindfulness Based Cognitive Therapy[Title/Abstract])) OR (Therapy, Mindfulness-Based Cognitive[Title/Abstract])) OR (MBCT (mindfulness-based cognitive therapy[Title/Abstract]))) OR (mindfulness cognitive therapy[Title/Abstract])) OR (mindfulness-based CBT[Title/Abstract])) OR (mindfulness-based cognitive behavior therapy[Title/Abstract])) OR (mindfulness-based cognitive behavioral therapy[Title/Abstract])) OR (mindfulness-based cognitive behaviour therapy[Title/Abstract])) OR (mindfulness-based cognitive behavioural therapy[Title/Abstract])) OR (mindfulness-CBT[Title/Abstract])) OR (mindfulness-based cognitive therapy[Title/Abstract])) OR (Therapy, Dance[Title/Abstract])) OR (Dance Therapies[Title/Abstract])) OR (Therapies, Dance[Title/Abstract])) OR (dance movement psychotherapy[Title/Abstract])) OR (dance movement therapy[Title/Abstract])) OR (dance psychotherapy[Title/Abstract])) OR (dance therapy[Title/Abstract])) | **98279** |
|  | **#3** ("Randomized Controlled Trials as Topic"[Mesh]) OR (((((((((Clinical Trials, Randomized[Title/Abstract]) OR (Trials, Randomized Clinical[Title/Abstract])) OR (Controlled Clinical Trials, Randomized[Title/Abstract])) OR (RCT[Title/Abstract])) OR (RCTS[Title/Abstract])) OR (pragmatic clinical trials as topic[Title/Abstract])) OR (randomized controlled trials[Title/Abstract])) OR (randomized controlled trials as topic[Title/Abstract])) OR (randomized controlled trial (topic[Title/Abstract]))) | **329091** |
|  | **#4** (#1 AND #2 AND #3) | **157** |
| **Web of Science** | **#1** ((((((((((((((((((((((TS=(Parkinson Disease)) OR TS=(idiopathic parkinsonism)) OR TS=(Lewy bodies of Parkinson disease)) OR TS=(Lewy bodies of Parkinson's disease)) OR TS=(Lewy bodies of Parkinsons disease)) OR TS=(Lewy body Parkinson disease)) OR TS=(Lewy body Parkinson's disease)) OR TS=(Lewy body Parkinsons disease)) OR TS=(paralysis agitans)) OR TS=(Parkinson dementia complex)) OR TS=(Parkinson's disease)) OR TS=(Parkinsons disease)) OR TS=(primary parkinsonism)) OR TS=(Parkinson disease)) OR TS=(Idiopathic Parkinson Disease)) OR TS=(Idiopathic Parkinson's Disease)) OR TS=(Parkinson Disease, Idiopathic)) OR TS=(Parkinson's Disease, Idiopathic)) OR TS=(Parkinson's Disease, Lewy Body)) OR TS=(Parkinsonism, Primary)) OR TS=(Parkinson Disease,Idiopathic)) OR TS=(Parkinson's Disease, Idiopathic)) OR TS=(Parkinson's Disease) | **279065** |
|  | **#2** ((((((((((((((((((((((((((((((((((((((((((((((((((((((((((((((((((((((((((((((((TS=(Mind-Body Therapies)) OR TS=(Meditation)) OR TS=(Yoga)) OR TS=(Exercise Movement Techniques)) OR TS=(Tai Ji)) OR TS=(Qigong)) OR TS=(Mindfulness-Based Cognitive Therapy)) OR TS=(Dance Therapy)) OR TS=(Mind Body Therapies)) OR TS=(Mind-Body Therapy)) OR TS=(Therapies, Mind-Body)) OR TS=(Therapy, Mind-Body)) OR TS=(Mind-Body Medicine)) OR TS=(Mind Body Medicine)) OR TS=(alternative therapies)) OR TS=(alternative therapy)) OR TS=(complementary medicine)) OR TS=(complementary therapies)) OR TS=(eclectic medicine)) OR TS=(eclecticism, historical)))OR TS=(historical eclecticism)) OR TS=(mental healing)) OR TS=(metaphysical mind-body relations)) OR TS=(mind body technique)) OR TS=(mind body therapy)) OR TS=(mind-body relations (metaphysics))) OR TS=(mind-body relations, metaphysical)) OR TS=(mind-body therapies)) OR TS=(Thomsonian medicine)) OR TS=(alternative medicine)) OR TS=(meditation)) OR TS=(yogic meditation)) OR TS=(Transcendental Meditation)) OR TS=(Meditation, Transcendental)) OR TS=(Exercise Movement Technics)) OR TS=(Movement Techniques, Exercise)) OR TS=(Pilates Based Exercises)) OR TS=(Exercises, Pilates-Based)) OR TS=(Training, Pilates)) OR TS=(Pilates-Based Exercises)) OR TS=(Pilates Training)) OR TS=(pilates exercise)) OR TS=(Tai-ji)) OR TS=(Tai Chi)) OR TS=(Chi, Tai)) OR TS=(Tai Chi Chuan)) OR TS=(Taiji)) OR TS=(Taijiquan)) OR TS=(T'ai Chi)) OR TS=(Tai Ji Quan)) OR TS=(Ji Quan, Tai)) OR TS=(Quan, Tai Ji)) OR TS=(Tai Ji)) OR TS=(Taiji quan)) OR TS=(Ch'i Kung)) OR TS=(Qi Gong)) OR TS=(Baduanjin)) OR TS=(Yijinjing)) OR TS=(Wuqinxi)) OR TS=(Liuzijue)) OR TS=(Cognitive Therapy, Mindfulness-Based)) OR TS=(Mindfulness-Based Cognitive Therapies)) OR TS=(Mindfulness Based Cognitive Therapy)) OR TS=(Therapy, Mindfulness-Based Cognitive)) OR TS=(MBCT (mindfulness-based cognitive therapy))) OR TS=(mindfulness cognitive therapy)) OR TS=(mindfulness-based CBT)) OR TS=(mindfulness-based cognitive behavior therapy)) OR TS=(mindfulness-based cognitive behavioral therapy)) OR TS=(mindfulness-based cognitive behaviour therapy)) OR TS=(mindfulness-based cognitive behavioural therapy)) OR TS=(mindfulness-CBT)) OR TS=(mindfulness-based cognitive therapy)) OR TS=(Therapy, Dance)) OR TS=(Dance Therapies)) OR TS=(Therapies, Dance)) OR TS=(dance movement psychotherapy)) OR TS=(dance movement therapy)) OR TS=(dance psychotherapy)) OR TS=(dance therapy) | **714518** |
|  | **#3** (((((((((TS=(Randomized Controlled Trials as Topic)) OR TS=(Clinical Trials, Randomized)) OR TS=(Trials, Randomized Clinical)) OR TS=(Controlled Clinical Trials, Randomized)) OR TS=(RCT)) OR TS=(RCTS)) OR TS=(pragmatic clinical trials as topic)) OR TS=(randomized controlled trials)) OR TS=(randomized controlled trials as topic)) OR TS=(randomized controlled trial (topic)) | **1082119** |
|  | **#4** (#1 AND #2 AND #3 ) | **853** |
| **Embase** | **#1** 'parkinson disease'/exp OR 'parkinson disease' OR 'idiopathic parkinsonism':ti,ab,kw OR 'lewy bodies of parkinson disease':ti,ab,kw OR 'lewy bodies of parkinsons disease':ti,ab,kw OR 'lewy body parkinson disease':ti,ab,kw OR 'lewy body parkinsons disease':ti,ab,kw OR 'paralysis agitans':ti,ab,kw OR 'parkinson dementia complex':ti,ab,kw OR 'primary parkinsonism':ti,ab,kw OR 'parkinson disease':ti,ab,kw OR 'idiopathic parkinson disease':ti,ab,kw OR 'idiopathic parkinsons disease':ti,ab,kw OR 'parkinson disease, idiopathic':ti,ab,kw OR 'parkinsons disease, lewy body':ti,ab,kw OR 'parkinsonism, primary':ti,ab,kw OR 'parkinson disease,idiopathic':ti,ab,kw OR 'parkinsons disease, idiopathic':ti,ab,kw OR 'parkinsons disease':ti,ab,kw | **251375** |
|  | **#2** 'alternative medicine'/exp OR 'alternative medicine' OR 'meditation'/exp OR 'meditation' OR 'yoga'/exp OR 'yoga' OR 'pilates'/exp OR 'pilates' OR 'tai chi'/exp OR 'tai chi' OR 'qigong'/exp OR 'qigong' OR 'mindfulness-based cognitive therapy'/exp OR 'mindfulness-based cognitive therapy' OR 'dance therapy'/exp OR 'dance therapy' OR 'mind body therapies':ti,ab,kw OR 'mind-body therapy':ti,ab,kw OR 'therapies, mind-body':ti,ab,kw OR 'therapy, mind-body':ti,ab,kw OR 'mind-body medicine':ti,ab,kw OR 'mind body medicine':ti,ab,kw OR 'alternative therapies':ti,ab,kw OR 'alternative therapy':ti,ab,kw OR 'complementary medicine':ti,ab,kw OR 'complementary therapies':ti,ab,kw OR 'eclectic medicine':ti,ab,kw OR 'eclecticism, historical':ti,ab,kw OR 'historical eclecticism':ti,ab,kw OR 'mental healing':ti,ab,kw OR 'metaphysical mind-body relations':ti,ab,kw OR 'mind body technique':ti,ab,kw OR 'mind body therapy':ti,ab,kw OR ('mind-body relations':ti,ab,kw AND metaphysics:ti,ab,kw) OR 'mind-body relations, metaphysical':ti,ab,kw OR 'mind-body therapies':ti,ab,kw OR 'thomsonian medicine':ti,ab,kw OR 'alternative medicine':ti,ab,kw OR meditation:ti,ab,kw OR 'yogic meditation':ti,ab,kw OR 'transcendental meditation':ti,ab,kw OR 'meditation, transcendental':ti,ab,kw OR 'exercise movement technics':ti,ab,kw OR 'movement techniques, exercise':ti,ab,kw OR 'pilates based exercises':ti,ab,kw OR 'exercises, pilates-based':ti,ab,kw OR 'training, pilates':ti,ab,kw OR 'pilates-based exercises':ti,ab,kw OR 'pilates training':ti,ab,kw OR 'pilates exercise':ti,ab,kw OR 'chi, tai':ti,ab,kw OR 'tai chi chuan':ti,ab,kw OR taiji:ti,ab,kw OR taijiquan:ti,ab,kw OR 'tai chi':ti,ab,kw OR 'tai ji quan':ti,ab,kw OR 'ji quan, tai':ti,ab,kw OR 'quan, tai ji':ti,ab,kw OR 'tai ji':ti,ab,kw OR 'taiji quan':ti,ab,kw OR 'chi kung':ti,ab,kw OR 'qi gong':ti,ab,kw OR baduanjin:ti,ab,kw OR yijinjing:ti,ab,kw OR wuqinxi:ti,ab,kw OR liuzijue:ti,ab,kw OR 'cognitive therapy, mindfulness-based':ti,ab,kw OR 'mindfulness-based cognitive therapies':ti,ab,kw OR 'mindfulness based cognitive therapy':ti,ab,kw OR 'therapy, mindfulness-based cognitive':ti,ab,kw OR (mbct:ti,ab,kw AND 'mindfulness-based cognitive therapy':ti,ab,kw) OR 'mindfulness cognitive therapy':ti,ab,kw OR 'mindfulness-based cbt':ti,ab,kw OR 'mindfulness-based cognitive behavior therapy':ti,ab,kw OR 'mindfulness-based cognitive behavioral therapy':ti,ab,kw OR 'mindfulness-based cognitive behaviour therapy':ti,ab,kw OR 'mindfulness-based cognitive behavioural therapy':ti,ab,kw OR 'mindfulness cbt':ti,ab,kw OR 'mindfulness-based cognitive therapy':ti,ab,kw OR 'therapy, dance':ti,ab,kw OR 'dance therapies':ti,ab,kw OR 'therapies, dance':ti,ab,kw OR 'dance movement psychotherapy':ti,ab,kw OR 'dance movement therapy':ti,ab,kw OR 'dance psychotherapy':ti,ab,kw OR 'dance therapy':ti,ab,kw | **176996** |
|  | **#3** 'randomized controlled trial (topic)'/exp OR 'randomized controlled trial (topic)' OR 'clinical trials, randomized':ti,ab,kw OR 'trials, randomized clinical':ti,ab,kw OR 'controlled clinical trials, randomized':ti,ab,kw OR rct:ti,ab,kw OR rcts:ti,ab,kw OR 'pragmatic clinical trials as topic':ti,ab,kw OR 'randomized controlled trials':ti,ab,kw OR 'randomized controlled trials as topic':ti,ab,kw OR ('randomized controlled trial':ti,ab,kw AND topic:ti,ab,kw) | **491596** |
|  | **#4** (#1 AND #2 AND #3 ) | **271** |
| **Cochrane Library** | **#1** MeSH descriptor: [Parkinson Disease] explode all trees | **6644** |
|  | **#2** (idiopathic parkinsonism):ti,ab,kw OR (Lewy bodies of Parkinson disease):ti,ab,kw OR (Lewy bodies of Parkinson's disease):ti,ab,kw OR (Lewy bodies of Parkinsons disease):ti,ab,kw OR (Lewy body Parkinson disease):ti,ab,kw (Word variations have been searched) OR (Lewy body Parkinson's disease):ti,ab,kw OR (Lewy body Parkinsons disease):ti,ab,kw OR (paralysis agitans):ti,ab,kw OR (Parkinson dementia complex):ti,ab,kw OR (Parkinson's disease):ti,ab,kw (Word variations have been searched) OR (Parkinsons disease):ti,ab,kw OR (primary parkinsonism):ti,ab,kw OR (Parkinson disease):ti,ab,kw OR (Idiopathic Parkinson Disease):ti,ab,kw OR (Idiopathic Parkinson's Disease):ti,ab,kw (Word variations have been searched) OR (Parkinson Disease, Idiopathic):ti,ab,kw OR (Parkinson's Disease, Idiopathic):ti,ab,kw OR (Parkinson's Disease, Lewy Body):ti,ab,kw OR (Parkinsonism, Primary):ti,ab,kw OR (Parkinson Disease,Idiopathic):ti,ab,kw (Word variations have been searched) OR (Parkinson's Disease, Idiopathic):ti,ab,kw OR (Parkinson's Disease):ti,ab,kw (Word variations have been searched) | **15510** |
|  | **#3** (#1 OR #2) | **15674** |
|  | **#4** MeSH descriptor: [Mind-Body Therapies] explode all trees | **10701** |
|  | **#5** (Mind Body Therapies):ti,ab,kw OR (Mind-Body Therapy):ti,ab,kw OR (Therapies, Mind-Body):ti,ab,kw OR (Therapy, Mind-Body):ti,ab,kw OR (Mind-Body Medicine):ti,ab,kw (Word variations have been searched) OR (Mind Body Medicine):ti,ab,kw OR (alternative therapies):ti,ab,kw OR (alternative therapy):ti,ab,kw OR (complementary medicine):ti,ab,kw OR (complementary therapies):ti,ab,kw (Word variations have been searched) OR (eclectic medicine):ti,ab,kw OR (eclecticism, historical):ti,ab,kw OR (historical eclecticism):ti,ab,kw OR (mental healing):ti,ab,kw OR (metaphysical mind-body relations):ti,ab,kw (Word variations have been searched) OR (mind body technique):ti,ab,kw OR (mind body therapy):ti,ab,kw OR (mind-body relations (metaphysics)):ti,ab,kw OR (mind-body relations, metaphysical):ti,ab,kw OR (mind-body therapies):ti,ab,kw (Word variations have been searched) OR (Thomsonian medicine):ti,ab,kw OR (alternative medicine):ti,ab,kw OR (meditation):ti,ab,kw OR (yogic meditation):ti,ab,kw OR (Transcendental Meditation):ti,ab,kw (Word variations have been searched) OR (Meditation, Transcendental):ti,ab,kw OR (Exercise Movement Technics):ti,ab,kw OR (Movement Techniques, Exercise):ti,ab,kw OR (Pilates Based Exercises):ti,ab,kw OR (Exercises, Pilates-Based):ti,ab,kw (Word variations have been searched) OR (Training, Pilates):ti,ab,kw OR (Pilates-Based Exercises):ti,ab,kw OR (Pilates Training):ti,ab,kw OR (pilates exercise):ti,ab,kw OR (Tai-ji):ti,ab,kw (Word variations have been searched) OR (Tai Chi):ti,ab,kw OR (Chi, Tai):ti,ab,kw OR (Tai Chi Chuan):ti,ab,kw OR (Taiji):ti,ab,kw OR (Taijiquan):ti,ab,kw (Word variations have been searched) OR (T'ai Chi):ti,ab,kw OR (Tai Ji Quan):ti,ab,kw OR (Ji Quan, Tai):ti,ab,kw OR (Quan, Tai Ji):ti,ab,kw OR (Tai Ji):ti,ab,kw (Word variations have been searched) OR (Taiji quan):ti,ab,kw OR (Ch'i Kung):ti,ab,kw OR (Qi Gong):ti,ab,kw OR (Baduanjin):ti,ab,kw OR (Yijinjing):ti,ab,kw (Word variations have been searched) OR (Wuqinxi):ti,ab,kw OR (Liuzijue):ti,ab,kw OR (Cognitive Therapy, Mindfulness-Based):ti,ab,kw OR (Mindfulness-Based Cognitive Therapies):ti,ab,kw OR (Mindfulness Based Cognitive Therapy):ti,ab,kw (Word variations have been searched) OR (Therapy, Mindfulness-Based Cognitive):ti,ab,kw OR (MBCT (mindfulness-based cognitive therapy)):ti,ab,kw OR (mindfulness cognitive therapy):ti,ab,kw OR (mindfulness-based CBT):ti,ab,kw OR (mindfulness-based cognitive behavior therapy):ti,ab,kw (Word variations have been searched) OR (mindfulness-based cognitive behavioral therapy):ti,ab,kw OR (mindfulness-based cognitive behaviour therapy):ti,ab,kw OR (mindfulness-based cognitive behavioural therapy):ti,ab,kw OR (mindfulness-CBT):ti,ab,kw OR (mindfulness-based cognitive therapy):ti,ab,kw (Word variations have been searched) OR(Therapy, Dance):ti,ab,kw OR (Dance Therapies):ti,ab,kw OR (Therapies, Dance):ti,ab,kw OR (dance movement psychotherapy):ti,ab,kw OR (dance movement therapy):ti,ab,kw (Word variations have been searched) OR (dance psychotherapy):ti,ab,kw OR (dance therapy):ti,ab,kw (Word variations have been searched) | **3172478** |
|  | **#6** (#4 OR #5) | **79916** |
|  | **#7** MeSH descriptor: [Randomized Controlled Trial] explode all trees | **34** |
|  | **#8** (Clinical Trials, Randomized):ti,ab,kw OR (Trials, Randomized Clinical):ti,ab,kw OR (Controlled Clinical Trials, Randomized):ti,ab,kw OR (RCT):ti,ab,kw OR (RCTS):ti,ab,kw (Word variations have been searched) OR (pragmatic clinical trials as topic):ti,ab,kw OR (randomized controlled trials):ti,ab,kw OR (randomized controlled trials as topic):ti,ab,kw OR (randomized controlled trial (topic)):ti,ab,kw (Word variations have been searched) | **1134273** |
|  | **#10** (#3 AND #6 AND #9) | **560** |
| **CNKI** | **#1** (帕金森病 + 帕金森病患者 + 帕金森病(pd) + 帕金森病病人 + 帕金森病治疗 + 帕金森病痴呆) * (身心运动 ＋冥想 + 冥想训练 + 冥想练习 + 冥想放松 + 冥想干预 + 冥想疗法＋太极拳 + 太极拳运动 + 太极拳锻炼 + 太极拳练习 + 太极拳训练 + 太极拳健身 ＋气功 + 气功疗法 + 气功锻炼 + 气功功法 + 中国气功 ＋ 八段锦 + 八段锦锻炼 + 八段锦运动 + 八段锦干预 + 八段锦训练 + 八段锦功法＋ 易筋经 + 易筋经锻炼 + 易筋经功法 + 易筋经训练 + 易筋经疗法 + 健身气功易筋经＋五禽戏 + 五禽戏锻炼 + 五禽戏运动 + 健身气功·五禽戏 + 华佗五禽戏 + 健身气功五禽戏＋ 六字诀 + 六字诀呼吸操 + 六字诀功法 + 六字诀训练 + 六字诀功法锻炼 + 六字诀呼吸吐纳功 ＋瑜伽 + 瑜伽运动 + 瑜伽练习 + 瑜伽训练 + 瑜伽锻炼 + 健身瑜伽 ＋ 普拉提 + 普拉提运动 + 普拉提训练 + 普拉提练习 + 普拉提斯运动 + 普拉提斯运动疗法 ＋ 舞蹈治疗 + 舞蹈治疗法) | **230** |
| **Wanfang Database** | **#1** (题名或关键词:("帕金森病" OR “帕金森氏病”OR “帕金森综合症“ OR ”帕金森氏综合征“ OR ”帕金森症“ OR ”帕金森氏综合征“ OR ”震颤麻痹“ OR ”帕金森综合征“ OR ”帕金森氏症“ OR "parkinson disease" OR "parkinsonian"）) AND ( 题名或关键词:(“身心运动“ OR ”身心疗法“ OR ”冥想“ OR ”太极拳“ OR ”大成拳“ OR ”意拳“ OR ”气功“ OR ”八段锦“ OR ”五禽戏“ OR ”六字诀“ OR ”易筋经“ OR ”瑜伽“ OR ”普拉提“ OR ”舞蹈“ OR "shadow boxing" OR "shadowboxing" OR "tai ji quan" OR "taijiquan"） ) | **229** |
| **VIP Database** | **#1** ((((((((((题名或关键词=帕金森病 OR 题名或关键词=帕金森氏病) OR 题名或关键词=帕金森综合症) OR 题名或关键词=帕金森氏综合征) OR 题名或关键词=帕金森症) OR 题名或关键词=帕金森氏综合征) OR 题名或关键词=震颤麻痹) OR 题名或关键词=帕金森综合征) OR 题名或关键词=帕金森氏症) OR 题名或关键词="parkinson disease") OR 题名或关键词="parkinsonian") AND (((((((((((题名或关键词=身心运动 OR 题名或关键词=身心疗法) OR 题名或关键词=冥想) OR 题名或关键词=太极拳) OR 题名或关键词=大成拳) OR 题名或关键词=意拳) OR 题名或关键词=气功) OR 题名或关键词=八段锦) OR 题名或关键词=五禽戏) OR 题名或关键词=六字诀) OR 题名或关键词=易筋经) OR 题名或关键词=瑜伽) OR 题名或关键词=普拉提) OR 题名或关键词=舞蹈) OR 题名或关键词="shadow boxing") OR 题名或关键词="shadowboxing") OR 题名或关键词="tai ji quan") OR 题名或关键词="taijiquan")) | **173** |
|  |  |  |
