# Supplementary material for: Effects of mind–body exercise on motor function, quality of life, and depressive symptoms in patients with Parkinson’s disease: a systematic review and network meta-analysis
Source: Front Aging Neurosci. 2026 Oct 28;18:1982103. doi: 10.3389/fnagi.2026.1982103 (PMC13751929; doi:10.3389/fnagi.2026.1982103)
Supplement: Supplementary file 1 [file Data_Sheet_1.zip › Supplementary Material/Appendix 11.1-BBS SUCRA.pdf]

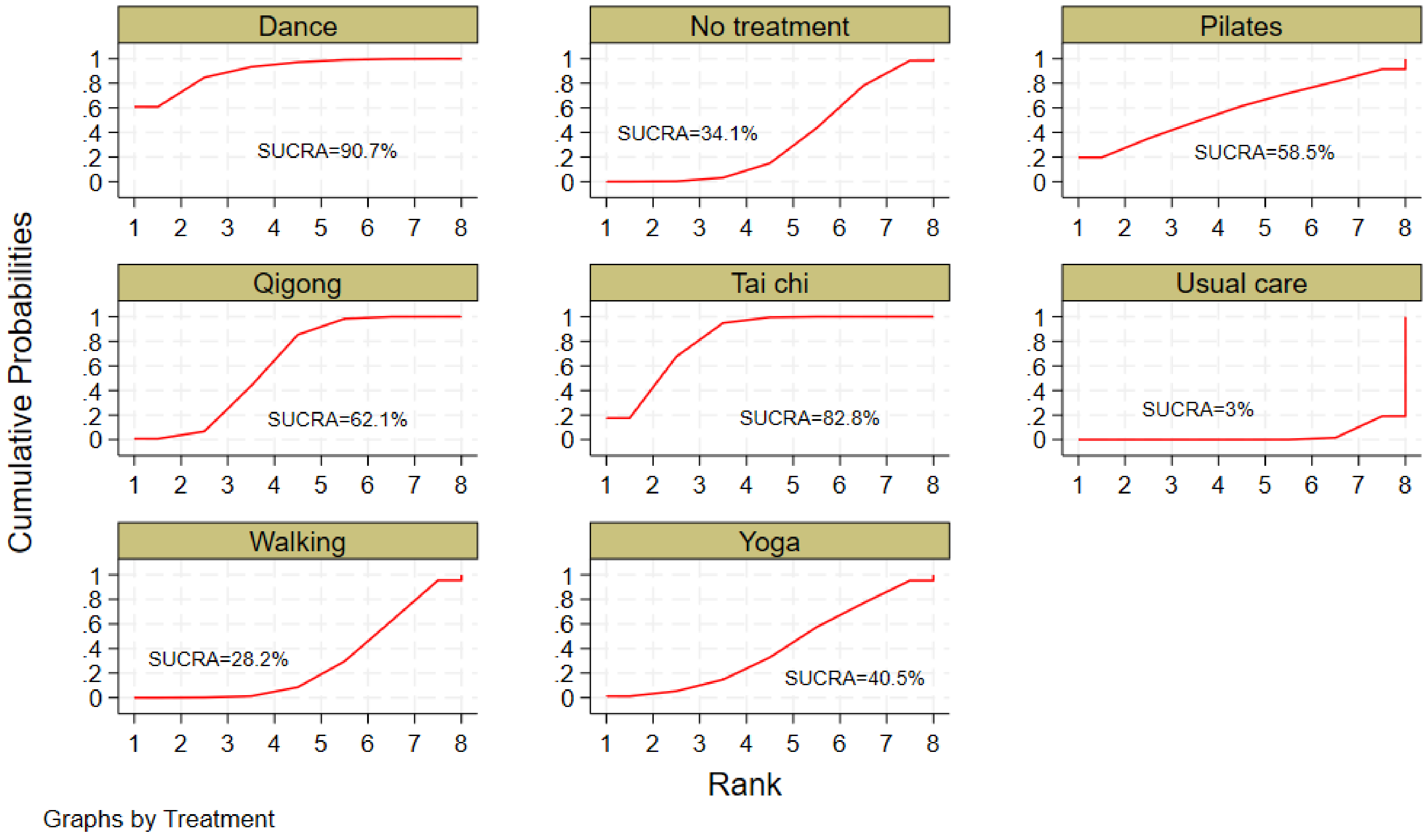
Dance
SUCRA=90.7%
No treatment
SUCRA=34.1%
Pilates
SUCRA=58.5%
Qigong
SUCRA=62.1%
Tai chi
SUCRA=82.8%
Usual care
SUCRA=3%
Walking
SUCRA=28.2%
Yoga
SUCRA=40.5%
Cumulative Probabilities
Rank
0
.2
.4
.6
.8
1
1 2 3 4 5 6 7 8
Graphs by Treatment
