# Supplementary material for: Effects of mind–body exercise on motor function, quality of life, and depressive symptoms in patients with Parkinson’s disease: a systematic review and network meta-analysis
Source: Front Aging Neurosci. 2026 Oct 28;18:1982103. doi: 10.3389/fnagi.2026.1982103 (PMC13751929; doi:10.3389/fnagi.2026.1982103)
Supplement: Supplementary file 1 [file Data_Sheet_1.zip › Supplementary Material/Appendix 11.2-TUG SUCRA.pdf]

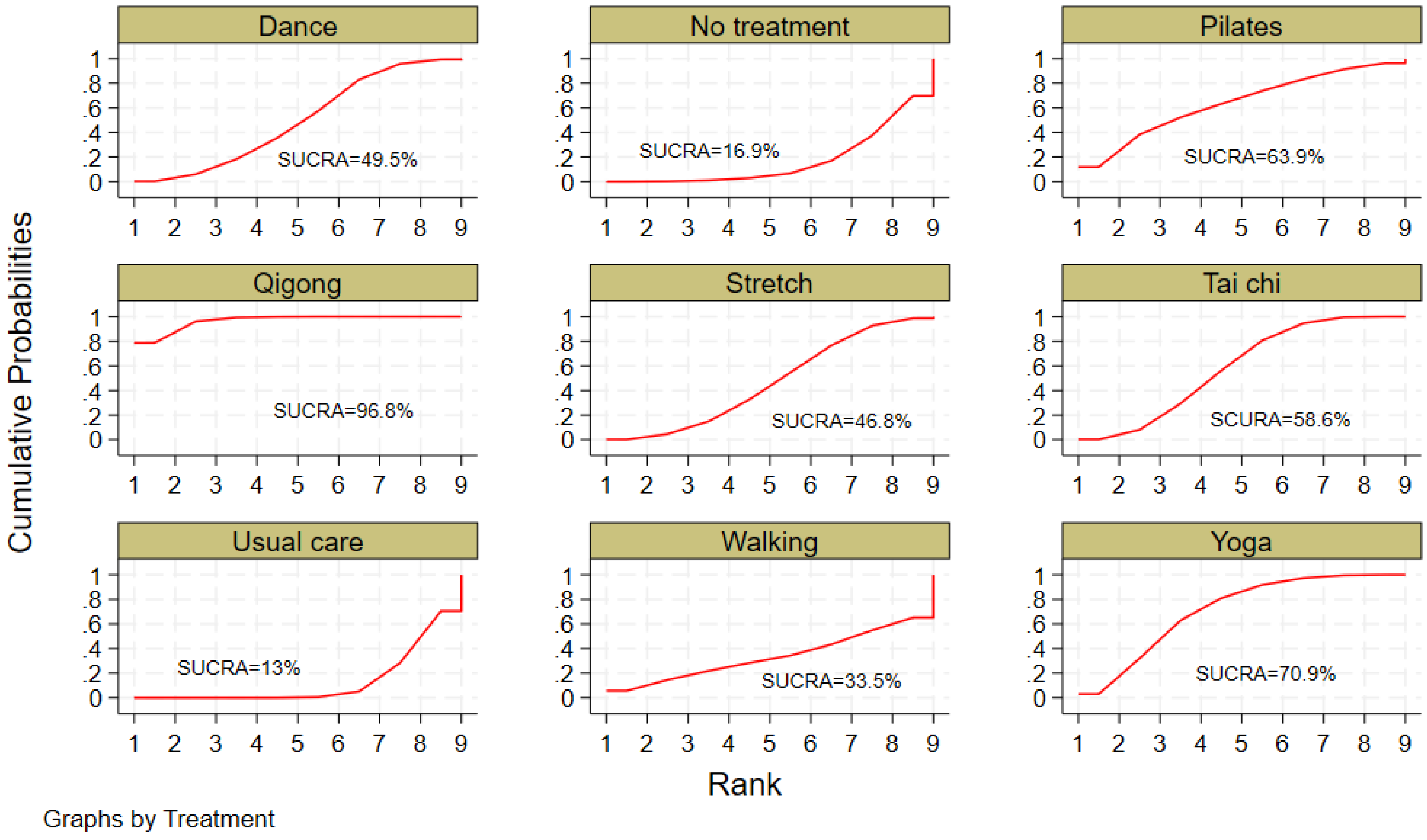
Dance
SUCRA=49.5%
No treatment
SUCRA=16.9%
Pilates
SUCRA=63.9%
Qigong
SUCRA=96.8%
Stretch
SUCRA=46.8%
Tai chi
SCURA=58.6%
Usual care
SUCRA=13%
Walking
SUCRA=33.5%
Yoga
SUCRA=70.9%
Cumulative Probabilities
Rank
Graphs by Treatment
