# Supplementary material for: Effects of mind–body exercise on motor function, quality of life, and depressive symptoms in patients with Parkinson’s disease: a systematic review and network meta-analysis
Source: Front Aging Neurosci. 2026 Oct 28;18:1982103. doi: 10.3389/fnagi.2026.1982103 (PMC13751929; doi:10.3389/fnagi.2026.1982103)
Supplement: Supplementary file 1 [file Data_Sheet_1.zip › Supplementary Material/Appendix 11.3-UPDRS-III SUCRA.pdf]

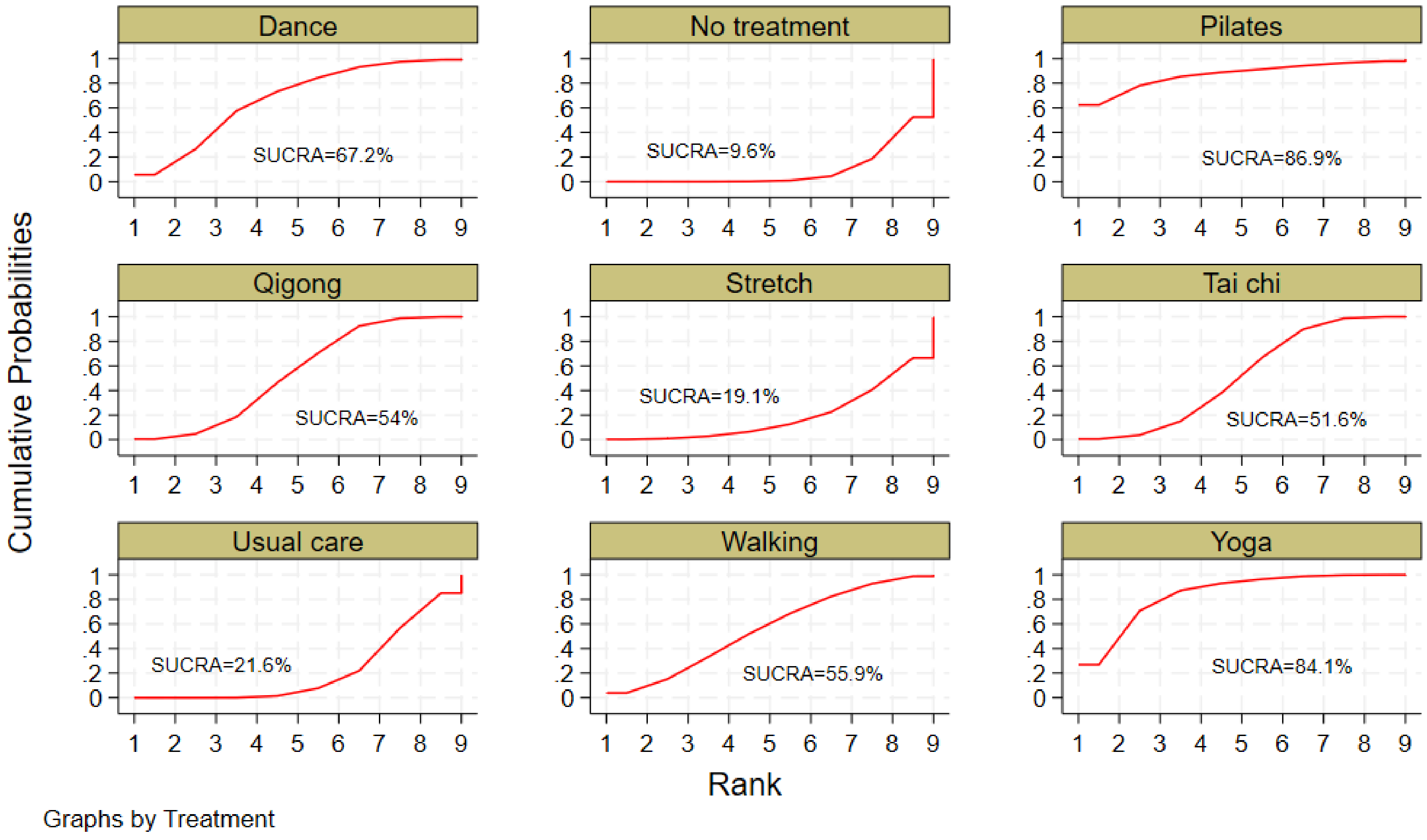

Dance
SUCRA=67.2%
No treatment
SUCRA=9.6%
Pilates
SUCRA=86.9%
Qigong
SUCRA=54%
Stretch
SUCRA=19.1%
Tai chi
SUCRA=51.6%
Usual care
SUCRA=21.6%
Walking
SUCRA=55.9%
Yoga
SUCRA=84.1%
Cumulative Probabilities
Rank
Graphs by Treatment
