# Supplementary material for: Effects of mind–body exercise on motor function, quality of life, and depressive symptoms in patients with Parkinson’s disease: a systematic review and network meta-analysis
Source: Front Aging Neurosci. 2026 Oct 28;18:1982103. doi: 10.3389/fnagi.2026.1982103 (PMC13751929; doi:10.3389/fnagi.2026.1982103)
Supplement: Supplementary file 1 [file Data_Sheet_1.zip › Supplementary Material/Appendix 11.4-HAMD SUCRA.pdf]

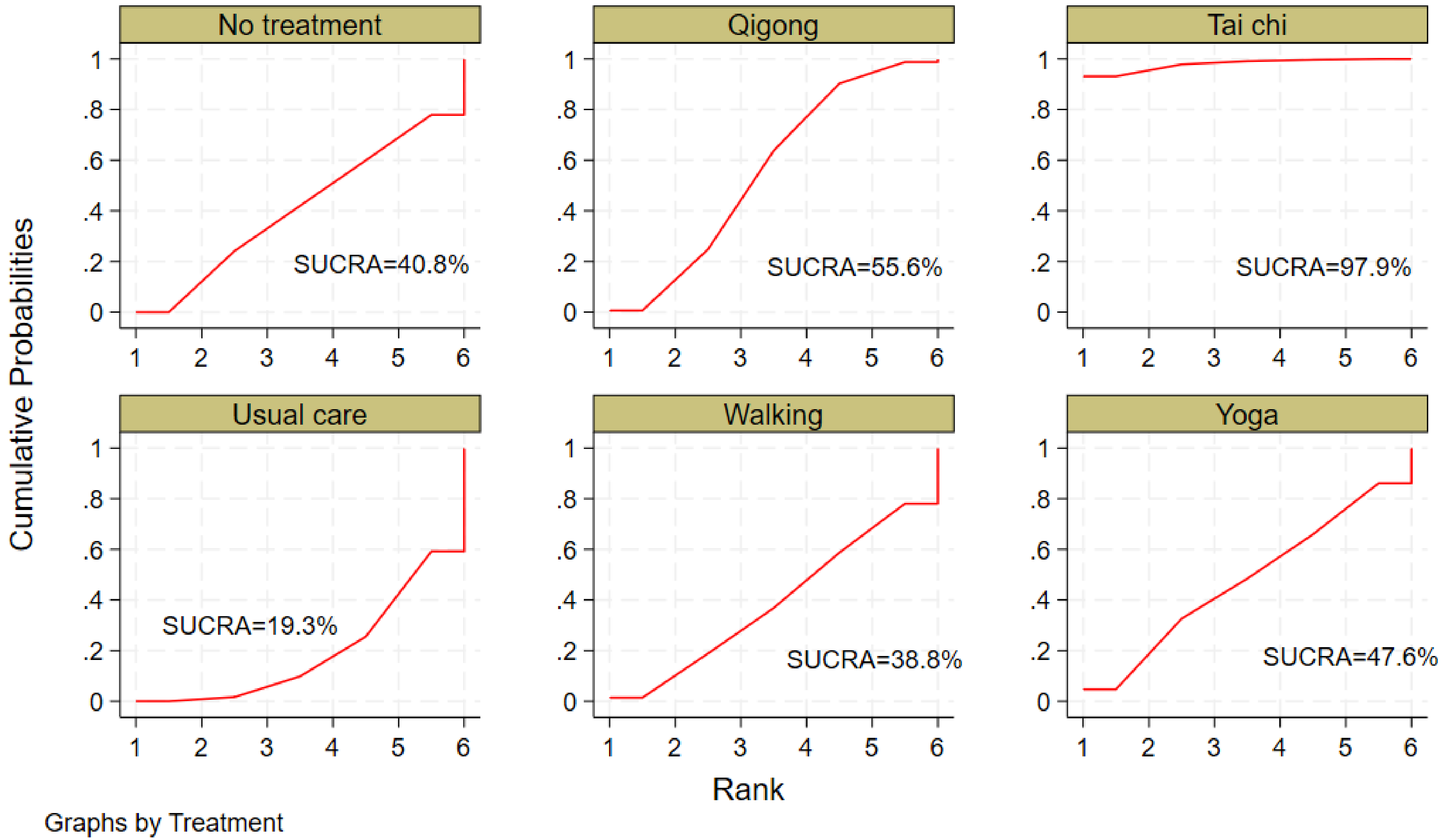
No treatment
SUCRA=40.8%
Qigong
SUCRA=55.6%
Tai chi
SUCRA=97.9%
Usual care
SUCRA=19.3%
Walking
SUCRA=38.8%
Yoga
SUCRA=47.6%
Cumulative Probabilities
Rank
Graphs by Treatment
