# Supplementary material for: Effects of mind–body exercise on motor function, quality of life, and depressive symptoms in patients with Parkinson’s disease: a systematic review and network meta-analysis
Source: Front Aging Neurosci. 2026 Oct 28;18:1982103. doi: 10.3389/fnagi.2026.1982103 (PMC13751929; doi:10.3389/fnagi.2026.1982103)
Supplement: Supplementary file 1 [file Data_Sheet_1.zip › Supplementary Material/Appendix 11.5-PDQ-39 SUCRA.pdf]

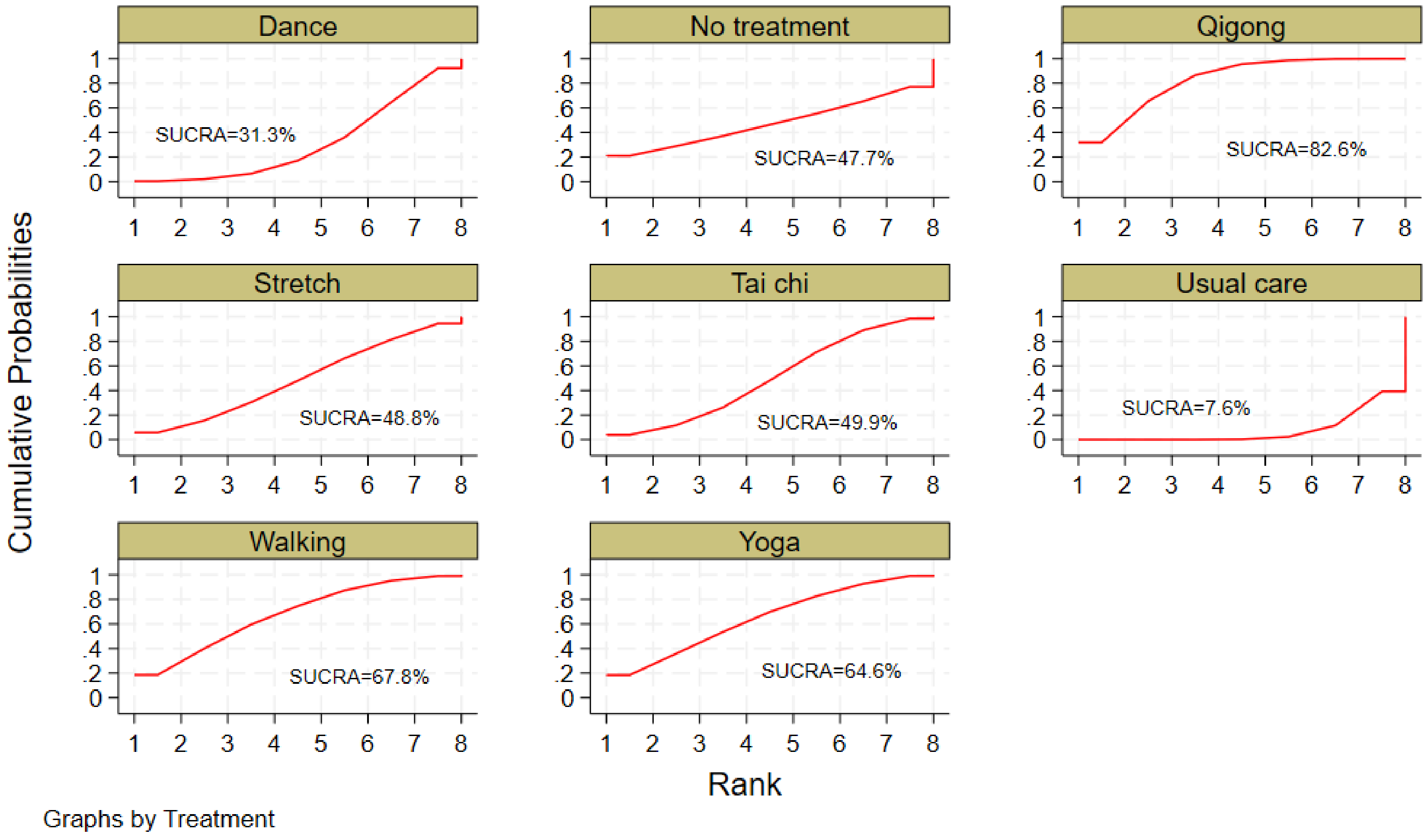

Cumulative Probabilities
Dance
SUCRA=31.3%
No treatment
SUCRA=47.7%
Qigong
SUCRA=82.6%
Stretch
SUCRA=48.8%
Tai chi
SUCRA=49.9%
Usual care
SUCRA=7.6%
Walking
SUCRA=67.8%
Yoga
SUCRA=64.6%
Rank
Graphs by Treatment
