# Supplementary material for: Effects of mind–body exercise on motor function, quality of life, and depressive symptoms in patients with Parkinson’s disease: a systematic review and network meta-analysis
Source: Front Aging Neurosci. 2026 Oct 28;18:1982103. doi: 10.3389/fnagi.2026.1982103 (PMC13751929; doi:10.3389/fnagi.2026.1982103)
Supplement: Supplementary file 1 [file Data_Sheet_1.zip › Supplementary Material/Appendix 12-GRADE.docx]

**Table 7. GRADE Summary of Findings**

**BBS-GRADE**

| **Comparison** | **Nature of the evidence** | **Direct certainty** | **Indirect certainty** | **Intransitivity** | **Incoherence** | **Network imprecision^1^** | **Final NMA certainty** | **Downgrade reason** |
| --- | --- | --- | --- | --- | --- | --- | --- | --- |
| DE vs TC | Indirect | NA | Moderate | Not serious | NA | Serious | **Low** | Risk of bias in contributing direct evidence;  network imprecision |
| DE vs QG | Indirect | NA | Moderate | Not serious | NA | Serious | **Low** | Risk of bias in contributing direct evidence;  network imprecision |
| DE vs PE | Indirect | NA | Moderate | Not serious | NA | Very serious | **Very low** | Risk of bias in contributing direct evidence;  very serious network imprecision |
| DE vs YG | Indirect | NA | Low | Not serious | NA | Serious | **Very low** | Very serious risk of bias in contributing direct evidence;  network imprecision |
| DE vs NT | Indirect | NA | Moderate | Not serious | NA | Serious | **Low** | Risk of bias in contributing direct evidence;  network imprecision |
| DE vs WE | Indirect | NA | Moderate | Not serious | NA | Serious | **Low** | Risk of bias in contributing direct evidence;  network imprecision |
| DE vs UC | Mixed,  direct-dominant | Moderate | Not informative | NA | Not assessable | Not serious | **Moderate** | Risk of bias |
| TC vs QG | Indirect | NA | Moderate | Not serious | NA | Serious | **Low** | Risk of bias in contributing direct evidence;  network imprecision |
| TC vs PE | Indirect | NA | Moderate | Not serious | NA | Very serious | **Very low** | Risk of bias in contributing direct evidence;  very serious network imprecision |
| TC vs YG | Indirect | NA | Low | Not serious | NA | Serious | **Very low** | Very serious risk of bias in contributing direct evidence; network imprecision |
| TC vs NT | Mixed | Moderate | Moderate | Not serious | Not serious | Serious | **Low** | Risk of bias;  network imprecision |
| TC vs WE | Mixed | Moderate | Moderate | Not serious | Not serious | Serious | **Low** | Risk of bias;  network imprecision |
| TC vs UC | Mixed | Moderate | Moderate | Not serious | Not serious | Not serious | **Moderate** | Risk of bias |
| QG vs PE | Indirect | NA | Moderate | Not serious | NA | Very serious | **Very low** | Risk of bias in contributing direct evidence;  very serious network imprecision |
| QG vs YG | Indirect | NA | Low | Not serious | NA | Serious | **Very low** | Very serious risk of bias in contributing direct evidence; network imprecision |
| QG vs NT | Mixed | Moderate | Moderate | Not serious | Not serious | Serious | **Low** | Risk of bias;  network imprecision |
| QG vs WE | Mixed | **High** | Moderate | Not serious | Not serious | Serious | **Moderate** | Network imprecision |
| QG vs UC | Mixed | Moderate | Moderate | Not serious | Not serious | Not serious | **Moderate** | Risk of bias |
| PE vs YG | Indirect | NA | Low | Not serious | NA | Very serious | **Very low** | Very serious risk of bias in contributing direct evidence;  very serious network imprecision |
| PE vs NT | Indirect | NA | Moderate | Not serious | NA | Very serious | **Very low** | Risk of bias in contributing direct evidence;  very serious network imprecision |
| PE vs WE | Indirect | NA | Moderate | Not serious | NA | Very serious | **Very low** | Risk of bias in contributing direct evidence;  very serious network imprecision |
| PE vs UC | Mixed,  direct-dominant | Moderate | Not informative | NA | Not assessable | Serious | **Low** | Risk of bias;  network imprecision |
| YG vs NT | Indirect | NA | Low | Not serious | NA | Very serious | **Very low** | Very serious risk of bias in contributing direct evidence;  very serious network imprecision |
| YG vs WE | Indirect | NA | Low | Not serious | NA | Very serious | **Very low** | Very serious risk of bias in contributing direct evidence;  very serious network imprecision |
| YG vs UC | Mixed,  direct-dominant | Low | Not informative | NA | Not assessable | Serious | **Very low** | Very serious risk of bias;  network imprecision |
| NT vs WE | Indirect | NA | Moderate | Not serious | NA | Serious | **Low** | Risk of bias in contributing direct evidence;  network imprecision |
| NT vs UC | Indirect | NA | Moderate | Not serious | NA | Serious | **Low** | Risk of bias in contributing direct evidence;  network imprecision |
| WE vs UC | Indirect | NA | Moderate | Not serious | NA | Serious | **Low** | Risk of bias in contributing direct evidence;  network imprecision |

1.BBS: Network imprecision was assessed using a 2-point threshold, obtained by rounding the improvement-rate-adjusted MID of 1.97 points reported in a Parkinson’s disease cohort by Mehdizadeh et al^[1]^. The use of symmetric thresholds of ±2 points was an operational assumption of this review; the cited study did not establish an equivalent 2-point threshold for worsening.

**TUG-GRADE**

| **Comparison** | **Nature of the evidence** | **Direct certainty** | **Indirect certainty** | **Intransitivity** | **Incoherence** | **Network imprecision^2^** | **Final NMA certainty** | **Downgrade reason** |
| --- | --- | --- | --- | --- | --- | --- | --- | --- |
| **QG vs YG** | Indirect | NA | Low | Not serious | NA | Serious | **Very low** | Very serious risk of bias in contributing direct evidence; network imprecision |
| **QG vs PE** | Indirect | NA | Moderate | Not serious | NA | Serious | **Low** | Risk of bias in contributing direct evidence;  network imprecision |
| **QG vs TC** | Indirect | NA | Moderate | Not serious | NA | Serious | **Low** | Risk of bias in contributing direct evidence;  network imprecision |
| **QG vs DE** | Indirect | NA | Moderate | Not serious | NA | Serious | **Low** | Risk of bias in contributing direct evidence;  network imprecision |
| **QG vs SE** | Mixed | High | Moderate | Not serious | Not serious | Serious | **Low** | Risk of bias in contributing indirect evidence;  network imprecision |
| **QG vs WE** | Indirect | NA | Moderate | Not serious | NA | Serious | **Low** | Risk of bias in contributing direct evidence;  network imprecision |
| **QG vs NT** | Mixed | Moderate | Moderate | Not serious | Not serious | Not serious | **Moderate** | Risk of bias |
| **QG vs UC** | Mixed | Moderate | High | Not serious | Not serious | Not serious | **Moderate** | Risk of bias |
| **YG vs PE** | Indirect | NA | Low | Not serious | NA | Very serious | **Very low** | Very serious risk of bias in contributing direct evidence;  very serious network imprecision |
| **YG vs TC** | Mixed | Moderate | Low | Not serious | Not serious | Serious | **Very low** | Very serious risk of bias in contributing indirect evidence; network imprecision |
| **YG vs DE** | Indirect | NA | Low | Not serious | NA | Serious | **Very low** | Very serious risk of bias in contributing direct evidence; network imprecision |
| **YG vs SE** | Mixed | Moderate | Low | Not serious | Not serious | Serious | **Very low** | Very serious risk of bias in contributing indirect evidence; network imprecision |
| **YG vs WE** | Indirect | NA | Low | Not serious | NA | Very serious | **Very low** | Very serious risk of bias in contributing direct evidence;  very serious network imprecision |
| **YG vs NT** | Indirect | NA | Moderate | Not serious | NA | Serious | **Low** | Risk of bias in contributing direct evidence;  network imprecision |
| **YG vs UC** | Mixed | Low | Moderate | Not serious | Not serious | Serious | **Very low** | Very serious risk of bias;  network imprecision |
| **PE vs TC** | Indirect | NA | Moderate | Not serious | NA | Very serious | **Very low** | Risk of bias in contributing direct evidence;  very serious network imprecision |
| **PE vs DE** | Indirect | NA | Moderate | Not serious | NA | Very serious | **Very low** | Risk of bias in contributing direct evidence;  very serious network imprecision |
| **PE vs SE** | Indirect | NA | Moderate | Not serious | NA | Very serious | **Very low** | Risk of bias in contributing direct evidence;  very serious network imprecision |
| **PE vs WE** | Indirect | NA | Moderate | Not serious | NA | Very serious | **Very low** | Risk of bias in contributing direct evidence;  very serious network imprecision |
| **PE vs NT** | Indirect | NA | Moderate | Not serious | NA | Serious | **Low** | Risk of bias in contributing direct evidence;  network imprecision |
| **PE vs UC** | Mixed, direct-dominant | Moderate | Not informative | NA | Not assessable | Serious | **Low** | Risk of bias;  network imprecision |
| **TC vs DE** | Indirect | NA | Moderate | Not serious | NA | Very serious | **Very low** | Risk of bias in contributing direct evidence;  very serious network imprecision |
| **TC vs SE** | Mixed | High | High | Not serious | Not serious | Serious | **Moderate** | Network imprecision |
| **TC vs WE** | Indirect | NA | Moderate | Not serious | NA | Very serious | **Very low** | Risk of bias in contributing direct evidence;  very serious network imprecision |
| **TC vs NT** | Mixed | Moderate | Moderate | Not serious | Not serious | Serious | **Low** | Risk of bias;  network imprecision |
| **TC vs UC** | Mixed | High | Low | Not serious | Not serious | Serious | **Moderate** | Network imprecision |
| **DE vs SE** | Indirect | NA | Moderate | Not serious | NA | Very serious | **Very low** | Risk of bias in contributing direct evidence;  very serious network imprecision |
| **DE vs WE** | Mixed, direct-dominant | Moderate | Not informative | NA | Not assessable | Very serious | **Very low** | Risk of bias;  very serious network imprecision |
| **DE vs NT** | Mixed | Moderate | Moderate | Not serious | Not serious | Serious | **Low** | Risk of bias;  network imprecision |
| **DE vs UC** | Mixed | Moderate | Moderate | Not serious | Not serious | Serious | **Low** | Risk of bias;  network imprecision |
| **SE vs WE** | Indirect | NA | Moderate | Not serious | NA | Very serious | **Very low** | Risk of bias in contributing direct evidence;  very serious network imprecision |
| **SE vs NT** | Indirect | NA | Moderate | Not serious | NA | Serious | **Low** | Risk of bias in contributing direct evidence;  network imprecision |
| **SE vs UC** | Mixed | High | High | Not serious | Not serious | Serious | **Moderate** | Network imprecision |
| **WE vs NT** | Indirect | NA | Moderate | Not serious | NA | Very serious | **Very low** | Risk of bias in contributing direct evidence;  very serious network imprecision |
| **WE vs UC** | Indirect | NA | Moderate | Not serious | NA | Very serious | **Very low** | Risk of bias in contributing direct evidence;  very serious network imprecision |
| **NT vs UC** | Indirect | NA | Moderate | Not serious | NA | Very serious | **Very low** | Risk of bias in contributing direct evidence;  very serious network imprecision |

2.TUG: Network imprecision was assessed using an operational threshold of 1.0 second. This value was informed by the single-task TUG MCID estimates reported by Taghizadeh et al. in Parkinson’s disease: 0.71–1.27 seconds using anchor-based methods and 1.07–1.14 seconds using distribution-based methods ^[2]^. The selected 1.0-second threshold is a pragmatic benchmark rather than a universally validated cut-off.

**UPDRS-Ⅲ-GRADE**

| **Comparison** | **Nature of the evidence** | **Direct certainty** | **Indirect certainty** | **Intransitivity** | **Incoherence** | **Network imprecision^3^** | **Final NMA certainty** | **Downgrade reason** |
| --- | --- | --- | --- | --- | --- | --- | --- | --- |
| **PE vs YG** | Indirect | NA | Low | Not serious | NA | Very serious | **Very low** | Very serious risk of bias in contributing direct evidence;  very serious network imprecision |
| **PE vs DE** | Indirect | NA | Moderate | Not serious | NA | Very serious | **Very low** | Risk of bias in contributing direct evidence;  very serious network imprecision |
| **PE vs WE** | Indirect | NA | High | Not serious | NA | Very serious | **Low** | Very serious network imprecision |
| **PE vs QG** | Indirect | NA | Moderate | Not serious | NA | Serious | **Low** | Risk of bias in contributing direct evidence;  network imprecision |
| **PE vs TC** | Indirect | NA | High | Not serious | NA | Serious | **Moderate** | Network imprecision |
| **PE vs UC** | Mixed, direct-dominant | High | Not informative | NA | Not assessable | Serious | **Moderate** | Network imprecision |
| **PE vs SE** | Indirect | NA | High | Not serious | NA | Serious | **Moderate** | Network imprecision |
| **PE vs NT** | Indirect | NA | Moderate | Not serious | NA | Serious | **Low** | Risk of bias in contributing direct evidence;  network imprecision |
| **YG vs DE** | Indirect | NA | Low | Not serious | NA | Serious | **Very low** | Very serious risk of bias in contributing direct evidence; network imprecision |
| **YG vs WE** | Indirect | NA | Low | Not serious | NA | Serious | **Very low** | Very serious risk of bias in contributing direct evidence; network imprecision |
| **YG vs QG** | Indirect | NA | Low | Not serious | NA | Serious | **Very low** | Very serious risk of bias in contributing direct evidence; network imprecision |
| **YG vs TC** | Indirect | NA | Low | Not serious | NA | Serious | **Very low** | Very serious risk of bias in contributing direct evidence; network imprecision |
| **YG vs UC** | Mixed, direct-dominant | Low | Not informative | NA | Not assessable | Serious | **Very low** | Very serious risk of bias;  network imprecision |
| **YG vs SE** | Indirect | NA | Low | Not serious | NA | Serious | **Very low** | Very serious risk of bias in contributing direct evidence; network imprecision |
| **YG vs NT** | Indirect | NA | Low | Not serious | NA | Serious | **Very low** | Very serious risk of bias in contributing direct evidence; network imprecision |
| **DE vs WE** | Indirect | NA | Moderate | Not serious | NA | Very serious | **Very low** | Risk of bias in contributing direct evidence;  very serious network imprecision |
| **DE vs QG** | Indirect | NA | Moderate | Not serious | NA | Serious | **Low** | Risk of bias in contributing direct evidence;  network imprecision |
| **DE vs TC** | Indirect | NA | Moderate | Not serious | NA | Serious | **Low** | Risk of bias in contributing direct evidence;  network imprecision |
| **DE vs UC** | Mixed, direct-dominant | Moderate | Not informative | NA | Not assessable | Serious | **Low** | Risk of bias;  network imprecision |
| **DE vs SE** | Indirect | NA | Moderate | Not serious | NA | Serious | **Low** | Risk of bias in contributing direct evidence;  network imprecision |
| **DE vs NT** | Indirect | NA | Moderate | Not serious | NA | Serious | **Low** | Risk of bias in contributing direct evidence;  network imprecision |
| **WE vs QG** | Indirect | NA | Moderate | Not serious | NA | Very serious | **Very low** | Risk of bias in contributing direct evidence;  very serious network imprecision |
| **WE vs TC** | Mixed | High | Moderate | Not serious | Not serious | Serious | **Low** | Risk of bias in contributing indirect evidence;  network imprecision |
| **WE vs UC** | Indirect | NA | High | Not serious | NA | Serious | **Moderate** | Network imprecision |
| **WE vs SE** | Indirect | NA | High | Not serious | NA | Serious | **Moderate** | Network imprecision |
| **WE vs NT** | Mixed, direct-dominant | High | Moderate | Not serious | Not serious | Serious | **Moderate** | Network imprecision |
| **QG vs TC** | Indirect | NA | Moderate | Not serious | NA | Not serious | **Moderate** | Risk of bias in contributing direct evidence |
| **QG vs UC** | Mixed, direct-dominant | Moderate | High | Not serious | Not serious | Serious | **Low** | Risk of bias;  network imprecision |
| **QG vs SE** | Mixed | High | Moderate | Not serious | Not serious | Serious | **Low** | Risk of bias in contributing indirect evidence;  network imprecision |
| **QG vs NT** | Mixed | Moderate | Moderate | Not serious | **Serious** | Serious | **Very low** | Risk of bias; incoherence;  network imprecision |
| **TC vs UC** | Mixed | High | Moderate | Not serious | Not serious | Serious | **Low** | Risk of bias in contributing indirect evidence;  network imprecision |
| **TC vs SE** | Mixed, direct-dominant | High | Moderate | Not serious | Not serious | Serious | **Moderate** | Network imprecision |
| **TC vs NT** | Mixed | Moderate | High | Not serious | **Serious** | Serious | **Low** | Incoherence;  network imprecision |
| **UC vs SE** | Indirect | NA | High | Not serious | NA | Very serious | **Low** | Very serious network imprecision |
| **UC vs NT** | Indirect | NA | Moderate | Not serious | NA | Serious | **Low** | Risk of bias in contributing direct evidence;  network imprecision |
| **SE vs NT** | Indirect | NA | Moderate | Not serious | NA | Very serious | **Very low** | Risk of bias in contributing direct evidence;  very serious network imprecision |

3.UPDRS-III: Network imprecision was assessed using a 5-point threshold, informed by Schrag et al., who identified a 5-point improvement on the original UPDRS motor section as a clinically important change in patients with early Parkinson’s disease ^[3]^. This estimate was not derived for the MDS-UPDRS-III.

**PDQ-39-GRADE**

| **Comparison** | **Nature of the evidence** | **Direct certainty** | **Indirect certainty** | **Intransitivity** | **Incoherence** | **Network imprecision^4^** | **Final NMA certainty** | **Downgrade reason** |
| --- | --- | --- | --- | --- | --- | --- | --- | --- |
| **QG vs WE** | Mixed, direct-dominant | High | Moderate | Not serious | Not serious | Very serious | **Low** | Very serious network imprecision |
| **QG vs YG** | Indirect | NA | Moderate | Not serious | NA | Very serious | **Very low** | Risk of bias in contributing direct evidence;  very serious network imprecision |
| **QG vs TC** | Indirect | NA | Moderate | Not serious | NA | Serious | **Low** | Risk of bias in contributing direct evidence;  network imprecision |
| **QG vs SE** | Mixed | High | Moderate | Not serious | Not serious | Serious | **Low** | Risk of bias in contributing indirect evidence;  network imprecision |
| **QG vs NT** | Indirect | NA | Moderate | Not serious | NA | Very serious | **Very low** | Risk of bias in contributing direct evidence;  very serious network imprecision |
| **QG vs DE** | Indirect | NA | Moderate | Not serious | NA | Serious | **Low** | Risk of bias in contributing direct evidence;  network imprecision |
| **QG vs UC** | Mixed | Moderate | High | Not serious | Not serious | Not serious | **Moderate** | Risk of bias in contributing direct evidence |
| **WE vs YG** | Indirect | NA | Moderate | Not serious | NA | Very serious | **Very low** | Risk of bias in contributing direct evidence;  very serious network imprecision |
| **WE vs TC** | Indirect | NA | Moderate | Not serious | NA | Very serious | **Very low** | Risk of bias in contributing direct evidence;  very serious network imprecision |
| **WE vs SE** | Indirect | NA | High | Not serious | NA | Very serious | **Low** | Very serious network imprecision |
| **WE vs NT** | Indirect | NA | Moderate | Not serious | NA | Very serious | **Very low** | Risk of bias in contributing direct evidence;  very serious network imprecision |
| **WE vs DE** | Mixed | Moderate | Moderate | Not serious | Not serious | Serious | **Low** | Risk of bias;  network imprecision |
| **WE vs UC** | Indirect | NA | Moderate | Not serious | NA | Serious | **Low** | Risk of bias in contributing direct evidence;  network imprecision |
| **YG vs TC** | Indirect | NA | Moderate | Not serious | NA | Very serious | **Very low** | Risk of bias in contributing direct evidence;  very serious network imprecision |
| **YG vs SE** | Indirect | NA | Moderate | Not serious | NA | Very serious | **Very low** | Risk of bias in contributing direct evidence;  very serious network imprecision |
| **YG vs NT** | Indirect | NA | Moderate | Not serious | NA | Very serious | **Very low** | Risk of bias in contributing direct evidence;  very serious network imprecision |
| **YG vs DE** | Indirect | NA | Moderate | Not serious | NA | Very serious | **Very low** | Risk of bias in contributing direct evidence;  very serious network imprecision |
| **YG vs UC** | Mixed, direct-dominant | Moderate | Not informative | NA | Not assessable | Serious | **Low** | Risk of bias;  network imprecision |
| **TC vs SE** | Mixed | High | Moderate | Not serious | Serious | Very serious | **Very low** | Risk of bias in contributing indirect evidence;  incoherence;  very serious network imprecision |
| **TC vs NT** | Indirect | NA | Moderate | Not serious | NA | Very serious | **Very low** | Risk of bias in contributing direct evidence;  very serious network imprecision |
| **TC vs DE** | Mixed | Moderate | Moderate | Not serious | Serious | Very serious | **Very low** | Risk of bias; incoherence;  very serious network imprecision |
| **TC vs UC** | Mixed | Moderate | Moderate | Not serious | Serious | Serious | **Very low** | Risk of bias; incoherence;  network imprecision |
| **SE vs NT** | Indirect | NA | Moderate | Not serious | NA | Very serious | **Very low** | Risk of bias in contributing direct evidence;  very serious network imprecision |
| **SE vs DE** | Indirect | NA | High | Not serious | NA | Very serious | **Low** | Very serious network imprecision |
| **SE vs UC** | Mixed | High | Moderate | Not serious | Serious | Serious | **Very low** | Risk of bias in contributing indirect evidence;  incoherence; network imprecision |
| **NT vs DE** | Mixed, direct-dominant | Moderate | Not informative | NA | Not assessable | Very serious | **Very low** | Risk of bias;  very serious network imprecision |
| **NT vs UC** | Indirect | NA | Moderate | Not serious | NA | Very serious | **Very low** | Risk of bias in contributing direct evidence;  very serious network imprecision |
| **DE vs UC** | Mixed | High | Moderate | Not serious | Serious | Serious | **Very low** | Risk of bias in contributing indirect evidence;  incoherence; network imprecision |

4.PDQ-39: Network imprecision was assessed using thresholds of −4.72 points for improvement and +4.22 points for worsening, as reported by Horváth et al. ^[4]^. These thresholds refer specifically to the PDQ-39 Summary Index and should not be applied directly to differently scored raw totals.

**HAMD-GRADE**

| **Comparison** | **Nature of the evidence** | **Direct certainty** | **Indirect certainty** | **Intransitivity** | **Incoherence** | **Network imprecision^5^** | **Final NMA certainty** | **Downgrade reason** |
| --- | --- | --- | --- | --- | --- | --- | --- | --- |
| **TC vs QG** | Indirect | NA | Moderate | Not serious | NA | Serious | **Low** | Risk of bias in contributing direct evidence;  network imprecision |
| **TC vs YG** | Indirect | NA | Moderate | Not serious | NA | Serious | **Low** | Risk of bias in contributing direct evidence;  network imprecision |
| **TC vs NT** | Direct, direct-dominant | Moderate | Not informative | NA | Not assessable | Not serious | **Moderate** | Risk of bias |
| **TC vs WE** | Indirect | NA | Moderate | Not serious | NA | Serious | **Low** | Risk of bias in contributing direct evidence;  network imprecision |
| **TC vs UC** | Indirect | NA | Moderate | Not serious | NA | Serious | **Low** | Risk of bias in contributing direct evidence;  network imprecision |
| **QG vs YG** | Indirect | NA | Moderate | Not serious | NA | Very serious | **Very low** | Risk of bias in contributing direct evidence;  very serious network imprecision |
| **QG vs NT** | Direct, direct-dominant | Moderate | Not informative | NA | Not assessable | Very serious | **Very low** | Risk of bias;  very serious network imprecision |
| **QG vs WE** | Direct, direct-dominant | High | Not informative | NA | Not assessable | Very serious | **Low** | Very serious network imprecision |
| **QG vs UC** | Direct, direct-dominant | Moderate | Not informative | NA | Not assessable | Serious | **Low** | Risk of bias;  network imprecision |
| **YG vs NT** | Indirect | NA | Moderate | Not serious | NA | Very serious | **Very low** | Risk of bias in contributing direct evidence;  very serious network imprecision |
| **YG vs WE** | Indirect | NA | Moderate | Not serious | NA | Very serious | **Very low** | Risk of bias in contributing direct evidence;  very serious network imprecision |
| **YG vs UC** | Direct, direct-dominant | High | Not informative | NA | Not assessable | Serious | **Moderate** | Network imprecision |
| **NT vs WE** | Indirect | NA | Moderate | Not serious | NA | Very serious | **Very low** | Risk of bias in contributing direct evidence;  very serious network imprecision |
| **NT vs UC** | Indirect | NA | Moderate | Not serious | NA | Very serious | **Very low** | Risk of bias in contributing direct evidence;  very serious network imprecision |
| **WE vs UC** | Indirect | NA | Moderate | Not serious | NA | Very serious | **Very low** | Risk of bias in contributing direct evidence;  very serious network imprecision |

5.HAMD: Network imprecision was provisionally assessed using a 3-point threshold, informed by the historical between-group criterion used in the 2004 NICE depression guideline and documented by Kirsch et al. ^[5,6]^. This was treated as an operational benchmark, not as a validated Parkinson’s disease–specific MCID; its applicability across HAMD versions remains uncertain.

**References**

[1]	Mehdizadeh M, Fereshtehnejad SM, Landers MR, et al. Responsiveness of the mini-balance evaluation systems test, dynamic gait index, Berg balance scale, and performance-oriented mobility assessment in parkinson’s disease. Scientific Reports. 2025;15:21126. doi:10.1038/s41598-025-08463-8.

[2]	Taghizadeh G, Eissazade N, Fereshtehnejad SM, et al. Minimal clinically important difference and substantial clinical benefits for single- and dual-task timed up and go test following motor-cognitive training in Parkinson’s disease. Age and Ageing. 2025;54(9). doi:10.1093/ageing/afaf241.

[3]	Schrag A, Sampaio C, Counsell N, Poewe W. Minimal clinically important change on the unified Parkinson’s disease rating scale. Movement Disorders. 2006;21(8):1200–1207. doi:10.1002/mds.20914.

[4]	Horváth K, Aschermann Z, Kovács M, et al. Changes in Quality of Life in Parkinson’s Disease: How Large Must They Be to Be Relevant? Neuroepidemiology. 2017;48(1–2):1–8. doi:10.1159/000455863.

[5]	National Institute for Clinical Excellence. Depression: management of depression in primary and secondary care. Clinical Guideline 23. London: National Institute for Clinical Excellence; 2004.

[6]	Kirsch I, Deacon BJ, Huedo-Medina TB, Scoboria A, Moore TJ, Johnson BT. Initial Severity and Antidepressant Benefits: A Meta-Analysis of Data Submitted to the Food and Drug Administration. PLoS Medicine. 2008;5(2). doi:10.1371/journal.pmed.0050045.

**Abbreviations and interpretation:**

NA: insufficient evidence to permit assessment

Not informative: an indirect estimate was theoretically available but was too imprecise to provide meaningful information for certainty assessment.

Not assessable: the relevant domain could not be evaluated reliably because one evidence component was extremely imprecise or otherwise uninformative.
