# Supplementary material for: Effects of mind–body exercise on motor function, quality of life, and depressive symptoms in patients with Parkinson’s disease: a systematic review and network meta-analysis
Source: Front Aging Neurosci. 2026 Oct 28;18:1982103. doi: 10.3389/fnagi.2026.1982103 (PMC13751929; doi:10.3389/fnagi.2026.1982103)
Supplement: Supplementary file 1 [file Data_Sheet_1.zip › Supplementary Material/Appendix 13-Results of sensitivity analysis..docx]

# **Table 8. Results of sensitivity analysis**

**PDQ-39(Random-effects sensitivity analysis)**

| **Intervention** | **SUCRA** | | | **RANK** | | |
| --- | --- | --- | --- | --- | --- | --- |
|  | **Fixed-effect** | **Random-effects** | | **Fixed-effect** | **Random-effects** | |
| Tai chi | **49.9%** | | **48.3%** | **4** | | **4** |
| Qigong | **82.6%** | | **92.5%** | **1** | | **1** |
| Yoga | **64.6%** | | **50.6%** | **3** | | **3** |
| Dance | **31.3%** | | **34.5%** | **7** | | **7** |

| **Qigong** |  |  |  |  |  |  |  |
| --- | --- | --- | --- | --- | --- | --- | --- |
| -0.64  (-1.88,0.61) | **Stretch** |  |  |  |  |  |  |
| -1.16  (-2.80,0.49) | -0.52  (-2.37,1.33) | **Yoga** |  |  |  |  |  |
| -1.21  (-2.64,0.22) | -0.58  (-2.12,0.97) | -0.05  (-1.70,1.59) | **Tai chi** |  |  |  |  |
| -1.29  (-2.98,0.40) | -0.66  (-2.65,1.34) | -0.14  (-2.27,2.00) | -0.08  (-2.00,1.84) | **Walking** |  |  |  |
| -1.40  (-3.99,1.20) | -0.76  (-3.49,1.97) | -0.24  (-2.99,2.50) | -0.19  (-2.73,2.36) | -0.10  (-2.81,2.60) | **No treatment** |  |  |
| -1.51  (-2.99,-0.03) | -0.87  (-2.58,0.83) | -0.35  (-2.08,1.37) | -0.30  (-1.68,1.08) | -0.22  (-1.88,1.45) | -0.11  (-2.25,2.02) | **Dance** |  |
| -1.85  (-2.94,-0.77) | -1.22  (-2.59,0.15) | -0.70  (-1.93,0.54) | -0.64  (-1.73,0.45) | -0.56 (-2.30,1.18) | -0.46  (-2.91,2.00) | -0.34  (-1.55,0.86) | **Usual care** |

**SMD（95%CI）**

**UPDRS-Ⅲ(Random-effects sensitivity analysis)**

| **Intervention** | **SUCRA** | | | **RANK** | | |
| --- | --- | --- | --- | --- | --- | --- |
|  | **Fixed-effect** | **Random-effects** | | **Fixed-effect** | **Random-effects** | |
| Tai chi | **51.6%** | | **57.1%** | **6** | | **5** |
| Qigong | **54%** | | **67.6%** | **5** | | **3** |
| Yoga | **84.1%** | | **85.6%** | **2** | | **1** |
| Dance | **67.2%** | | **67.9%** | **3** | | **2** |
| Pilates | **86.9%** | | **62.7%** | **1** | | **4** |

| **Yoga** |  |  |  |  |  |  |  |  |
| --- | --- | --- | --- | --- | --- | --- | --- | --- |
| -0.57  (-2.28,1.14) | **Dance** |  |  |  |  |  |  |  |
| -0.63  (-2.22,0.96) | -0.06  (-1.43,1.31) | **Qigong** |  |  |  |  |  |  |
| -0.58  (-2.87,1.70) | -0.01  (-2.15,2.12) | 0.05  (-1.99,2.09) | **Pilates** |  |  |  |  |  |
| -0.86  (-2.51,0.79) | -0.29  (-1.73,1.15) | -0.23  (-1.20,0.75) | -0.28  (-2.36,1.81) | **Tai chi** |  |  |  |  |
| -1.28  (-3.27,0.71) | -0.71  (-2.53,1.11) | -0.65  (-2.10,0.81) | -0.69  (-3.06,1.67) | -0.42  (-1.57,0.74) | **Walking** |  |  |  |
| -1.29  (-3.25,0.66) | -0.72  (-2.50,1.06) | -0.66  (-1.96,0.64) | -0.71  (-3.04,1.63) | -0.43  (-1.72,0.86) | -0.01  (-1.72,1.69) | **Stretch** |  |  |
| -1.45  (-2.78,-0.11) | -0.88  (-1.94,0.19) | -0.82  (-1.68,0.04) | -0.86  (-2.71,0.99) | -0.59  (-1.56,0.38) | -0.17  (-1.65,1.30) | -0.16  (-1.59,1.27) | **Usual care** |  |
| -1.60  (-3.31,0.10) | -1.03  (-2.53,0.47) | -0.97  (-1.93,-0.02) | -1.02  (-3.15,1.11) | -0.74  (-1.45,-0.03) | -0.33  (-1.57,0.92) | -0.31  (-1.69,1.06) | -0.15  (-1.21,0.90) | **No treatment** |

**SMD（95%CI）**

**UPDRS-Ⅲ(Fixed-effect analysis after exclusion of the high-risk study)**

| **Intervention** | **SUCRA** | | | **RANK** | | |
| --- | --- | --- | --- | --- | --- | --- |
|  | **Ago** | **After** | | **Ago** | **After** | |
| Tai chi | **51.6%** | | **55.2%** | **6** | | **5** |
| Qigong | **54%** | | **58.3%** | **5** | | **4** |
| Yoga | **84.1%** | | **61.9%** | **2** | | **3** |
| Dance | **67.2%** | | **71.5%** | **3** | | **2** |
| Pilates | **86.9%** | | **89%** | **1** | | **1** |

| **Pilates** |  |  |  |  |  |  |  |  |
| --- | --- | --- | --- | --- | --- | --- | --- | --- |
| -5.73  (-18.43,6.97) | **Dance** |  |  |  |  |  |  |  |
| -6.41  (-20.48,7.67) | -0.68  (-9.78,8.43) | **Yoga** |  |  |  |  |  |  |
| -7.19  (-20.59,6.20) | -1.46  (-9.49,6.57) | -0.78  (-10.83,9.26) | **Walking** |  |  |  |  |  |
| -7.50  (-19.79,4.80) | -1.77  (-7.77,4.24) | -1.09  (-9.62,7.44) | -0.31  (-6.51,5.90) | **Qigong** |  |  |  |  |
| -7.74  (-20.34,4.87) | -2.00  (-8.61,4.60) | -1.33  (-10.30,7.64) | -0.54  (-5.32,4.24) | -0.24  (-4.60,4.13) | **Tai chi** |  |  |  |
| -10.58  (-22.34,1.18) | -4.85  (-9.65,-0.05) | -4.17  (-11.90,3.56) | -3.39  (-9.80,3.03) | -3.08  (-6.67,0.51) | -2.84  (-7.39,1.70) | **Usual care** |  |  |
| -11.26  (-24.61,2.08) | -5.53  (-13.47,2.41) | -4.85  (-14.84,5.13) | -4.07  (-11.29,3.15) | -3.77  (-9.54,2.01) | -3.53  (-9.08,2.03) | -0.68  (-7.00,5.63) | **Stretch** |  |
| -12.19  (-24.88,0.50) | -6.46  (-13.23,0.32) | -5.78  (-14.87,3.31) | -5.00  (-10.17,0.18) | -4.69  (-8.92,-0.46) | -4.45  (-7.61,-1.29) | -1.61  (-6.38,3.16) | -0.92  (-6.92,5.07) | **No treatment** |

**MD（95%CI）**

**Colors in the table indicate the following:**

**light yellow, interventions;**

**light blue, effect estimates for pairwise comparisons between interventions that are not statistically significant;**

**and dark blue, statistically significant effect estimates for pairwise comparisons between interventions.**
