# Supplementary material for: Effects of mind–body exercise on motor function, quality of life, and depressive symptoms in patients with Parkinson’s disease: a systematic review and network meta-analysis
Source: Front Aging Neurosci. 2026 Oct 28;18:1982103. doi: 10.3389/fnagi.2026.1982103 (PMC13751929; doi:10.3389/fnagi.2026.1982103)
Supplement: Supplementary file 1 [file Data_Sheet_1.zip › Supplementary Material/Appendix 4-Included literture.docx]

# **Table 2. Characteristics of included studies**

| **Author** | **Publish  Year** | **Country** | **Research  Design** | **Sample Size** | | | **Age**（Mean±SD） | | | **Gender**（male/ female） | | | **Measure** | | | **Duration (weeks)** | **Frequency** | **Duration per Session** | **Hoehn**－Yahr | **Outcomes** |
| --- | --- | --- | --- | --- | --- | --- | --- | --- | --- | --- | --- | --- | --- | --- | --- | --- | --- | --- | --- | --- |
|  |  |  |  | **T1** | **T2** | **C** | **T1** | **T2** | **C** | **T1** | **T2** | **C** | **T1** | **T2** | **C** |  |  |  |  |  |
| Cao^[1]^ | 2021 | China | RCT | 31 | — | 31 | 62.45±2.87 | — | 62.97±3.27 | 20/11 | — | 21/10 | Qigong | — | Usual care | 8 | 5 times/week | 30min/session | 3 | ①②⑤ |
| Ding^[2]^ | 2023 | China | RCT | 42 | — | 42 | 69.34±4.02 | — | 69.12±4.06 | 24/18 | — | 26/16 | Tai chi | — | Usual care | 12 | 4 times/week | 60min/session | 2-2.5 | ① |
| Guan^[3]^ | 2018 | China | RCT | 40 | — | 40 | 69.46±5.45 | — | 68.61±6.22 | 23/17 | — | 21/19 | Tai chi | — | Usual care | 24 | 4 times/week | 60min/session | 1-2 | ①② |
| Guan^[4]^ | 2016 | China | RCT | 31 | — | 31 | 70.23±4.24 | — | 69.71±4.13 | 16/15 | — | 17/14 | Tai chi | — | Usual care | 12 | 4 times/week | 60min/session | 1-3 | ①② |
| Guan^[5]^ | 2017 | China | RCT | 50 | — | 50 | 68.75±3.42 | — | 69.56±3.75 | — | — | — | Yoga | — | Usual care | 24 | 3 times/week | 60min/session | 1-2 | ② |
| Ji^[6]^ | 2016 | China | RCT | 16 | — | 16 | 56.06±11.16 | — | 59.13±11.22 | 9/7 | — | 8/8 | Tai chi | — | NO treatment | 12 | 7 times/week | 60min/session | 1-3 | ①③ |
| Jiang^[7]^ | 2023 | China | RCT | 20 | — | 20 | 60.6±6.75 | — | 59.45±5.45 | 8/12 | — | 13/7 | Qigong | — | Usual care | 8 | 5 times/week | 30min/session | 1-3 | ③⑤ |
| Li^[8]^ | 2025 | China | RCT | 50 | — | 50 | 65.43±7.27 | — | 66.41±7.41 | 19/31 | — | 22/28 | Qigong | — | NO treatment | 12 | 5 times/week | 30min/session | 1-3 | ①③④ |
| Liu^[9]^ | 2017 | China | RCT | 23 | — | 18 | 57.2±8.1 | — | 52.7±5.5 | 9/14 | — | 6/12 | Qigong | — | NO treatment | 10 | 5 times/week | 60min/session | 1-3 | ②③ |
| Lu^[10]^ | 2024 | China | RCT | 20 | — | 20 | 63.65±5.28 | — | 64.05±4.77 | 11/9 | — | 10/10 | Qigong | — | Usual care | 4 | 7 times/week | 30min/session | 1-3 | ④ |
| Wang^[11]^ | 2016 | China | RCT | 40 | — | 40 | 67.6±8.4 | — | 68±8.5 | 19/21 | — | 20/20 | Tai chi | — | NO treatment | 16 | 14 times/week | 60min/session | 1-2 | ①③④ |
| Wang^[12]^ | 2025 | China | RCT | 35 | — | 37 | 70.54±6.71 | — | 74.00±7.15 | 18/17 | — | 23/14 | Qigong | — | Usual care | 12 | 2 times/week | 60min/session | 1-3 | ①③ |
| Wang^[13]^ | 2023 | China | RCT | 15 | — | 15 | 69.80±6.90 | — | 67.13±8.33 | 6/9 | — | 10/5 | Tai chi | — | NO treatment | 24 | 3 times/week | 60min/session | 1-4 | ①②③④ |
| Wu^[14]^ | 2018 | China | RCT | 28 | — | 24 | 62.42 ± 5. 37 | — | 64.66 ± 5.47 | 20/8 | — | 17/7 | Tai chi | — | Usual care | 16 | 4 times/week | 40min/session | 1-3 | ⑤ |
| Xiao^[15]^ | 2021 | China | RCT | 20 | — | 20 | 72.78±2.63 | — | 72.58± 2.62 | 9/11 | — | 8/12 | Tai chi | — | Usual care | 24 | 4 times/week | 60min/session | 1-2.5 | ① |
| Zhu^[16]^ | 2011 | China | RCT | 20 | — | 20 | 63∙35±8∙72 | — | 64∙83±9∙29 | 11/9 | — | 12/8 | Tai chi | — | Walking | 4 | 5 times/week | 40min/session | 1-2 | ①③ |
| Cherup^[17]^ | 2021 | America | RCT | 15 | — | 18 | 69.8±7.3 | — | 71.4±12.1 | 10/5 | — | 11/7 | Yoga | — | Usual care | 12 | 2 times/week | 45min/session | 1-3 | ② |
| Choi^[18]^ | 2013 | Korea | RCT | 11 | — | 9 | 60.81 ± 7.6 | — | 65.54 ± 6.8 | — | — | — | Tai chi | — | NO treatment | 12 | 1 times/week | 60min/session | 1-2 | ②③ |
| Çoban^[19]^ | 2021 | UK | RCT | 20 | — | 20 | 58.85 ± 8.09 | — | 60.75 ± 7.62 | 9/11 | — | 10/10 | Pilates | — | Usual care | 8 | 2 times/week | 45min/session | 2-3 | ①② |
| Frisaldi^[20]^ | 2020 | Italy | RCPT | 19 | — | 19 | 60.68±6.34 | — | 61.21±7.18 | 10/9 | — | 13/6 | Dance | — | Usual care | 5 | 3 times/week | 60min/session | 2 | ②⑤ |
| Gao^[21]^ | 2014 | China | RCT | 37 | — | 39 | 69.54±7.32 | — | 68.28±8.53 | 23/14 | — | 27/12 | Tai chi | — | Usual care | 12 | 3 times/week | 60min/session | 1-3 | ①②③ |
| Haas^[22]^ | 2024 | Brazil | RCT | 31 | — | 31 | 71,61±8,89 | — | 67.87±11 | 10/21 | — | 23/8 | Dance | — | Walking | 12 | 2 times/week | 60min/session | 1-3 | ②⑤ |
| Kwok^[23]^ | 2019 | China | RCT | 71 | — | 67 | 63.7±8.2 | — | 63.5±9.3 | 37/34 | — | 28/39 | Yoga | — | Stretch | 8 | 1 times/week | 90min/session | 1-3 | ② |
| [Li](https://pubmed.ncbi.nlm.nih.gov/?sort=date&size=200&term=Li+F&cauthor_id=22316445)^[24]^ | 2012 | America | RCT | 65 | — | 65 | 68±9 | — | 69±9 | 45/20 | — | 39/26 | Tai chi | — | Stretch | 24 | 2 times/week | 60min/session | 1-3 | ②③ |
| Li^[25]^ | 2022 | China | RCT | 17 | — | 19 | 67.4±6.06 | — | 69.16±6.17 | 8/9 | — | 9/10 | Dance | — | Usual care | 4 | 5 times/week | 60min/session | 1-3 | ①②③ |
| Li^[26]^ | 2022 | China | RCT | 32 | 31 | 32 | 62.7±5.51 | 61.9±5.64 | 61.9±6.76 | 17/15 | 22/9 | 19/13 | Tai chi | Walking | NO treatment | 24 | 2 times/week | 60min/session | 1-2.5 | ③ |
| Li^[27]^ | 2022 | China | RCPT | 20 | — | 20 | 67.57 ± 3.95 | — | 70± 5.59 | 13/7 | — | 16/4 | Qigong | — | Stretch | 12 | 2 times/week | 90min/session | 1-3 | ②⑤ |
| Cardalda^[28]^ | 2017 | Spain | RCT | 13 | — | 13 | 62.85 ± 9.75 | — | 66.00 ± 13.14 | 5/8 | — | 4/9 | Pilates | — | Usual care | 12 | 2 times/week | 60min/session | 1-3 | ②③ |
| Ni^[29]^ | 2016 | America | RCT | 15 | — | 10 | 71.2±6.5 | — | 74.9±8.3 | 11/4 | — | 6/6 | Yoga | — | Usual care | 12 | 2 times/week | 60min/session | 1-3 | ⑤ |
| Ni^[30]^ | 2015 | America | RCT | 13 | — | 10 | 72.1±4.5 | — | 73.8±7.5 | 11/2 | — | 4/6 | Yoga | — | Usual care | 12 | 2 times/week | 60min/session | 1-3 | ①②③ |
| Park^[31]^ | 2025 | Korea | RCT | 22 | 29 | 28 | 69.1±7.8 | 68.2±6.4 | 68.7±7.5 | 10/12 | 14/15 | 19/9 | Yoga | Tai chi | Usual care | 12 | 2 times/week | 60min/session | 1-3 | ② |
| Poier^[32]^ | 2019 | Germany | RCT | 14 | — | 15 | 68.50±8.07 | — | 68.87±10.96 | 9/5 | — | 3/12 | Dance | — | Tai chi | 10 | 1 times/week | 60min/session | 1-3 | ⑤ |
| Romenets^[33]^ | 2015 | Canada | RCT | 18 | — | 15 | 63.2±9.9 | — | 64.3±8.1 | 12/6 | — | 7/8 | Dance | — | NO treatment | 12 | 2 times/week | 60min/session | 1-3 | ②⑤ |
| Solla^[34]^ | 2018 | Italy | RCPT | 10 | — | 10 | 67.8 ± 5.9 | — | 67.1 ±6.3 | 男6女4 | — | 男7女3 | Dance | — | Usual care | 12 | 2 times/week | 90min/session | 1-3 | ①②③ |
| Sun^[35]^ | 2026 | China | RCT | 16 | — | 16 | 66.12±6.94 | — | 64.38±9.9 | 男9女7 | — | 男10女6 | Qigong | — | Walking | 12 | 5 times/week | 40min/session | 1-2.5 | ①④⑤ |
| Thunga^[36]^ | 2022 | India | RCT | 12 | — | 12 | 69±8.03 | — | 68.08±6.2 | 男8女4 | — | 男7女5 | Yoga | — | Usual care | 12 | 2 times/week | 60min/session | 1-3 | ③⑤ |
| Volpe^[37]^ | 2013 | Italy | RCPT | 12 | — | 12 | 61.6 ± 4.5 | — | 65.0 ± 5.3 | 男7女5 | — | 男6女6 | Dance | — | Usual care | 24 | 1 times/week | 90min/session | 0-2.5 | ①③⑤ |
| Wang^[38]^ | 2022 | China | RCT | 23 | — | 22 | 68.83 ± 4.35 | — | 67.95 ± 4.86 | 男7女16 | — | 男10女12 | Qigong | — | Stretch | 24 | 3 times/week | 90min/session | 1-2 | ②③⑤ |
| Vergara-Diaz^[39]^ | 2018 | America | RCPT | 16 | — | 16 | 65.7 ± 3.86 | — | 62 ± 7.77 | 男9女7 | — | 男7女9 | Tai chi | — | Usual care | 24 | 2 times/week | 60min/session | 2-2.5 | ②③⑤ |
| Xiao^[40]^ | 2015 | China | RCT | 48 | — | 48 | 68.17±2.27 | — | 66.52±2.13 | 男34女14 | — | 男33女15 | Qigong | — | Usual care | 24 | 4 times/week | 45min/session | 1-3 | ①②③ |
| [Xu](https://pubmed.ncbi.nlm.nih.gov/?sort=date&size=200&term=Xu+Q&cauthor_id=41469986)^[41]^ | 2025 | China | RCT | 44 | — | 46 | 71.43±6.33 | — | 71.2±7.44 | 男32女12 | — | 男33女13 | Yoga | — | Usual care | 8 | 5 times/week | 20min/session | 0-3 | ④⑤ |
| Yin^[42]^ | 2025 | China | RCT | 25 | — | 26 | 58.8±8.61 | — | 58.15±7.09 | 男12女13 | — | 男12女14 | Qigong | — | Usual care | 12 | 5 times/week | 30min/session | 1-3 | ④⑤ |
| [Zhang](https://pubmed.ncbi.nlm.nih.gov/?sort=date&size=200&term=Zhang+W&cauthor_id=42082156)^[43]^ | 2026 | China | RCT | 15 | 15 | 15 | 64.33 ± 6.42 | 62.27±4.33 | 63.93 ± 5.38 | 男7女8 | 男10女5 | 男6女9 | Tai chi | Stretch | Usual care | 12 | 3 times/week | 60min/session | 2-3 | ②⑤ |

①=BBS;②=TUG;③=UPDRS-Ⅲ;④=HAMD;⑤PDQ-39

[1]	Cao H H, Sun W Y, Xi X M, Wang L M, Bi H Y. Effects of Wuqinxi on Balance, Walking and Quality of Life for Patients with Parkinson's Disease. Chinese Journal of Rehabilitation Theory and Practice 27 (2021) 1087-1092.

[2]	Ding L, Chen J L, Yu X M, Wan X W, Xiong J. Effect of Tai Chi Exercise on Mental Health and Balance Ability in Patients with Parkinson's Disease. Shanghai Nursing 23 (2023) 19-21.

[3]	Guan X H, Tang X Z, Dong Y H. The Effects of Tai Chi Training on Walking Ability and Fear of Falling in Early Parkinson's Patients. Chinese Journal of Gerontology 38 (2018) 4962-4963.

[4]	Guan X H, Tang X Z, Liu J M. Effect of Tai Chi training on walking ability and fear of falling of patients with Parkinson's disease. Chinese Nursing Research 30 (2016) 3514-3517.

[5]	Guan X H, Zhu. X G, Liu J M, Liu Z Q, Zhai S Z. Influence of Yoga on balancing ability and fear of falling in patients with early Parkinson's disease. Chinese Nursing Research 31 (2017) 1274-1276.

[6]	Ji S Q, Mao Z J, Yang Q, Gao H L, Xue Z. Effectiveness of Tai Chi for Parkinson disease. Chinese Journal of Rehabilitation 31 (2016) 51-53.

[7]	Jiang J H, Bi H Y. Influence of fitness qigong Yijinjing on fatigue and quality of life in patients with Parkinson's disease. Chinese Nursing Research 37 (2023) 4452-4457.

[8]	Li Q, Liu H M, Wang W T, Zhang S, Xie B C, Chen L, et. aI. Effect of Baduanjin on mood and sleep quality in patients with mild to moderate Parkinson disease. Journal of Apoplexy and Nervous Diseases 42 (2025) 200-203+289.

[9]	Liu X L, Wan Z R, Shang M Q, Yang L X, Xin Y. Effects of Health Qigong on treatments of Parkinson's disease. Chinese Journal of Neuroimmunology and Neurology 24 (2017) 34-37.

[10]	Lu C X, Chen Y J, Fu H P, Dong K, Zeng Y Q, Mo X R. Observation of the Effect of Combining Baduanjin with Home-Based Parkinson's Rehabilitation Exercises on Negative Emotions in Patients with Early to Mid-Stage Parkinson's Disease. Reflexology and Rehabilitation Medicine 5 (2024) 64-67.

[11]	Wang J Z, Peng Y J, Zhen Z X. Research of the Effect of Taijiquan Sport in the Treatment of Early Parkinson's Patients with Depression. The Journal of Medical Theory and Practice 29 (2016) 3309-3311.

[12]	Wang J X, Ning L, Yang X Y, Wu H J, Wu J Y, Zhen Y, et. aI. Application of Chinese sexagenary cycle auricular theory based on meridian theory combined with Wuqinxi in patients with mild to moderate Parkinson's disease. Journal of Nursing(China) 32 (2025) 67-74.

[13]	Wang M H, Gan M, Wu H Q, Yu D X, Chen Z H, Qian P A, et. aI. Efficacy of Tai Chi Exercise With Different Training Loads on The Rehabilitation of Patients With Parkinson's Disease in The Early and Middle Stage. Progress in Biochemistry and Biophysics 50 (2023) 2487-2495.

[14]	Wu T T, Wang Y Q, Luo X R, Ye S. Effects of Tai Chi exercise on cognition and health-related quality of life in patients with Parkinson's disease. Chinese Journal of Rehabilitation 33 (2018) 95-97.

[15]	Xiao H L, Hong A H, Ma X Z. Observation of the Effect of Tai Chi on Improving Balance Disorders in Early Parkinson's Patients. Traditional Chinese Medicine Rehabilitation 12 (2021) 41-42+44.

[16]	Zhu Y, Li J X, Li N, Jin H Z, Hua L, Dong Q. Effect of Taijiquan on Motion Control for Parkinson's Disease at Early Stage. Chinese Journal of Rehabilitation Theory and Practice 17 (2011) 355-358.

[17]	N P Cherup, K L Strand, L Lucchi, S V Wooten, C Luca, J F Signorile. Yoga Meditation Enhances Proprioception and Balance in Individuals Diagnosed With Parkinson's Disease. Percept Mot Skills 128 (2021) 304-323.

[18]	H J Choi, C E Garber, T W Jun, Y S Jin, S J Chung, H J Kang. Therapeutic effects of tai chi in patients with Parkinson's disease. ISRN Neurol 2013 (2013) 548240.

[19]	F Çoban, B Belgen Kaygısız, F Selcuk. Effect of clinical Pilates training on balance and postural control in patients with Parkinson's disease: a randomized controlled trial. J Comp Eff Res 10 (2021) 1373-1383.

[20]	E Frisaldi, P Bottino, M Fabbri, M Trucco, A De Ceglia, N Esposito, et. al. Effectiveness of a dance-physiotherapy combined intervention in Parkinson's disease: a randomized controlled pilot trial. Neurol Sci 42 (2021) 5045-5053.

[21]	Q Gao, A Leung, Y Yang, Q Wei, M Guan, C Jia, et.al. Effects of Tai Chi on balance and fall prevention in Parkinson's disease: a randomized controlled trial. Clin Rehabil 28 (2014) 748-753.

[22]	A N Haas, M D S Delabary, E Passos-Monteiro, M Wolffenbuttel, R G Donida, M Z Casal, et.al. The effects of Brazilian dance, deep-water exercise and nordic walking, pre- and post-12 weeks, on functional-motor and non-motor symptoms in trained PwPD. Arch Gerontol Geriatr 118 (2024) 105285.

[23]	J Y Y Kwok, J C Y Kwan, M Auyeung, V C T Mok, C K Y Lau, K C Choi, et.al. Effects of Mindfulness Yoga vs Stretching and Resistance Training Exercises on Anxiety and Depression for People With Parkinson Disease: A Randomized Clinical Trial. JAMA Neurol 76 (2019) 755-763.

[24]	F Z Li, P Harmer, K Fitzgerald, E Eckstrom, R Stock, J Galver, et.al. Tai chi and postural stability in patients with Parkinson's disease. N Engl J Med 366 (2012) 511-9.

[25]	F Li, D Wang, X Ba, Z Liu, M Zhang. The comparative effects of exercise type on motor function of patients with Parkinson’s disease: A three-arm randomized trial. Frontiers in Human Neuroscience Volume 16 - 2022 (2022).

[26]	G Li, P Huang, S S Cui, Y Y Tan, Y C He, X Shen, et.al . Mechanisms of motor symptom improvement by long-term Tai Chi training in Parkinson's disease patients. Transl Neurodegener 11 (2022) 6.

[27]	Z Li, T Wang, M Shen, T Song, J He, W Guo, et.al. Comparison of Wuqinxi Qigong with Stretching on Single- and Dual-Task Gait, Motor Symptoms and Quality of Life in Parkinson's Disease: A Preliminary Randomized Control Study. Int J Environ Res Public Health 19 (2022).

[28]	I Mollinedo-Cardalda, J.M. Cancela-Carral, and M.H. Vila-Suárez, Effect of a Mat Pilates Program with TheraBand on Dynamic Balance in Patients with Parkinson's Disease: Feasibility Study and Randomized Controlled Trial. Rejuvenation Res 21 (2018) 423-430.

[29]	M Ni, K Mooney, J F Signorile. Controlled pilot study of the effects of power yoga in Parkinson's disease. Complement Ther Med 25 (2016) 126-31.

[30]	M Ni, J F Signorile, K Mooney, A Balachandran, M Potiaumpai, C Luca, et.al. Comparative Effect of Power Training and High-Speed Yoga on Motor Function in Older Patients With Parkinson Disease. Arch Phys Med Rehabil 97 (2016) 345-354.e15.

[31]	J Park, S M Cheon, M J Lee, D W Ryu, D Yoo. Comparison of the Impact of Various Exercise Modalities on Parkinson's Disease. J Mov Disord 18 (2025) 222-230.

[32]	D Poier, D Rodrigues Recchia, T Ostermann, A Büssing. A Randomized Controlled Trial to Investigate the Impact of Tango Argentino versus Tai Chi on Quality of Life in Patients with Parkinson Disease: A Short Report. Complement Med Res 26 (2019) 398-403.

[33]	S Rios Romenets, J Anang, S M Fereshtehnejad, A Pelletier, R Postuma. Tango for treatment of motor and non-motor manifestations in Parkinson's disease: a randomized control study. Complement Ther Med 23 (2015) 175-84.

[34]	P Solla, L Cugusi, M Bertoli, A Cereatti, U Della Croce, D Pani, et.al. Sardinian Folk Dance for Individuals with Parkinson's Disease: A Randomized Controlled Pilot Trial. J Altern Complement Med 25 (2019) 305-316.

[35]	Y Sun, P Tian, Y Xie, Y Guo, F Liu, H Xing. Comparative effects of Baduanjin versus brisk walking on postural control and multidimensional functions in early-to-mid-stage Parkinson's Disease: a randomized controlled trial. Front Neurol 17 (2026) 1792257.

[36]	S Karthikbabu, V Prem, A Thunga. Comparison of yoga and physiotherapy on motor neuropsychiatric symptoms and quality of life in Parkinson’s disease. Annals of Movement Disorders 5 (2022) 55-64.

[37]	D Volpe, M Signorini, A Marchetto, T Lynch, M E Morris. A comparison of Irish set dancing and exercises for people with Parkinson’s disease: A phase II feasibility study. BMC Geriatrics 13 (2013) 54.

[38]	Z Wang, Y Pi, X Tan, Z Wang, R Chen, Y Liu, et.al. Effects of Wu Qin Xi exercise on reactive inhibition in Parkinson’s disease: A randomized controlled clinical trial. Frontiers in Aging Neuroscience Volume 14 - 2022 (2022).

[39]	G Vergara-Diaz, K Osypiuk, J M Hausdorff, P Bonato, B J Gow, J G Miranda, et.al. Tai Chi for Reducing Dual-task Gait Variability, a Potential Mediator of Fall Risk in Parkinson's Disease: A Pilot Randomized Controlled Trial. Glob Adv Health Med 7 (2018) 2164956118775385.

[40]	C M Xiao, and Y C Zhuang. Effect of health Baduanjin Qigong for mild to moderate Parkinson's disease. Geriatr Gerontol Int 16 (2016) 911-9.

[41]	Q Xu, X Nian, Y Pan, L Zhang, S Lin, S Zheng, et.al. Effect of laughter yoga on anxiety and depression in people with Parkinson’s disease: a randomized controlled trial. BMC Complementary Medicine and Therapies 26 (2025) 103.

[42]	H Yin, O Cheng, X Zhang, F Quan, Y Zhang, H Zuo, et.al. Effects of Liuzijue Qigong on respiratory function among patients with Parkinson’s disease: a randomized clinical trial. BMC Complementary Medicine and Therapies 25 (2025) 63.

[43]	W Zhang, X Xie, F Zhong, W Lv, H-Y Lai, C Huang. Effects of Tai Chi on core motor symptoms in patients with Parkinson’s disease: A three−armed randomized controlled trial. Behavioural Brain Research 510 (2026) 116251.
