# Supplementary material for: Effects of mind–body exercise on motor function, quality of life, and depressive symptoms in patients with Parkinson’s disease: a systematic review and network meta-analysis
Source: Front Aging Neurosci. 2026 Oct 28;18:1982103. doi: 10.3389/fnagi.2026.1982103 (PMC13751929; doi:10.3389/fnagi.2026.1982103)
Supplement: Supplementary file 1 [file Data_Sheet_1.zip › Supplementary Material/Appendix 5-Risk of bias assessment..docx]

# **Table 3. Risk of Bias Assessment**

| **Study**  **ID** | **Outcomes assessed** | **Randomization process** | **Deviations from intended interventions** | **Missing outcome data** | **Measurement of the outcome** | **Selection of the reported result** | **Overall** |
| --- | --- | --- | --- | --- | --- | --- | --- |
| **Cao 2021** | **①②⑤** | Low risk^1^ | Some concerns^4^ | Low risk^5^ | Low risk^8^ | Some concerns^11^ | Some concerns^13^ |
| **Ding 2023** | **①** | Low risk^1^ | Some concerns^4^ | Low risk^5^ | Low risk^8^ | Some concerns^11^ | Some concerns^13^ |
| **Guan 2018** | **①②** | Low risk^1^ | Low risk^3^ | Low risk^5^ | Low risk^8^ | Low risk^10^ | Low risk^12^ |
| **Guan 2016** | **①②** | Low risk^1^ | Some concerns^4^ | Low risk^5^ | Low risk^8^ | Some concerns^11^ | Some concerns^13^ |
| **Guan 2017** | **②** | Low risk^1^ | Some concerns^4^ | Low risk^5^ | Low risk^8^ | Some concerns^11^ | Some concerns^13^ |
| **Ji 2016** | **①③** | Some concerns^2^ | Some concerns^4^ | Low risk^5^ | Some concerns^9^ | Some concerns^11^ | Some concerns^13^ |
| **Jiang 2023** | **③⑤** | Low risk^1^ | Low risk^3^ | Low risk^5^ | Low risk^8^ | Some concerns^11^ | Some concerns^13^ |
| **Li 2025** | **①③④** | Low risk^1^ | Low risk^3^ | Low risk^5^ | Low risk^8^ | Some concerns^11^ | Some concerns^13^ |
| **Liu 2017** | **②③** | Some concerns^2^ | Some concerns^4^ | Low risk^5^ | Some concerns^9^ | Some concerns^11^ | Some concerns^13^ |
| **Lu 2024** | **④** | Low risk^1^ | Low risk^3^ | Low risk^5^ | Low risk^8^ | Some concerns^11^ | Some concerns^13^ |
| **Wang 2016** | **①③④** | Low risk^1^ | Some concerns^4^ | Low risk^5^ | Some concerns^9^ | Some concerns^11^ | Some concerns^13^ |
| **Wang 2025** | **①③** | Low risk^1^ | Low risk^3^ | Some concerns^6^ | Low risk^8^ | Some concerns^11^ | Some concerns^13^ |
| **Wang 2023** | **①②③④** | Low risk^1^ | Low risk^3^ | Low risk^5^ | Low risk^8^ | Some concerns^11^ | Some concerns^13^ |
| **Wu 2018** | **⑤** | Some concerns^2^ | Some concerns^4^ | Low risk^5^ | Some concerns^9^ | Some concerns^11^ | Some concerns^13^ |
| **Xiao 2021** | **①** | Low risk^1^ | Some concerns^4^ | Low risk^5^ | Low risk^8^ | Some concerns^11^ | Some concerns^13^ |
| **Zhu 2011** | **①③** | Low risk^1^ | Some concerns^4^ | Some concerns^6^ | Low risk^8^ | Some concerns^11^ | Some concerns^13^ |
| **Cherup 2021** | **②** | Low risk^1^ | Some concerns^4^ | Low risk^5^ | Low risk^8^ | Low risk^10^ | Some concerns^13^ |
| **Choi 2013** | **②③** | Low risk^1^ | Some concerns^4^ | Low risk^5^ | Low risk^8^ | Low risk^10^ | Some concerns^13^ |
| **Çoban 2021** | **①②** | Low risk^1^ | Some concerns^4^ | Low risk^5^ | Low risk^8^ | Low risk^10^ | Some concerns^13^ |
| **Frisaldi 2020** | **②⑤** | Low risk^1^ | Low risk^3^ | Low risk^5^ | Low risk^8^ | Low risk^10^ | Low risk^12^ |
| **Gao 2014** | **①②③** | Low risk^1^ | Low risk^3^ | Low risk^5^ | Low risk^8^ | Low risk^10^ | Low risk^12^ |
| **Haas 2024** | **②⑤** | Low risk^1^ | Some concerns^4^ | Low risk^5^ | Low risk^8^ | Low risk^10^ | Some concerns^13^ |
| **Kwok 2019** | **②** | Low risk^1^ | Some concerns^4^ | Low risk^5^ | Low risk^8^ | Low risk^10^ | Some concerns^13^ |
| **[Li](https://pubmed.ncbi.nlm.nih.gov/?sort=date&size=200&term=Li+F&cauthor_id=22316445) 2012** | **②③** | Low risk^1^ | Low risk^3^ | Low risk^5^ | Low risk^8^ | Low risk^10^ | Low risk^12^ |
| **[Li](https://pubmed.ncbi.nlm.nih.gov/?sort=date&size=200&term=Li+F&cauthor_id=22316445) 2022** | **①②③** | Some concerns^2^ | Some concerns^4^ | Some concerns^6^ | Low risk^8^ | Some concerns^11^ | Some concerns^13^ |
| **[Li](https://pubmed.ncbi.nlm.nih.gov/?sort=date&size=200&term=Li+F&cauthor_id=22316445) 2022** | **③** | Low risk^1^ | Some concerns^4^ | Low risk^5^ | Low risk^8^ | Low risk^10^ | Some concerns^13^ |
| **[Li](https://pubmed.ncbi.nlm.nih.gov/?sort=date&size=200&term=Li+F&cauthor_id=22316445) 2022** | **②⑤** | Low risk^1^ | Low risk^3^ | Low risk^5^ | Low risk^8^ | Low risk^10^ | Low risk^12^ |
| **Cardalda 2017** | **②③** | Low risk^1^ | Low risk^3^ | Low risk^5^ | Low risk^8^ | Low risk^10^ | Low risk^12^ |
| **Ni 2016** | **⑤** | Low risk^1^ | Some concerns^4^ | Some concerns^6^ | Some concerns^9^ | Some concerns^11^ | Some concerns^13^ |
| **Ni 2015** | **①②③** | Low risk^1^ | Some concerns^4^ | High risk^7^ | Low risk^8^ | Some concerns^11^ | High risk^14^ |
| **Park 2025** | **②** | Some concerns^2^ | Some concerns^4^ | Low risk^5^ | Some concerns^9^ | Low risk^10^ | Some concerns^13^ |
| **Poier 2019** | **⑤** | Low risk^1^ | Some concerns^4^ | Low risk^5^ | Low risk^8^ | Low risk^10^ | Some concerns^13^ |
| **Romenets 2015** | **②⑤** | Low risk^1^ | Some concerns^4^ | Low risk^5^ | Low risk^8^ | Low risk^10^ | Some concerns^13^ |
| **Solla 2018** | **①②③** | Low risk^1^ | Low risk^3^ | Low risk^5^ | Some concerns^9^ | Low risk^10^ | Some concerns^13^ |
| **Sun 2026** | **①④⑤** | Low risk^1^ | Low risk^3^ | Low risk^5^ | Low risk^8^ | Low risk^10^ | Low risk^12^ |
| **Thunga 2022** | **③⑤** | Low risk^1^ | Some concerns^4^ | Low risk^5^ | Low risk^8^ | Low risk^10^ | Some concerns^13^ |
| **Volpe 2013** | **①③⑤** | Low risk^1^ | Low risk^3^ | Low risk^5^ | Low risk^8^ | Low risk^10^ | Low risk^12^ |
| **Wang 2022** | **②③⑤** | Low risk^1^ | Low risk^3^ | Low risk^5^ | Low risk^8^ | Low risk^10^ | Low risk^12^ |
| **Vergara-Diaz 2018** | **②③⑤** | Low risk^1^ | Low risk^3^ | Low risk^5^ | Low risk^8^ | Low risk^10^ | Low risk^12^ |
| **Xiao 2015** | **①②③** | Low risk^1^ | Low risk^3^ | Some concerns^6^ | Low risk^8^ | Low risk^10^ | Some concerns^13^ |
| **[Xu](https://pubmed.ncbi.nlm.nih.gov/?sort=date&size=200&term=Xu+Q&cauthor_id=41469986) 2025** | **④⑤** | Low risk^1^ | Low risk^3^ | Low risk^5^ | Low risk^8^ | Low risk^10^ | Low risk^12^ |
| **Yin 2025** | **④⑤** | Low risk^1^ | Low risk^3^ | Low risk^5^ | Low risk^8^ | Low risk^10^ | Low risk^12^ |
| **[Zhang](https://pubmed.ncbi.nlm.nih.gov/?sort=date&size=200&term=Zhang+W&cauthor_id=42082156) 2026** | **②⑤** | Low risk^1^ | Low risk^3^ | Low risk^5^ | Low risk^8^ | Low risk^10^ | Low risk^12^ |

**Notes:①BBS,②TUG,③UPDRS-Ⅲ，④HAMD,⑤PDQ-39**

**The table uses color coding to indicate risk levels: green, low risk; yellow, some concerns; and red, high risk.**

**1.Appropriate random sequence generation and allocation concealment were reported, with no baseline imbalances suggesting a problem with randomization.**

**2.Allocation concealment was not reported, raising some concerns about the randomization process.**

**3.Although blinding was not feasible, no deviations from the intended interventions arising from the trial context were identified, and an appropriate analysis was used to estimate the effect of assignment to intervention.**

**4.Although an appropriate analysis was used to estimate the effect of assignment to intervention, blinding was not feasible, and reporting was insufficient to determine whether deviations from the intended interventions arose because of the trial context.**

**5.Outcome data were available for all or nearly all randomized participants.**

**6.Some outcome data were missing, and it was unclear whether missingness depended on the true outcome values.**

**7.Missingness was likely to depend on the true outcome values, and there was insufficient evidence that the result was unbiased despite the missing data.**

**8.Outcomes were measured using appropriate and consistent methods across groups by assessors blinded to treatment allocation.**

**9.It was unclear whether outcome assessors were aware of intervention allocation or whether such awareness could have influenced outcome measurement.**

**10.The analysis followed a prespecified plan finalized before unblinded outcome data were available, with no indication of result-based selection among eligible outcome measurements or analyses.**

**11.No prespecified analysis plan was available to verify whether the reported result had been selected from multiple eligible outcome measurements or analyses.**

**12.All five domains were judged to be at low risk of bias.**

**13.Some concerns were identified in one or more domains, but no domain was rated as high risk, and the combined concerns did not substantially lower confidence in the result.**

**14.The overall judgment was “High risk” because at least one domain was rated as high risk of bias.**

**Within each study, the listed outcome results were assessed separately and yielded identical domain-level and overall risk-of-bias judgments; they are therefore presented together.**

As percentage (intention-to-treat)

|  | Low risk | Some concerns | High risk |
| --- | --- | --- | --- |
| Randomization process | 88.4 | 11.6 | 0 |
| Deviations from intended interventions | 46.5 | 53.5 | 0 |
| Mising outcome data | 86 | 11.6 | 2.3 |
| Measurement of the outcome | 83.7 | 16.3 | 0 |
| Selection of the reported result | 58.1 | 41.9 | 0 |
| Overall Bias | 30.2 | 67.4 | 2.3 |

Figure 2 Distribution of risk of bias across included studies, as assessed by the Cochrane Rob 2.0 Risk of Bias tool.
