# Supplementary material for: Effects of mind–body exercise on motor function, quality of life, and depressive symptoms in patients with Parkinson’s disease: a systematic review and network meta-analysis
Source: Front Aging Neurosci. 2026 Oct 28;18:1982103. doi: 10.3389/fnagi.2026.1982103 (PMC13751929; doi:10.3389/fnagi.2026.1982103)
Supplement: Supplementary file 1 [file Data_Sheet_1.zip › Supplementary Material/Appendix 6-Assessment of transitivity.docx]

**Table 4.** Assessment of transitivity

| **Direct comparison** | **Number of studies** | **Age^1^**  **(Reported mean age)** | **Hoehn－Yahr** | **Duration**  **(weeks)** | **Frequency** |
| --- | --- | --- | --- | --- | --- |
| TC vs UC | 9 | 62-73 | 1-3 | 12-24 | 2-4 times/week |
| TC vs NT | 5 | 56-70 | 1-4 | 12-24 | 1-14 times/week |
| TC vs WE | 2 | 62-65 | 1-2.5 | 4-24 | 2-5 times/week |
| TC vs SE | 2 | 62-69 | 1-3 | 12-24 | 2-3 times/week |
| TC vs YG | 1 | 68-69 | 1-3 | 12 | 2 times/week |
| TC vs DE | 1 | 68 | 1-3 | 10 | 1 times/week |
| QG vs UC | 6 | 58-74 | 1-3 | 4-24 | 2-7 times/week |
| QG vs NT | 2 | 53-66 | 1-3 | 10-12 | 5 times/week |
| QG vs WE | 1 | 64-66 | 1-2.5 | 12 | 5 times/week |
| QG vs SE | 2 | 68-70 | 1-3 | 12-24 | 2-3 times/week |
| DE vs UC | 4 | 61-69 | 0-3 | 4-24 | 1-5 times/week |
| DE vs NT | 1 | 63-64 | 1-3 | 12 | 2 times/week |
| DE vs WE | 1 | 68-72 | 1-3 | 12 | 2 times/week |
| YG vs UC | 7 | 68-75 | 0-3 | 8-24 | 2-5 times/week |
| YG vs SE | 1 | 63 | 1-3 | 8 | 1 times/week |
| PE vs UC | 2 | 59-66 | 1-3 | 8-12 | 2 times/week |
| WE vs NT | 1 | 62 | 1-2.5 | 24 | 2 times/week |
| SE vs UC | 1 | 62-64 | 2-3 | 12 | 3 times/week |

1.Age ranges represent the minimum and maximum of the reported study-arm mean ages, rather than the individual participant age ranges.

| **Direct comparison** | **Number of studies** | **Range of reported baseline means** | | | | | **Disease duration**  **(year)** | **Medication status/ assessment state** |
| --- | --- | --- | --- | --- | --- | --- | --- | --- |
|  |  | **BBS** | **TUG（s）** | **UPDRS-Ⅲ** | **HAMD** | **PDQ-39** |  |  |
| TC vs UC | 9 | 40.98-46.03 | 8.94-21.41 | 20.63-31.86 | NR | 16.3-41.91 | 1.07-9.06 | Stable medication;  mixed ON/OFF/NR |
| TC vs NT | 5 | 44.21-52.63 | 7.39-14.89 | 13.06-29.73 | 13.42-22.68 | NR | 2.09-5.91 | Stable medication;  mixed ON/OFF/NR |
| TC vs WE | 2 | 44.11-44.47 | NR | 17.5-25.2 | NR | NR | 2.72-5.91 | Stable medication;  mixed ON/NR |
| TC vs SE | 2 | NR | 8.6-11.56 | 15.06-15.28 | NR | 19.4-21.27 | 5.73-8 | Stable medication;  mixed ON/OFF |
| TC vs YG | 1 | NR | 8.94-9.61 | NR | NR | NR | NR | NR |
| TC vs DE | 1 | NR | NR | NR | NR | 25.06-28.13 | NR | NR |
| QG vs UC | 6 | 33.94-49.2 | 12.1-24.45 | 20.27-27.4 | 7.54-21.33 | 23.34-87.74 | 3.85-6.15 | Stable medication;  mixed ON/OFF/NR |
| QG vs NT | 2 | 47.78-48.7 | 9.19-11.19 | 32.54-35 | 13.57-13.81 | NR | 3.9-5 | Stable medication;  mixed ON/NR |
| QG vs WE | 1 | 48.69-50.56 | NR | NR | 7.58-9.13 | 13.06-13.5 | 5.29-5.59 | Stable medication;  ON |
| QG vs SE | 2 | NR | 9.96-12.59 | 20.78-21.18 | NR | 25.78-30.32 | 6.09-7.76 | Stable medication;  mixed ON/NR |
| DE vs UC | 4 | 34.08-46.35 | 6.56-14.24 | 13-24.58 | NR | 15.49-32.58 | 3.65-9 | Stable medication;  mixed ON/NR |
| DE vs NT | 1 | NR | 7.4-7.9 | NR | NR | 25.8-26.8 | 5.5-7.7 | Stable medication;  NR |
| DE vs WE | 1 | NR | 9.72-11.7 | NR | NR | 31.87-34.35 | 5.61-7.03 | Stable medication;  NR |
| YG vs UC | 7 | 49.22-50.6 | 7.42-10.27 | 15.25-30.8 | 9.04-9.34 | 28.07-44.2 | 1.23-6.9 | Stable medication;  mixed ON/OFF/NR |
| YG vs SE | 1 | NR | 14.05-17.54 | 31.64-34.9 | NR | NR | NR | Stable medication;  NR |
| PE vs UC | 2 | 45.1-47.45 | 11.83-13 | 29.55-31.54 | NR | NR | 5.32-5.77 | Stable medication;  mixed ON/OFF/NR |
| WE vs NT | 1 | NR | NR | 17.5-19.3 | NR | NR | 3.82-4.32 | Stable medication;  NR |
| SE vs UC | 1 | NR | 10.6-10.67 | NR | NR | 21.27-30.67 | 5.73-7.17 | Stable medication;  NR |

ON=ON medication;OFF=OFF medication;NR=assessment state not reported

Assessment state：the medication state at the time of outcome assessment, including ON-medication state, OFF-medication state, or not reported (NR).
