# Supplementary material for: Effects of mind–body exercise on motor function, quality of life, and depressive symptoms in patients with Parkinson’s disease: a systematic review and network meta-analysis
Source: Front Aging Neurosci. 2026 Oct 28;18:1982103. doi: 10.3389/fnagi.2026.1982103 (PMC13751929; doi:10.3389/fnagi.2026.1982103)
Supplement: Supplementary file 1 [file Data_Sheet_1.zip › Supplementary Material/Appendix 7-Results of the Node-Splitting model.docx]

# **Table 5. Results of the Node-Splitting model**

**BBS**

|  | **Direct Indirect Difference** | | | | | | **p＞Z** |
| --- | --- | --- | --- | --- | --- | --- | --- |
|  | **Coefficient SE Coefficient SE Coefficient SE** | | | | | |  |
| **TC vs UC** | -4.269 | 0.677 | -4.29 | 1.466 | 0.021 | 1.617 | 0.990 |
| **TC vs NT** | -2.438 | 0.757 | -2.905 | 1.9 | 0.467 | 2.051 | 0.820 |
| **TC vs WE** | -3 | 1.459 | -2.54 | 1.36 | -0.46 | 1.995 | 0.817 |
| **QG vs UC** | -3.232 | 0.674 | -3.205 | 1.47 | -0.027 | 1.619 | 0.987 |
| **QG vs NT** | -1.78 | 1.713 | -1.313 | 1.127 | -0.467 | 2.05 | 0.820 |
| **QG vs WE** | -1.58 | 1.057 | -2.038 | 1.692 | 0.458 | 1.995 | 0.819 |
| **YG vs UC** | -2.12 | 1.274 | -8.29 | 137.399 | 6.17 | 137.4 | 0.964 |
| **DE vs UC** | -5.196 | 1.272 | -9.327 | 181.655 | 4.131 | 181.661 | 0.982 |
| **PE vs UC** | -3.25 | 2.381 | -8.566 | 393.116 | 5.316 | 393.133 | 0.989 |

**TUG**

|  | **Direct Indirect Difference** | | | | | | **p＞Z** |
| --- | --- | --- | --- | --- | --- | --- | --- |
|  | **Coefficient SE Coefficient SE Coefficient SE** | | | | | |  |
| **TC vs YG** | -0.943 | 1.012 | -0.012 | 0.746 | -0.931 | 1.255 | 0.458 |
| **TC vs UC** | 1.262 | 0.496 | 1.108 | 0.926 | 0.154 | 1.049 | 0.883 |
| **TC vs NT** | 0.962 | 0.851 | 1.484 | 0.997 | -0.522 | 1.308 | 0.690 |
| **TC vs SE** | 0.122 | 0.705 | 0.611 | 0.974 | -0.489 | 1.195 | 0.682 |
| **QG vs UC** | 2.806 | 0.76 | 2.809 | 0.893 | -0.002 | 1.173 | 0.998 |
| **QG vs NT** | 2.17 | 1.116 | 3.201 | 0.962 | -1.031 | 1.474 | 0.484 |
| **QG vs SE** | 2.265 | 0.914 | 1.485 | 0.897 | 0.78 | 1.28 | 0.542 |
| **YG vs UC** | 1.252 | 0.527 | 3.171 | 1.214 | -1.919 | 1.322 | 0.147 |
| **YG vs SE** | 1.11 | 1.417 | 0.474 | 0.782 | 0.636 | 1.619 | 0.695 |
| **DE vs UC** | 0.638 | 0.61 | 2.419 | 1.322 | -1.781 | 1.456 | 0.221 |
| **DE vs NT** | 1.9 | 1.089 | 0.12 | 0.967 | 1.78 | 1.457 | 0.222 |
| **DE vs WE** | 0.64 | 1.385 | -0.526 | 255.142 | 1.166 | 255.144 | 0.996 |
| **PE vs UC** | 1.496 | 1.006 | 2.357 | 132.95 | -0.861 | 132.954 | 0.995 |
| **SE vs UC** | -1.873 | 0.851 | -0.361 | 0.682 | -1.511 | 1.091 | 0.166 |

**UPDRS-Ⅲ**

|  | **Direct Indirect Difference** | | | | | | **p＞Z** |
| --- | --- | --- | --- | --- | --- | --- | --- |
|  | **Coefficient SE Coefficient SE Coefficient SE** | | | | | |  |
| **TC vs** **UC** | 4.137 | 3.25 | 1.253 | 3.597 | 2.884 | 4.847 | 0.552 |
| **TC vs** **NT** | 3.334 | 1.765 | 9.396 | 3.711 | -0.062 | 4.128 | 0.142 |
| **TC vs** **WE** | -0.701 | 2.777 | 0.157 | 7.906 | -0.858 | 8.463 | 0.919 |
| **TC vs** **SE** | 4.8 | 3.722 | 1.235 | 4.922 | 3.565 | 6.171 | 0.563 |
| **QG vs UC** | 2.531 | 2.145 | 5.418 | 4.355 | -2.887 | 4.855 | 0.552 |
| **QG vs NT** | 6.425 | 2.793 | 1.779 | 3.614 | 4.646 | 4.57 | 0.309 |
| **QG vs SE** | 2.1 | 4.211 | 5.665 | 4.512 | -3.565 | 6.171 | 0.564 |
| **YG vs UC** | 7.55 | 2.793 | 4.652 | 211.281 | 2.899 | 211.298 | 0.989 |
| **DE vs UC** | 4.832 | 2.482 | 5.656 | 185.098 | -0.824 | 185.113 | 0.996 |
| **PE vs UC** | 10.58 | 6.039 | 4.722 | 740.667 | 5.858 | 740.69 | 0.994 |
| **WE vs NT** | -6.413 | 3.928 | -3.627 | 3.917 | -2.786 | 5.53 | 0.614 |

**PDQ-39**

|  | **Direct Indirect Difference** | | | | | | **p＞Z** |
| --- | --- | --- | --- | --- | --- | --- | --- |
|  | **Coefficient SE Coefficient SE Coefficient SE** | | | | | |  |
| **TC vs DE** | -1.58 | 6.315 | 4.76 | 4.974 | -6.34 | 8.039 | 0.430 |
| **TC vs UC** | 7.279 | 3.575 | 1.324 | 6.661 | 5.955 | 7.647 | 0.436 |
| **TC vs SE** | 4.074 | 6.168 | -4.12 | 6.31 | 8.194 | 8.781 | 0.351 |
| **QG vs UC** | 9.91 | 3.166 | 10.838 | 5.91 | -0.928 | 6.724 | 0.890 |
| **QG vs WE** | 1.81 | 5.11 | 1.198 | 7.579 | 0.612 | 9.140 | 0.947 |
| **QG vs SE** | 4.589 | 4.838 | 3.582 | 6.898 | 1.007 | 8.402 | 0.905 |
| **YG vs UC** | 8.043 | 3.591 | 11.817 | 399.375 | -3.774 | 399.389 | 0.992 |
| **DE vs UC** | 2.026 | 4.209 | 6.221 | 5.474 | -4.196 | 6.903 | 0.543 |
| **DE vs NT** | -1.9 | 7.298 | -4.836 | 1022.8 | 2.936 | 1011.835 | 0.998 |
| **DE vs WE** | -5.2 | 5.972 | -4.488 | 6.91 | -0.712 | 9.132 | 0.938 |
| **SE vs UC** | -12.738 | 7.307 | -2.379 | 5.086 | -10.359 | 9.093 | 0.255 |

**HAMD**

|  | **Direct Indirect Difference** | | | | | | **p＞Z** |
| --- | --- | --- | --- | --- | --- | --- | --- |
|  | **Coefficient SE Coefficient SE Coefficient SE** | | | | | |  |
| **TC vs NT** | 7.026 | 1.931 | 0.247 | 232.773 | 6.779 | 232.78 | 0.977 |
| **QG vs UC** | 2.237 | 1.877 | -7.059 | 1169.072 | 9.296 | 1169.073 | 0.994 |
| **QG vs NT** | 0.78 | 2.326 | 7.59 | 234.243 | -6.81 | 234.254 | 0.977 |
| **QG vs WE** | 0.98 | 2.059 | -12.493 | 487.042 | 13.473 | 487.046 | 0.978 |
| **YG vs UC** | 1.82 | 2.228 | 17.005 | 2704.56 | -15.185 | 2704.561 | 0.996 |
