# Supplementary material for: Effects of mind–body exercise on motor function, quality of life, and depressive symptoms in patients with Parkinson’s disease: a systematic review and network meta-analysis
Source: Front Aging Neurosci. 2026 Oct 28;18:1982103. doi: 10.3389/fnagi.2026.1982103 (PMC13751929; doi:10.3389/fnagi.2026.1982103)
Supplement: Supplementary file 1 [file Data_Sheet_1.zip › Supplementary Material/Appendix 8-Results of loop inconsistency tests.docx]

# **Table 6. Results of loop inconsistency tests**

**BBS**

| **Loop** | **IF** | **SeIF** | **Z_value** | **P_value** | **CI_95** | **Loop_Heterog_tau2** |
| --- | --- | --- | --- | --- | --- | --- |
| **TC-QG-NT-WE** | 0.635 | 3.962 | 0.160 | 0.873 | (0.00,8.40) | 3.574 |
| **TC-QG-UC-NT** | 0.585 | 1.855 | 0.316 | 0.752 | (0.00,4.22) | 0.440 |
| **TC-QG-UC-WE** | 0.212 | 1.263 | 0.168 | 0.867 | (0.00,2.69) | 0.00 |

**TUG**

| **Loop** | **IF** | **SeIF** | **Z_value** | **P_value** | **CI_95** | **Loop_Heterog_tau2** |
| --- | --- | --- | --- | --- | --- | --- |
| **QG-DE-UC-NT** | 1.877 | 2.584 | 0.727 | 0.467 | (0.00,6.94) | 1.933 |
| **YG-UC-SE** | 1.723 | 1.227 | 1.404 | 0.160 | (0.00,4.13) | 0.093 |
| **TC-DE-UC-NT** | 1.532 | 1.906 | 0.804 | 0.422 | (0.00,5.27) | 1.288 |
| **QG-UC-SE** | 1.342 | 1.686 | 0.796 | 0.426 | (0.00,4.65) | 1.112 |
| **TC-YG-UC** | 1.203 | 0.828 | 1.453 | 0.146 | (0.00,2.83) | 0.200 |
| **TC-QG-NT-SE** | 0.990 | 2.026 | 0.489 | 0.625 | (0.00,4.96) | 1.132 |
| **TC-YG-SE** | 0.718 | 1.197 | 0.600 | 0.548 | (0.00,3.06) | 0.000 |
| **TC-UC-SE** | 0.588 | 1.867 | 0.315 | 0.753 | (0.00,4.25) | 1.832 |
| **TC-QG-UC-NT** | 0.346 | 1.954 | 0.177 | 0.860 | (0.00,4.18) | 0.094 |

**UPDRS-Ⅲ**

| **Loop** | **IF** | **SeIF** | **Z_value** | **P_value** | **CI_95** | **Loop_Heterog_tau2** |
| --- | --- | --- | --- | --- | --- | --- |
| **TC-QG-NT-SE** | 6.107 | 8.854 | 0.690 | 0.490 | (0.00,23.46) | 25.720 |
| **TC-QG-UC-NT** | 4.666 | 4.972 | 0.939 | 0.348 | (0.00,14.41) | 9.979 |
| **TC-NT-WE** | 2.188 | 6.706 | 0.326 | 0.744 | (0.00,15.33) | 22.777 |
| **TC-QG-UC-SE** | 1.279 | 3.633 | 0.352 | 0.725 | (0.008.40,) | 0.962 |

**PDQ-39**

| **Loop** | **IF** | **SeIF** | **Z_value** | **P_value** | **CI_95** | **Loop_Heterog_tau2** |
| --- | --- | --- | --- | --- | --- | --- |
| **TC-UC-SE** | 13.124 | 7.147 | 1.836 | 0.066 | (0.00,27.13) | 0.000 |
| **QG-UC-SE** | 8.698 | 12.132 | 0.717 | 0.473 | (0.00,32.48) | 56.472 |
| **TC-DE-UC** | 6.769 | 9.090 | 0.745 | 0.457 | (0.00,24.59) | 21.550 |
| **QG-DE-UC-WE** | 0.401 | 13.058 | 0.031 | 0.975 | (0.00,25.99) | 54.208 |
